# The prevalence of occupational exposure to noise: A systematic review and meta-analysis from the WHO/ILO Joint Estimates of the Work-related Burden of Disease and Injury

**DOI:** 10.1016/j.envint.2021.106380

**Published:** 2021-09

**Authors:** Liliane R. Teixeira, Frank Pega, Wagner de Abreu, Marcia S. de Almeida, Carlos A.F. de Andrade, Tatiana M. Azevedo, Angel M. Dzhambov, Weijiang Hu, Marta R.V. Macedo, Martha S. Martínez-Silveira, Xin Sun, Meibian Zhang, Siyu Zhang, Denise T. Correa da Silva

**Affiliations:** a Workers' Health and Human Ecology Research Center, National School of Public Health Sergio Arouca, Oswaldo Cruz Foundation, Rio de Janeiro, RJ, Brazil; b Department of Environment, Climate Change and Health, World Health Organization, Geneva, Switzerland; c Department of Epidemiology and Quantitative Methods in Health, National School of Public Health Sergio Arouca, Oswaldo Cruz Foundation, Rio de Janeiro, RJ, Brazil; d School of Medicine, Universidade de Vassouras, Vassouras, RJ, Brazil; e Workers' State Secretariat of Health, Rio de Janeiro, RJ, Brazil; f State Reference Center in Workers' Health, Rio de Janeiro, RJ, Brazil; g Department of Hygiene, Faculty of Public Health, Medical University of Plovdiv, Plovdiv, Bulgaria; h National Institute for Occupational Health and Poison Control, Center for Disease Control and Prevention, Beijing, People’s Republic of China; i Workers' Health Coordination, Oswaldo Cruz Foundation, Rio de Janeiro, RJ, Brazil; j Gonçalo Moniz Institute, Oswaldo Cruz Foundation, Salvador, BA, Brazil; k Zhejiang Provincial Center for Disease Control and Prevention, Hangzhou, People’s Republic of China; l Institute for Highway Engineering and Transport Planning, Graz University of Technology, Graz, Austria

**Keywords:** Global burden of disease, Systematic review, Prevalence, Occupational risk factor, Noise

## Abstract

**Background:**

The World Health Organization (WHO) and the International Labour Organization (ILO) are developing joint estimates of the work-related burden of disease and injury (WHO/ILO Joint Estimates), with contributions from a large network of individual experts. Evidence from mechanistic and human data suggests that occupational exposure to noise may cause cardiovascular disease. In this paper, we present a systematic review and meta-analysis of the prevalence of occupational exposure to noise for estimating (if feasible) the number of deaths and disability-adjusted life years from cardiovascular disease that are attributable to exposure to this risk factor, for the development of the WHO/ILO Joint Estimates.

**Objectives:**

We aimed to systematically review and meta-analyse estimates of the prevalence of occupational exposure to noise.

**Data sources:**

We searched electronic academic databases for potentially relevant records from published and unpublished studies, including Ovid Medline, PubMed, EMBASE, and CISDOC. We also searched electronic grey literature databases, Internet search engines, and organizational websites; hand-searched reference list of previous systematic reviews and included study records; and consulted additional experts.

**Study eligibility and criteria:**

We included working-age (≥15 years) workers in the formal and informal economies in any WHO Member and/or ILO member State, but excluded children (<15 years) and unpaid domestic workers. We included all study types with an estimate of the prevalence of occupational exposure to noise, categorized into two levels: no (low) occupational exposure to noise (<85dBA) and any (high) occupational exposure to noise (≥85dBA).

**Study appraisal and synthesis methods:**

At least two review authors independently screened titles and abstracts against the eligibility criteria at a first stage and full texts of potentially eligible records at a second stage, followed by extraction of data from qualifying studies. We combined prevalence estimates using random-effect meta-analysis. Two or more review authors assessed the risk of bias and the quality of evidence, using the RoB-SPEO tool and QoE-SPEO approach developed specifically for the WHO/ILO Joint Estimates.

**Results:**

Sixty-five studies (56 cross-sectional studies and nine cohort studies) met the inclusion criteria, comprising 157,370 participants (15,369 females) across 28 countries and all six WHO regions (Africa, Americas, Eastern Mediterranean, Europe, South-East Asia, and Western Pacific). For the main analyses, we prioritized the four included studies that surveyed national probability samples of general populations of workers over the 58 studies of workers in industrial sectors and/or occupations with relatively high occupational exposure to noise. The exposure was generally assessed with dosimetry, sound level meter, or official or company records; in the population-based studies, it was assessed with validated questions. Estimates of the prevalence of occupational exposure to noise are presented for all 65 included studies, by country, sex, 5-year age group, industrial sector, and occupation where feasible. The pooled prevalence of any (high) occupational exposure to noise (≥85dBA) among the general population of workers was 0.17 (95% confidence interval 0.16 to 0.19, 4 studies, 108,256 participants, 38 countries, two WHO regions, I^2^ 98%, low quality of evidence). Subgroup analyses showed that pooled prevalence differed substantially by WHO region, sex, industrial sector, and occupation.

**Conclusions:**

Our systematic review and meta-analysis found that occupational exposure to noise is prevalent among general populations of workers. The current body of evidence is, however, of low quality, due to serious concerns for risk of bias and indirectness. Producing estimates of occupational exposure to noise nevertheless appears evidence-based, and the pooled effect estimates presented in this systematic review are suitable as input data for the WHO/ILO Joint Estimates (if feasible).

***Protocol identifier*:** 10.1016/j.envint.2018.09.040

***PROSPERO registration number*:** CRD42018092272

## Highlights

• WHO and ILO are developing joint estimates of work-related burden of disease and injury.
• This paper synthesizes evidence on prevalence of occupational noise exposure.
• 65 studies were included (157,370 participants, 54 countries, six WHO regions).
• The prevalence of occupational noise exposure in workers was 0.17.

## 1 Background

The World Health Organization (WHO) and the International Labour Organization (ILO) are finalizing joint estimates of the work-related burden of disease and injury (WHO/ILO Joint Estimates) (Ryder, 2017). The organizations are estimating the numbers of deaths and disability-adjusted life years (DALYs) that are attributable to exposure to selected occupational risk factors. The WHO/ILO Joint Estimates are based on existing WHO and ILO methodologies for estimating the burden of disease for selected occupational risk factors (Ezzati et al., 2004, International Labour Organization, 1999, International Labour Organization, 2014, Pruss-Ustun et al., 2017). They expand these existing methodologies with estimation of the burden of several prioritized additional pairs of occupational risk factors and health outcomes. For this purpose, population attributable fractions (Murray et al., 2004) are being calculated for each additional risk factor-outcome pair, and these fractions are being applied to the total disease burden envelopes for the health outcome from the WHO *Global Health Estimates* for the years 2000–2016 (World Health Organization, 2019). Population attributable fractions are the proportional reduction in burden from the health outcome achieved by a reduction of exposure to the risk factor to zero.

The WHO/ILO Joint Estimates may include estimates of the burden of cardiovascular disease attributable to occupational exposure to noise, if feasible, as additional risk factor-outcome pairs whose global burden of disease has not previously been estimated. To select parameters with the best and least biased evidence for our estimation models, we conducted a systematic review and meta-analysis of studies of the prevalence of occupational exposure to noise (Teixeira et al., 2019). In the current paper, we present this systematic review and meta-analysis. WHO and ILO, supported by a large network of individual experts, have in parallel also produced a systematic review of studies estimating the effect of occupational exposure to noise on cardiovascular disease, defined as ischemic heart disease, stroke, and hypertension (Teixeira et al., 2021). The organizations are also concurrently conducting or have completed several other systematic reviews and meta-analyses on other additional risk factor-outcome pairs (Descatha et al., 2018, Descatha et al., 2020, Godderis et al., 2018, Hulshof et al., 2019, Li et al., 2018, Li et al., 2020, Mandrioli et al., 2018, Pachito et al., 2020, Paulo et al., 2019, Pega et al., 2020a, Rugulies et al., 2019, Teixeira et al., 2019, Tenkate et al., 2019; Hulshof et al., 2021b, Hulshof et al., 2021a; Teixeira et al., 2021). To our knowledge, these are the first systematic reviews and meta-analyses (with pre-published protocols) conducted specifically for an occupational burden of disease study (Mandrioli et al., 2018). The WHO and ILO’s joint estimation methodology and the WHO/ILO Joint Estimates are separate from these systematic reviews and will be described in more detail and reported elsewhere.

### 1.1 Rationale

To consider the feasibility of estimating the burden of cardiovascular disease attributable to occupational exposure to noise, and to ensure that potential estimates of burden of disease are reported in adherence with the guidelines for accurate and transparent health estimates reporting (GATHER) (Stevens et al., 2016), WHO and ILO require a systematic review of studies on the prevalence of relevant levels of occupational exposure to noise. The theoretical minimum risk exposure level is the exposure level that would result in the lowest possible population risk, even if it were not feasible to attain this exposure level in practice (Murray et al., 2004). These prevalence and effect estimates should be tailored to serve as parameters for estimating the burden of cardiovascular disease from occupational exposure to noise. In 2002, WHO carried out an assessment of the global burden of selected diseases (not including cardiovascular disease) from occupational exposure to noise, as part of a larger initiative to assess the impact of 25 risk factors in a standardized manner, in which noise related to work appears as one of the major risk factors to human health (Concha-Barrientos et al., 2004).

In general, the health consequences of a given level of occupational exposure to noise are likely to be similar, regardless of the country or region in which the exposure occurs. A review has therefore been carried out of all well-designed epidemiological studies that link occupational noise exposure to health outcomes, regardless of where the study was conducted (Concha-Barrientos et al., 2004). High levels of occupational exposure to noise remain a problem in all regions of the world, being one of the most common occupational risk factors (Concha-Barrientos et al., 2004, United States Department of Labor, 2013). According to recent evidence (Kerns et al., 2018), 22 million (25%) workers in the general population of the United States of America have a history of occupational exposure to noise (14% exposed in the last year). According to the 6th European Working Conditions Survey (EWCS) in 36 countries in Europe, 28% of workers in 2015 were exposed to excessive noise for at least a quarter of their time at work (Parent-Thirion et al., 2019).

The most serious effect of occupational exposure to noise is irreversible hearing impairment (Concha-Barrientos et al., 2004, Tomprette et al., 2019). This is explained by noise causing mechanical damage to the hair cells and other structures in the cochlea, impaired microcirculation in the inner ear and generation of free radicals, which in turn promote DNA damage, and protein and lipid peroxidation in those structures (Themann and Masterson, 2019). Additionally, according to the World Health Organization (2018), environmental noise exposure can have non-auditory effects on health, such as cardiovascular disease (van Kempen et al., 2018), noise annoyance (Guski et al., 2017), sleep disturbance (Basner and McGuire, 2018), metabolic disorders (van Kempen et al., 2018), cognitive impairment (Clark and Paunovic, 2018a), poor quality of life and mental ill-health (Clark and Paunovic, 2018b), and adverse birth outcomes (Dzhambov and Lercher, 2019, Nieuwenhuijsen et al., 2017). Adverse health effects have also been observed in the occupational environment where noise levels tend to be considerably higher (Themann and Masterson, 2019). For example, a recent systematic review with meta-analysis supported higher risk of some adverse pregnancy outcomes in women exposed to high occupational exposure to noise (Dzhambov et al., 2014). In another meta-analysis (Dzhambov and Dimitrova, 2017), a dose-response relationship was found between occupational exposure to noise and work-related injury risk, based however on a body of evidence the study authors judged to be of “very low” quality. According to Concha-Barrientos et al. (2004), there is limited evidence that occupational exposure to noise impairs performance and entails biochemical, immune, and birth weight effects. In this context, our systematic review and meta-analysis is highly warranted; it contributes a synthesis and assessment of the latest body of evidence from studies estimating the prevalence occupational exposure to noise.

### 1.2 Description of occupational exposure to noise

**Table 1:** Definitions of occupational exposure to noise, its levels, and the minimum risk exposure level.

Concept	Definition
Risk factor	Exposure to occupational noise is the exposure at the workplace to an unpleasant or unwanted sound
Risk factor levels	(1) Any occupational exposure to noise (≥85dBA) (2) No occupational exposure to noise (<85dBA)
Theoretical minimum risk exposure level	No occupational exposure to noise (<85dBA)

## 2 Objectives

To systematically review and meta-analyse evidence on the prevalence of any (high) occupational exposure to noise (≥85dBA) among working-age workers.

## 3 Methods

### 3.1 Developed protocol

**Fig. 1:**
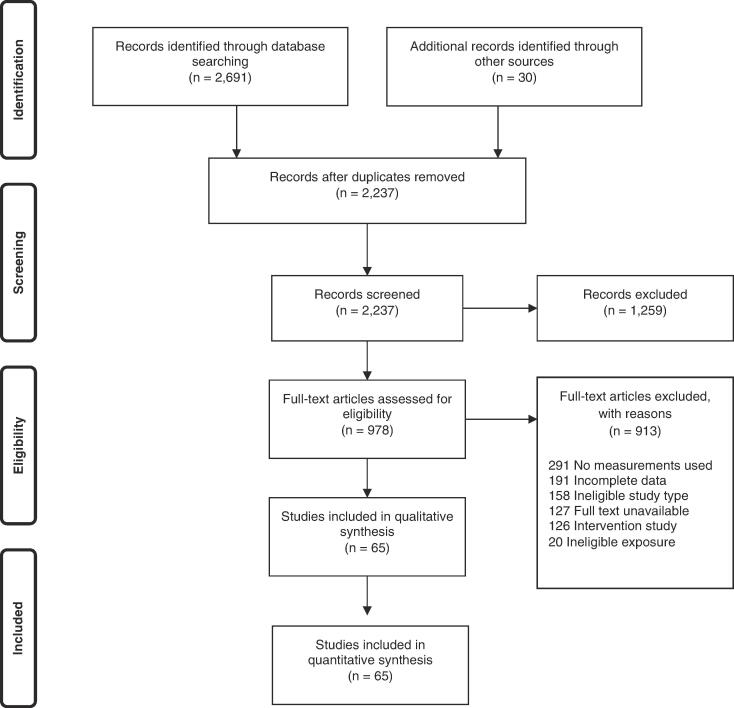
Flow diagram of study selection.

We applied the Navigation Guide systematic review methodology for systematic reviews in environmental and occupational health as our guiding methodological framework (Woodruff and Sutton, 2014), wherever feasible. The Navigation Guide applies established systematic review methods from clinical medicine, including standard Cochrane Collaboration methods for systematic reviews of interventions, to the field of environmental and occupational health to ensure systematic and rigorous evidence synthesis on environmental and occupational risk factors that reduces bias and maximizes transparency (Woodruff and Sutton, 2014). The need for further methodological development and refinement of the relatively novel Navigation Guide has been acknowledged (Woodruff and Sutton, 2014). Our systematic review may not map well to the Navigation Guide framework (Fig. 1 on page 1009 in Lam et al. (2016)), which is tailored to hazard identification and risk assessment. Nevertheless, steps 1–6 for the stream on human data can be applied to systematically review evidence on the prevalence of exposure to occupational risk factors.

We have registered the protocol in PROSPERO under CRD42018092272. This protocol adheres with the preferred reporting items for systematic review and meta-analysis protocols statement (PRISMA-P) (Moher et al., 2015, Shamseer et al., 2015), with the abstract adhering with the reporting items for systematic reviews in journal and conference abstracts (PRISMA-A) (Beller et al., 2013). Any modification of the methods stated in the protocol was registered in PROSPERO and is reported in the systematic review itself (this article). The systematic review is reported to align with the GATHER guidelines (Stevens et al., 2016).

### 3.2 Searched literature

#### 3.2.1 Information sources and search

##### 3.2.1.1 Electronic bibliographic databases

We searched the following electronic bibliographic databases:

(1) Ovid Medline (1 January 1946 to 21 March 2019 and updated on 25 May 2020)
(2) PubMed (1 January 1946 to 21 March 2019 and updated on 25 May 2020)
(3) Embase (1 January 1947 up to 29 March 2019 and updated on 25 May 2020)
(4) Web of Science (1 January 1945 up to 29 March 2019 and updated on 25 May 2020)
(5) Scopus (1 January 1966 up to 1 April 2019 and updated on 25 May 2020)
(6) Lilacs (1 January 1985 up to 1 April 2019 and updated on 25 May 2020)

The Ovid MEDLINE search strategy was presented in the published protocol (Teixeira et al., 2019). The full search strategies for all databases were revised by an information scientist and are presented in Appendix 1 in the Supplementary data. Searches were performed in electronic databases operated in the English language for most databases, and in Portuguese and Spanish for Lilacs. When we neared completion of the review, we conducted a top-up search of the Ovid Medline and PubMed databases on 31 January 2020 to capture the most recent publications (e.g., publications ahead of print). Deviations from the proposed search strategy and the actual search strategy are documented in Section 8.

##### 3.2.1.2 Electronic grey literature databases and complementary searches

We adapted the search syntax (Appendix 1 in the Supplementary data) to suit grey literature databases and searched the following electronic databases:

(1) CISDOC (up to 3 May 2018)
(2) OpenGrey (up to 3 May 2018)
(3) GreyLit (up to 3 May 2018)

##### 3.2.1.3 Internet search engines.

We also searched Google (www.google.com/) and Google Scholar (www.google.com/scholar/) on 3 May 2018 and screened the first 100 hits for potentially relevant records, as has previously been done in Cochrane Reviews (Pega et al., 2015, Pega et al., 2017).

##### 3.2.1.4 Organizational websites

The websites of the following seven international organizations and national government departments were also searched:

(1) International Labour Organization (www.ilo.org/)
(2) World Health Organization (www.who.int)
(3) European Agency for Safety and Health at Work (https://osha.europa.eu/en)
(4) Eurostat (www.ec.europa.eu/eurostat/web/main/home).
(5) China National Knowledge Infrastructure (http://www.cnki.net/)
(6) Finnish Institute of Occupational Health (https://www.ttl.fi/en/)
(7) National Institute of Occupational Safety and Health of the United States of America, using the institute’s data and statistics gateway (https://www.cdc.gov/niosh/data/)

##### 3.2.1.5 Hand-searching and expert consultation

We hand-searched for potentially eligible studies in:

• Reference lists of previous systematic reviews
• Reference lists of all included study records
• Study records published over the past 24 months in the three peer-reviewed academic journals from which we obtained the largest number of included studies
• Study records that have cited an included study record (identified in the Web of Science citation database)
• Collections of the review authors

Additional individual experts were contacted with a list of included studies, with the request to identify potentially eligible additional studies.

##### 3.2.1.6 National information searches

The review authors from the National Institute for Occupational Health and Poison Control of the Chinese Center for Disease Control and Prevention conducted searches of national and local bibliographic and grey literature databases in Chinese language for the People’s Republic of China.

### 3.3 Selected studies

Study selection was carried out using Covidence (Covidence systematic review software). All study records identified in the search were downloaded, and duplicates were identified and deleted. Afterwards, two review authors independently and in duplicate screened titles and abstracts (step 1), and then full texts (step 2) of potentially relevant records. A third review author resolved any disagreements between the two review authors. If a study record identified in the literature search was authored by a review author assigned to study selection or if an assigned review author was involved in the study, it was re-assigned to another review author for study selection. The study selection is presented in a flow chart, as per PRISMA guidelines (Liberati et al., 2009).

### 3.4 Eligibility criteria

The population and exposure criteria are described below.

#### 3.4.1 Types of populations

We included studies of working-age (≥15 years) workers in the formal and informal economies. Studies of children (aged < 15 years) and unpaid domestic workers were excluded. Participants residing in any WHO and/or ILO Member (or member) State and any industrial setting or occupation were included. We note that occupational exposure to noise may potentially have further population reach (e.g. through the release of noise from the workplace into the community) and acknowledge that the scope of our systematic reviews may not be able capture the environmental noise for nearby residents and its impacts on them.

#### 3.4.2 Types of exposures

We included studies that defined occupational exposure to noise in accordance with our standard definition (Table 1). We included studies with measures from any data source, including registry data. All studies of current occupational exposure to noise were included, whether measured objectively (e.g. by means of technology, such as a sound level meter), semi-subjectively (e.g. by means of measurements by experts, such as scientists with subject matter expertise), or based on self-reports by a worker, workplace administrator or manager. If a study reported both objective and subjective measures, we prioritized the objective measure. If a study reported both prevalence of current occupational exposure to noise and history of occupational exposure to noise (i.e., having ever been exposed to high noise levels at work), we prioritized current prevalence. This rule was applied to systematically prioritize prevalence measurements in included studies that collected two or more alternative prevalence measures, such as the National Health Interview Survey (NHIS) (Center for Disease Control and Prevention, 2007, Center for Disease Control and Prevention, 2014) and National Health and Nutrition Examination Survey (NHANES) (Center for Disease Control and Prevention and National Center for Health Statistics, 2020), as described in Appendix 2 in the Supplementary data.

#### 3.4.3 Types of studies

This systematic review included studies of any design reporting quantitative results, including cross-sectional studies. The studies had to be representative (within reason) of the relevant industrial sector(s), occupation(s) or the national population. We excluded qualitative, modelling, and case studies, as well as non-original studies without quantitative data (e.g. letters, commentaries, and perspectives).

Records published in any language were included. Most searches were conducted using English language terms, so that records published in any language that presented essential information (i.e. title and abstract) in English were included. If a record was written in a language other than those spoken by the authors of this review or those of other reviews (Descatha et al., 2018, Descatha et al., 2020, Godderis et al., 2018, Hulshof et al., 2019, Li et al., 2018, Li et al., 2020, Mandrioli et al., 2018, Pachito et al., 2020, Paulo et al., 2019, Pega et al., 2020a, Rugulies et al., 2019, Teixeira et al., 2019, Tenkate et al., 2019; Hulshof et al., 2021b, Hulshof et al., 2021a; Teixeira et al., 2021) in the series (i.e. Arabic, Bulgarian, Chinese, Danish, Dutch, English, French, Finnish, German, Hungarian, Italian, Japanese, Norwegian, Portuguese, Russian, Spanish, Swedish and Thai), then the record was translated into English. Published and unpublished studies were included. Studies conducted using unethical practices were excluded from the review.

#### 3.4.4 Types of prevalence measures

We included studies that reported a prevalence measure. Prevalence is the presence (and often the level) of an exposure to an occupational risk factor in each individual of the study population or in a representative sample at one particular time point (Porta, 2014). The prevalence (as here defined) is usually measured as the number of exposed persons (numerator) divided by the total number of persons (i.e., unexposed persons plus exposed persons) (denominator). It is usually reported as a fraction, in percentage points.

### 3.5 Extracted data

WHO and ILO developed a standard data extraction sheet. All data extractors piloted this sheet until there was convergence and agreement among them. Most data extractors participated in WHO’s online training for the use of the data extraction sheet. At a minimum, two review authors independently extracted the data on occupational exposure to noise, disaggregated by country, sex, age, and industrial sector and/or occupation. A third review author resolved conflicting extractions. Data were extracted on study characteristics (including study authors, study year, study country, participants, and exposure), study design (including study type, exposure measurement, and type of prevalence estimate), risk of bias (including missing data, as indicated by response rate and other measures), and study context. The prevalence estimates from included studies were entered and managed with the Stata computer software.

Data on potential conflict of interest were also extracted from the included studies, such as financial disclosures, funding sources, and authors’ affiliated organizations. A modification of a previous method was used to identify and assess undisclosed financial interests (Forsyth et al., 2014). If no financial disclosure and conflict of interest statements were provided, other records were searched from this study published in the 36 months prior to the included study record and in other publicly available repositories (Drazen et al., 2010a, Drazen et al., 2010b).

### 3.6 Requested missing data

We requested the missing data presented in Table 2.

**Table 2:** Description of missing data requested and received.

Study ID	Description of missing data	Person(s) from whom missing data were requested	Date of request(s)	Data received
Parent-Thirion 2015	Data disaggregated by sex, age, and occupation	Open-access database (https://www.eurofound.europa.eu/surveys/european-working-conditions-surveys-ewcs)	Downloaded on 30 January 2020	Downloaded on 30 January 2020
NHIS 2007 and NHIS 2014	Data disaggregated by sex, age, occupation, and industrial sector	Open-access database (www.cdc.gov/nchs/nhis/index.htm); Elizabeth Masterson, National Institute for Occupational Safety and Health of the United States of America, advised on the interpretation of measures of self-reported occupational exposure to noise	30 Jan 2020	31 Jan 2020
NHANES 1999–2004	Data disaggregated by sex, age, occupation, and industrial sector	Open-access database (www.cdc.gov/nchs/nhanes/index.htm); Elizabeth Masterson, National Institute for Occupational Safety and Health of the United States of America, advised on the interpretation of measures of self-reported occupational exposure to noise	30 Jan 2020	31 Jan 2020

### 3.7 Assessed risk of bias

We used the RoB-SPEO tool for assessing risk of bias in studies estimating the prevalence of exposure to occupational risk factors (Pega et al., 2020b). WHO and ILO, in collaboration with a Working Group of individual experts, developed this tool specifically for the systematic reviews conducted for the development of the WHO/ILO Joint Estimates. For each included study, two or more review authors independently assessed risk of bias with RoB-SPEO, and another review author resolved any conflicts between the individual assessments.

### 3.8 Conducted evidence synthesis (including meta-analysis)

If we found two or more studies with an eligible prevalence estimate, two or more review authors independently assessed the clinical heterogeneity (Deeks et al., 2011) of the studies in terms of population (WHO region and/or distribution by sex, age, industrial sector, and/or occupation) and exposure (definition, measurement methods, and level of exposure), following our protocol (Teixeira et al., 2019). If we judged two or more prevalence estimates to be sufficiently clinically homogenous, we pooled the prevalence estimates of these studies in a quantitative meta-analysis, using the inverse variance method with a random effects model. The output from these meta-analyses is a variance-weighted mean prevalence estimate, which is reported as a fraction. We assessed statistical heterogeneity using the I^2^ statistic. As recommended elsewhere (Higgins et al., 2019), we did not set specific ranges of I^2^ to indicate certain levels of statistical heterogeneity, and as described in Section 4.8.1 we expected statistical (and clinical) heterogeneity to be high. The meta-analysis was conducted in Stata 5.0. For our main meta-analysis, we prioritized the included studies that surveyed national probability samples of general populations of workers. We considered the studies of workers in industrial sectors and/or occupations with relatively high occupational exposure to noise as supporting evidence only, but made full use of these for additional analyses (see Section 3.9).

### 3.9 Conducted additional analyses

We conducted subgroup analyses by:

• WHO region (and/or country)
• Sex
• Industrial sector
• Occupation

We also planned to conduct subgroup analyses by age group, but did not do so because we did not access the data disaggregated by age.

For the subgroup analyses by sex, we prioritized the included studies that surveyed national probability samples of general populations of workers because these provided the prevalence that we were interested in. However, for the other subgroup analyses, we used all included studies (including those of workers in industrial sectors and/or occupations with relatively high occupational exposure to noise) to estimate prevalence in subgroups, such as defined industrial sectors and occupations, respectively, or if a subgroup had no data available from the prioritized evidence (i.e. general worker population studies). This may have introduced some between-subgroup heterogeneity in our subgroup analyses. Differences between subgroups were assessed with Chi^2^ tests; for each subgroup analysis, we report the p-value from the test and assume a p-value lower than 0.05 to indicate a significant subgroup difference.

No sensitivity or other analyses were conducted.

### 3.10 Assessed quality of evidence

We used the QoE-SPEO approach for assessing the quality of evidence in studies estimating the prevalence of exposure to occupational risk factors (Pega et al., submitted for publication). At least two review authors assessed quality of evidence. A third review author resolved conflicting ratings. WHO, supported by a Working Group of individual experts, developed the QoE-SPEO approach specifically for systematic reviews for the WHO/ILO Joint Estimates.

## 4 Results

### 4.1 Study selection

A flow diagram of the study selection is presented in Fig. 1. Sixty-five studies (56 cross-sectional studies and nine cohort studies) met the inclusion criteria, comprising 157,370 participants (15,369 females) across 28 countries and all six WHO regions (Africa, Americas, Eastern Mediterranean, Europe, South-East Asia, and Western Pacific). For 30 excluded studies that most closely resembled inclusion criteria, the reasons for exclusion are listed in Appendix 3 in the Supplementary data. All 65 included studies were included in the quantitative meta-analyses, with only four however included in the main meta-analysis.

### 4.2 Characteristics of included studies

The characteristics of the included studies are summarized in Table 3.

**Table 3:** Characteristics of included studies.

Study	Study population							Study type		
Study ID	Total number of study participants	Number of female study participants	Country of study population	Geographic location	Industrial sector (ISIC-4 code)	Occupation (ISCO-08 code)	Age (in years)	Study design	Study period	Follow-up period
Ahmed 2001	368	0	Kingdom of Saudi Arabia	Region	24, 35	8121, 7127	Mean = 32.09; s.d. = 7.70	Cross-sectional	1996–1999	Unclear
Attarchi 2012	331	0	Islamic Republic of Iran	Local	Unclear	Unclear	Range = 19–55; mean = 38.95	Cross-sectional	2010	Unclear
Attarchi 2013	471	0	Islamic Republic of Iran	Local	29	3341, 7131, 8211	Range = 20–58; mean = 32.91	Cross-sectional	2010–2011	Unclear
Bauer 1991	47,388	7,156	Austria	Local	Unclear	Unclear	Range = 25–55	Cross-sectional	1984–1986	36 months
Cantley 2015	9,220	989	United States of America	National	32	7223	Mean = 44.2; s.d. = 10.5	Cohort	1 Jan 2003–31 Dec 2008	72 months
Chang 2003	20	0	People's Republic of China	Local	45	1290	Mean = 39; s.d. = 7; mean = 45; s.d. = 8	Panel/Longitudinal	2000–2001	Unclear
Chang 2009	59	12	People's Republic of China	Local	15	7543, 8159, 7322, 9611, 4110, 9329	Mean = 34.6; s.d. = 7.7; mean = 40.3; s.d. = 5.8	Cross-sectional	2005–2006	Unclear
Chang 2012	281	33	People's Republic of China	Region	32	8121	Mean = 32.4; s.d. = 6.4	Cross-sectional	At the end of 2009	Unclear
Chen 2017	1,390	0	People's Republic of China	National	32	8189	Mean = 33.1; s.d. = 8.7	Cross-sectional	Unclear	Unclear
de Souza 2015	1,729	144	Brazil	Local	6	3134	<30; 30–34; 35–39; 40–44; 45–49; ≥50	Cross-sectional	6 months	6 months
Du 2007	3,279	Unclear	People's Republic of China	Region	13	8152	Range = 21–45; mean = 33.5	Cross-sectional	Unclear	Unclear
Guo 2012	60	50	People's Republic of China	Region	13	8159	Mean = 25.8; s.d. = 8.4	Cross-sectional	Feb 2010-Jan 2011	Unclear
He 2017	531	23	People's Republic of China	Region	20	Unclear	Unclear	Cross-sectional	2016	Unclear
Hong 1998	450	0	Republic of Korea	Region	51	3153	Unclear	Cross-sectional	1995	Unclear
Hu 2005	191	Unclear	People's Republic of China	Region	24	8121, 3343	Range = 20–53	Cross-sectional	Unclear	Unclear
Hughes 2013	503	27	United States of America	Local	51	3153	<25; 25–34; 35–44; 45–54; >55	Cohort	1 Jan 2001–31 Dec 2007	1 Jan 2001–31 Dec 2007
Inoue 2005	415	0	Japan	Region	17	8171	Unclear	Cross-sectional	Unclear	Unclear
Ivanovich 1994	249	249	Bulgaria	Local	61	2656	Mean = 34; s.d. = 9.0, Mean = 35.6; s.d. = 9.2, Mean = 40.4; s.d. = 6.5	Cross-sectional	Unclear	Unclear
Johnson 2006	313	35	Sweden	Local	96	7315	Unclear	Cross-sectional	2002–2006	2002–2006
Kock 2004	741	207	Denmark	Local	32	Unclear	Unclear	Cross-sectional	Unclear	Unclear
Kovacevic 2006	225	111	Montenegro	Local	13	8152	Mean = 42.5; s.d. = 8.5; mean = 39.2; s.d. = 8.5; mean = 33.1; s.d. = 9.0; mean = 32.9; s.d. = 7.2	Cross-sectional	Unclear	Unclear
Landen 2004	309	19	United States of America	Region	8	8111	18–29; 30–39; 40–49; 50–59 ; >60	Cross-sectional	Unclear	Unclear
Lee 1999	80	16	Singapore	Local	96	9626	Range = 17–44; mean = 24.6; s.d. = 6.8; range = 17–45; mean = 23.; s.d. = 5.9	Cross-sectional	Unclear	Unclear
Lee 2009	530	0	Republic of Korea	Local	24	Unclear	Range = 16–45; mean = 25.6	Cohort (retrospective)	1991–1999	9 years
Liu 2015	247	48	People's Republic of China	Region	32	8121	Mean = 38.0; s.d. = 11.0	Cross-sectional	Unclear	Unclear
Lv 2003	319	0	People's Republic of China	Region	Unclear	7232	Mean = 33.4; s.d. = 10.3	Cross-sectional	Unclear	Unclear
Maccà 2014	285	128	Italy	Local	13, 25, 32, 41	8159, 7211, 9313, 8114	Mean = 39.9; s.d. = 10	Cross-sectional	Unclear	Unclear
Melamed 1997	970	624	Israel	National	10, 13, 24, 26	8121, 8159, 8189, 8212, 8160	Women = 20–60; men 20–44, ≥45	Cross-sectional	Unclear	Unclear
Morata 1997	124	0	Brazil	Local	32	3139	Range = 21–58; mean = 33.8; s.d. = 8.5	Cross-sectional	Unclear	Unclear
Morata 1997	438	0	United States of America	Local	6	3134	Mean = 44.0; s.d. = 0.9; mean = 40.4; s.d. = 0.6; mean = 41.5; s.d. = 0.9; mean = 43.9; s.d. = 0.4; mean = 42.8; s.d. = 1.4; mean = 40.7; s.d. = 0.7	Cross-sectional	Jun 1989–1994	60 months
Nasir 2012	358	111	Malaysia	Local	51	3153	Range = 21–54; mean = 31.9; s.d. = 9.9	Cross-sectional	Nov 2008-March 2009	Unclear
NHIS 2014	34,059	18,494	United States of America	National	General working population	General working population	Range = 18–85+; mean = 49.37; s.d. = 17.99	Cross-sectional	2014	Unclear
NHIS 2007	20,800	11,221	United States of America	National	General working population	General working population	Range = 18-≥85; mean = 47.15; s.d. = 17.55	Cross-sectional	2007	Unclear
NHANES 1999–2004	9,721	509	United States of America	National	General working population	General working population	Range = 16-≥85; mean = 37.73; s.d. = 15.57	Cross-sectional	1999–2004	Unclear
Noweir 1984	2,458	0	Egypt	Other	13	7318	<25; 25–34; 35–44; 45–54; >55	Cross-sectional	1974 (data collected 1975–1976)	24 months
Nyarubeli 2018	326	Unclear	Tanzania	Local	25	7211, 7223	Unclear	Cohort (prospective)	Unclear	Unclear
Osibogun 2000	204	7	Nigeria	Local	13	8159	16–25; 26–35; 36–45; 46–55	Cross-sectional	Unclear	Unclear
Parent-Thirion 2015 (EWCS)	43,684	26,424	35 European countries: Albania, Austria, Belgium, Bulgaria, Croatia, Cyprus, Czech Republic, Denmark, Estonia, Finland, France, Republic of North Macedonia, Germany, Greece, Hungary, Ireland, Italy, Latvia, Lithuania, Luxembourg, Malta, Montenegro, Netherlands, Norway, Poland, Portugal, Romania, Serbia, Slovakia, Slovenia, Spain, Sweden, Switzerland, Turkey, United Kingdom of Great Britain and Northern Ireland	National	General working population	General working population	15–65	Cross-sectional. This is not one cross sectional study across all of Europe-but 35 separate cross-sectional studies with 35 separate data points.	Feb-Dec 2015	Not applicable
Pawlaczy-Luszczynska 2016	50	0	Poland	Region	31	8189	Range = 20–57; mean = 35; s.d. = 8.1	Cross-sectional	Unclear	Not applicable
Rabinowitz 2007	6,217	397	United States of America	Local	32	7223	Mean = 40.8; s.d. = 7.2	Cohort	1990–2004	7 years
Rachiotis 2006	145	16	Greece	Local	26	8212	≤ 40; > 40	Cross-sectional	Unclear	Unclear
Sancini 2014	191	0	Italy	Other	17	8171	Group A - mean = 38.1; s.d. = 7.7; group B - mean = 38.8; s.d. = 4; group C - mean = 38.8; s.d. = 3.6	Cross-sectional	Unclear	Unclear
Seixas 2001	59	0	United States of America	Local	43	7411	Mean = 34; s.d. = 9	Cross-sectional	4 months	4 months
Shakhatreh 2000	140	26	Jordan	Local	32	8159	Exposed group - mean = 38 male and 31.5 female; non-exposed group - mean = 39.7 male and 30.7 female	Cross-sectional	Unclear	Unclear
Shi 2009	1,170	0	People's Republic of China	Region	6	Not applicable	Unclear	Cross-sectional	March-May 2008	Unclear
Singh 2012	222	222	India	Region	24	7221	Exposed group - mean = 30.1; s.d. = 7.8; control group - mean = 32; s.d. = 8.9	Cross-sectional	Unclear	Unclear
Sliwinska-Kowalska 2004	906	124	Poland	Local	3	8350	Range = 20–66	Cross-sectional	Unclear	Unclear
Solecki 2008	44	Unclear	Unclear	Unclear	1	6111	Range = 33–65; mean = 48.6	Cross-sectional	1 year	Not applicable
Souza 2001	775	0	Brazil	Local	6	3134	≤ 38; > 38	Cross-sectional	1994	Unclear
Sriopas 2017	180	0	Thailand	Local	45	7212	20–30; >30–50	Cross-sectional	Unclear	1 year
Starck 1999	370	0	Unclear	Unclear	43	6210	Range 23–59; mean = 42.7; range 21–60; mean = 38.3	Cross-sectional	Unclear	Unclear
Stokholm 2013	11,395	2,887	Denmark	Local	Unclear	Unclear	<25; 25–34; 35–44; 45–54; 55–64; ≥65	Cohort (prospective)	1 Jan 2001–31 Dec 2007	2001–2007
Strauss 2014	40,123	0	South Africa	Local	7	8111	16–30; 31–40; 41–50; 51–60; 61–65	Cohort	2001–2008	8 years
Talbott 1999	308	0	United States of America	Local	29	8211	Range = 40–63; mean = 49.6; s.d. = 5.7; mean = 48.7; s.d. = 5.3	Cross-sectional	Since 1975	15 or more years of work
Toppila 2001	706	0	Finland	Region	Unclear	Unclear	Mean = 40; s.d. = 9	Cross-sectional	1995	Unclear
Vihma 1981	1,181	10	Finland	Local	16, 22, 24, 27	8121	Offset printers - mean = 30; relief printers - mean = 41	Cross-sectional	1976	1977–1980
Virkkunen 2005	6,005	0	Finland	National	96	8121	Beginning of the stud 40–56 ; end of the study 57–63	Cohort (prospective)	18 years	96 months-1985–1994
Whittaker 2014	238	Study group 5.22%; control group 25.2%	Nepal	Local	7, 55	3117, 9112	Median = 24.0; IQR = 16	Cross-sectional	Unclear	Unclear
Wu 1987	316	0	People's Republic of China	Local	43	9329	Unclear	Cross-sectional	Jun-Aug 1983	Jun-Aug 1983
Xiao 2008	1,906	458	People's Republic of China	Region	Unclear	Unclear	Range = 22–50	Cross-sectional	Unclear	Unclear
Xie 2015	98	15	People's Republic of China	Region	24	7223	Mean = 38.6; s.d. = 5.6	Cross-sectional	Unclear	Unclear
Xue 2018	1,813	0	People's Republic of China	Region	29	Not applicable	Range = 18–55	Cross-sectional	Unclear	Unclear
Yu 2017	6,297	Unclear	People's Republic of China	Region	24	7213	Unclear	Cohort (retrospective)	Unclear	Unclear
Yuan 2005	174	0	People's Republic of China	Region	32	7221, 4110	Mean = 36.5; s.d. = 9.4, Mean = 37.2; s.d. = 8.6	Cross-sectional	Unclear	Unclear
Zhao 1991	1,101	1,101	People's Republic of China	Local	13	8159	Mean = 38.5; s.d. = 8.1; mean = 37.2; s.d. = 8.6; mean = 33.9; s.d. = 8; mean = 33.9; s.d. = 8.2; mean = 35.5; s.d. = 8.4	Cross-sectional	8 Jul-10 Aug 1985	Unclear

Study	Exposure assessment							
Study ID	Exposure definition	Unit for which exposure was assessed	Mode of exposure data collection	Exposure assessment methods	Type of exposure measurement or estimate	Dates covered by exposure assessment (years)	Shortest and longest exposure period	Levels/intensity of exposure (specify unit)

Ahmed 2001	Equivalent continuous noise level (Leq/shift; mean Leq/shift and mean of the total noise immission level (TNIL)	Individual level	Technical device	Dosimeter	Prevalence	1996–1999	12h/day	>85dBA
Attarchi 2012	Leq (equivalent continuous noise level)	Individual level	Technical device	Sound level meter	Prevalence	2010	Unclear	Relevant category: >85dBA
Attarchi 2013	Leq (equivalent continuous noise level)	Individual level	Technical device	Sound level meter	Prevalence	2010–2011	8 working hours; repeated through 1 week	Relevant category: >85dBA
Bauer 1991	A-weighted sound-pressure	Individual level	Technical device	Sound level meter	Prevalence	1984–1986	Unclear	Relevant category: >85dBA
Cantley 2015	8h time weighted mean	Individual level	Administrative records	Dosimeter	Prevalence	2003–2008	Unclear	<82; 82–84.99; 85–87.99; ≥88dBA
Chang 2003	TWA (Leq)	Individual level	Technical device	Dosimeter	Prevalence	2000–2001	24h monitoring periods	Relevant category: >85dBA
Chang 2009	A-weighted (dBA) and time-weighted average	Individual level	Technical device	Dosimeter	Prevalence	Unclear	5 min/shift (8am-6pm)	≥80dBA
Chang 2012	A-weighted decibels (dBA)	Individual level	Technical device	Sound level meter, dosimeter	Prevalence	Dec 2009	50–120dBA to all subjects’ noise exposure with 5-min analyse over 8h	Relevant category ≥80dBA
Chen 2017	Equivalent continuous dBA weighted sound pressure levels (LEX,8h)	Group level	Technical device	Sound level meter	Prevalence	Unclear	Unclear	>80dBA
de Souza 2015	A-weighted sound level	Individual level	Technical device	Dosimeter	Prevalence	2007	Unclear	Relevant category: >85dBA
Du 2007	A-weighted equivalent sound level (LAeq), peak sound level	Group level	Technical device	Dosimeter	Prevalence	Unclear	Unclear	Relevant category: >85dBA
Guo 2012	A sound level and sound pressure level (SPL), cumulative noise exposure (CNE)	Group level	Technical device	Dosimeter	Prevalence	Unclear (covered 24h on a day after Feb 2010)	Unclear	Relevant category: >85dBA
He 2017	A-weighted equivalent sound level (LAeq)	Group level	Technical device	Dosimeter	Prevalence	Unclear	Unclear	Relevant category: >85dBA
Hong 1998	Time-weighted average	Individual level	Technical device	Dosimeter	Prevalence	Unclear	Daily 8h TWA	Relevant category: >85dBA
Hu 2005	A-weighted equivalent sound level (LAeq)	Group level	Technical device	Dosimeter	Prevalence	Unclear	Unclear	Relevant category: >85dBA
Hughes 2013	Equivalent Cumulative Level (ECL)	Group level	Administrative records	Registry data (industrial hygiene noise exposure monitoring data)	Prevalence	Jan 1, 2001 to Dec 31, 2007	Unclear	> 85dBA
Inoue 2005	A-weighted sound level	Group level	Administrative records	Sound level meter	Sound level measurement	Twice per year	Unclear	> 92dBA on average
Ivanovich 1994	Equivalent noise level (Leq)	Individual level	Technical device	Sound level meter	Prevalence	Unclear	15 min intervals during normal working activities	>85dBA
Johnson 2006	8h Level Equivalent Dosimeter Measurements	Individual level	Technical device	Dosimeter	Prevalence	4 years	One full shift (at least)	>85dBA
Kock 2004	A-weighted equivalent sound level (LAeq)	Individual level	Technical device	Dosimeter	Prevalence	Unclear (covered 24h on a day after 1 Aug 2001)	Always 24 h	>85dBA
Kovacevic 2006	Equivalent noise levels (Leq)	Group level	Technical device	Noise level analyzer	Prevalence	Unclear	One shift (8h)	Unclear
Landen 2004	Full shift, TWA (dBA)	Individual level	Technical device	Dosimeter	Prevalence	Unclear	Mean = 9h43min; s.d. = 1h11min	> 80dBA
Lee 1999	Time Weighted Average (TWA) level (dBA)	Individual level	Technical device	Dosimeter	Prevalence	Unclear	Whole work shift (7/8pm to 3am)	Relevant category: >85dBA
Lee 2009	TWA (dBA)	Individual level	Technical device	Dosimeter	Incidence	9 years	6h/day on demand	Relevant category: >85dBA
Liu 2015	Environmental noise levels and time-weighted noise level	Individual level	Technical device	Sound level meter, dosimeter	Prevalence	Unclear	Unclear	Relevant category: >85dBA
Lv 2003	The 8h equivalent continuous A sound level of each subject (LAeq.8h)	Individual level	Technical device	Unclear	Prevalence	Unclear (covered 24h on a day)	10h	Relevant category: >85dBA
Maccà 2014	dBA Lep,d	Individual level	Administrative records (supplied by the management of factories)	Sound level meter	Prevalence	Unclear	Unclear	80–100.7dBA
Melamed 1997	Time-weighted equivalent noise level	Group level	Technical device	Ambient noise levels	Prevalence	Twice each day	1/2h	Relevant category: >85dBA
Morata 1997	LAEq values in dBA	Individual level	Technical device	Dosimeter	Prevalence	Jun 1989	Full shift − 8h shifts	≤80dBA; 81-85dBA; 86-90dBA; ≥91dBA
Morata 1997	Time weighted average	Group level	Technical device	Unclear	Prevalence	Unclear	2-3h/day in some departments and 8h/shift at others	78-101dBA, mean = 87dBA; 71-98dBA, mean = 88dBA
Nasir 2012	Time-weighted average	Individual level	Technical device	Dosimeter	Prevalence	>5 years	Occupational exposure to noise >5years, 8h time-weighted average	<85 dBA; 85-89dBA; ≥ 90dBA
NHIS 2014	Exposure to “loud” or “very loud” sounds/ noise	Individual level	Self-reported	Questionnaire	Prevalence	Last 12 months	≥ 4 h/day, several days/week	“Loud” or “very loud” noise (approx. >85/90dBA)
NHIS 2007	Exposure to “loud” sounds/ noise	Individual level	Self-reported	Questionnaire	Prevalence	Last 12 months	≥ 4 h/day, several days/week	“Loud” noise (approx. >85dBA)
NHANES 1999–2004	Exposure to “loud” noise	Individual level	Self-reported	Questionnaire	Prevalence	“Currently“	≥ 3 months	“Loud” noise (approx. >85dBA)
Noweir 1984	Average value of the A-weighted sound pressure level and sound pressure levels	Group level	Technical device	Sound level meter	Prevalence	Unclear	Unclear	≥90dBA
Nyarubeli 2018	A-weighted equivalent noise level and the C-weighted peak noise level	Individual level	Technical device	Dosimeter	Incidence	Jun 2016-Jun 2017	Unclear	“Loud” noise (approx. >85dBA)
Osibogun 2000	Leq 60sec noise (for rapidly fluctuating noise) in the A-Weighted network and slow meter response	Group level	Technical device	Sound level meter	Prevalence	Unclear	Unclear	<85dBA; 85-90dBA; >90dBA
Parent-Thirion 2017 (EWCS)	Exposure to “Noise so loud that you would have to raise your voice to talk to people”. Responses ‘never or almost never’ were classified as ‘not occupationally exposed to noise’ and ‘Around 1/4 of the daytime’ or more as “occupationally exposed’	Individual level	Face-to-face computer-assisted personal interview	Self-reports	Prevalence	Feb-Dec 2015	At least half of the time at work	“Loud” noise (approx. > 85dBA)
Pawlaczy-Luszczunska 2016	Daily noise exposure level (LEX,8h), maximum A-weighted sound pressure level (with time constant Slow) (LAmax) and peak C-weighted sound pressure level (LCpeak)	Group level	Technical device	Sound level meter	Prevalence	Unclear	3–14 years	LEX,8h: 82.7–94.8dBA, LAmax; 91.9–108.6dBA; LCpeak: 111.5–139.3dBA
Rabinowitz 2007	Time Averaged Equivalent (Leq)	Individual level	Administrative records	Dosimeter	Incidence	14 years	Unclear	Unclear
Rachiotis 2006	Equivalent continuous Sound Level (Leq dB (A))	Group level	Technical device	Sound level meter	Prevalence	Uncl	Unclear	91 (dBA, Leq)
Sancini 2014	Equivalent continuous level noise (LAeq)	Individual level	Technical device	Sound level meter	Prevalence	Unclear	8h shift	Relevant category: >85dBA
Seixas 2001	Losha (TWA), Leq and Lpeak	Individual level	Technical device	Dosimeter	Prevalence	4 months	Workday	> 85dBA
Shakhatreh 2000	dB (A)	Group level	Technical device	Sound level meter	Prevalence	Unclear	Unclear	Unclear
Shi 2009	Sound pressure level (SPL);A-weighted equivalent sound level (LAeq)	Group level	Technical device	Dosimeter	Prevalence	Unclear	8h	>85dBA
Singh 2012	A weighted (Leq) ambient noise and time-weighted average dose	Individual level	Technical device	Dosimeter	Prevalence	Unclear	8h	≥90dBA
Sliwinska-Kowalska 2004	A-weighted equivalent sound pressure level	Group level	Technical device	Sound level meter, sound pressure level meter	Prevalence	Unclear	Unclear	Relevant category: >85dBA
Solecki 2008	8h workday (LEX, 8h)	Individual level	Technical device	Dosimeter and sound level meter	Prevalence	Unclear	Shortest-11 years	lifetime dose (noise emission level) 103.9 dBA; s.d.=1.09 dBA), median= 104 dBA; daily noise exposure level LEX,8h=90.2 dBA, noise exposure (per year): 2.54–4.16 Pa2xh, mean = 3.35; s.d.=1.27) Pa2xh
Souza 2001	Cumulative exposure to noise (dBA)	Individual level	Administrative records	Dosimeter	Prevalence	10 years	Unclear	Relevant category: >85dBA
Sriopas 2017	Time-weighted average of 8h	Individual level	Technical device	Dosimeter	Prevalence	At least 1 year	Working time for 8h for one time	Relevant category: >85dBA
Starck 1999	A-weighted equivalent level	Group level	Technical device	Unclear	Prevalence	Unclear	Unclear	Relevant category: >85dBA
Stokholm 2013	Full-shift noise exposure levels (*L*AEq values)	Individual level	Technical device	Dosimeter	Unclear	2001 and 2009–2010	Unclear	70-86dBA
Strauss 2014	Time-weighted average	Individual level	Administrative records	Dosimeter	Incidence	2001–2008	Unclear	Relevant category: >85dBA
Talbott 1999	Time-weighted average and Cumulative Exposure Level (Expc)	Individual level	Administrative records	Registry data	Prevalence	1975–1986	Unclear	Plant 1: 89–101.6dBA; Plant 2: ≤83dBA
Toppila 2001	10 min samples calculated A-weighted levels weighted noise exposure level	Individual level	Technical device	Datalogger calibrated	Prevalence	Unclear	Unclear	<90dBA; <100dBA; >100dBA
Vihma 1981	Mean noise level (A filter and predominantly the 200-ms time constant) and Impulse noise (the 35-ms time constant)	Group level	Technical device	Noise meter	Prevalence	Unclear	Unclear	Unclear
Virkkunen 2005	Mean level of exposure among the exposed (level L) by occupation and period	Group level	Administrative records	Registry data (FINJEM database) exposures to continuous and impulse noise	Incidence	18 years	1985–1994 (9 years follow-up)	1 = unexposed, 2 = exposed to 80–85dBA, 3 = exposed to >85dBA
Whittaker 2014	8h equivalent level	Individual level	Technical device	Workplace noise assessment	Prevalence	Unclear	1h	Relevant category: >85dBA
Wu 1987	A-weighted sound level	Group level	Technical device	Sound level meter	Prevalence	Jun-Aug 1983	Unclear	Relevant category: >85dB
Xiao 2008	A-weighted equivalent sound level (LAeq), peak sound level	Group level	Technical device	Dosimeter	Prevalence	Unclear	Unclear	Relevant category: >85dBA
Xie 2015	LAeq8h	Individual level	Technical device	Dosimeter	Prevalence	Unclear	Unclear	Relevant category: >85dBA
Xue 2018	Equivalent continuous A-weighted sound pressure level, LAeq8h	Individual level	Technical device	Dosimeter	Prevalence	Unclear	Unclear	Relevant category: >85dBA
Yu 2017	Cumulative noise exposure	Individual level	Technical device	Dosimeter	Prevalence	Unclear	Unclear	Relevant category: >85dBA
Yuan 2005	A-weighted equivalent sound level (LAeq)	Group level	Technical device	Dosimeter	Prevalence	Unclear	Unclear	Relevant category: >85dBA
Zhao 1991	Time-weighted average	Group level	Technical device	Sound level meter	Prevalence	Unclear	8h	Relevant category: >85dBA

Study	Co-exposure		Prevalence estimate				
Study ID	Exposure definition	Potential co-exposure with other occupational risk factors	Prevalence estimate type	Definition of numerator population	Number of study participants in exposed group	Definition of denominator population	Number of study participants in unexposed group
Ahmed 2001	Equivalent continuous noise level (Leq/shift; mean Leq/shift and mean of the total noise immission level (TNIL)	None	Prevalence	Number of workers above 85 dBA	202	All inspected workers	99
Attarchi 2012	Leq (equivalent continuous noise level)	Yes	Prevalence	Number of workers above 85 dBA	167	All inspected workers	164
Attarchi 2013	Leq (equivalent continuous noise level)	Mixed organic solvents	Prevalence	Number of workers above 85 dBA	347	All inspected workers	124
Bauer-1991	A-weighted sound-pressure	No	Prevalence	Number of workers above 85 dBA	45,154	All inspected workers	2,234
Cantley 2015	8h time weighted mean	No	Prevalence	Number of workers above 85 dBA	2,422	All inspected workers	6,798
Chang-2003	TWA (Leq)	No	Prevalence	Number of workers above 85 dBA	15	All inspected workers	5
Chang 2009	A-weighted (dBA) and time-weighted average	Organic solvents (DMF and toluene)	Prevalence	Number of workers above 85 dBA	42	All inspected workers	17
Chang 2012	A-weighted decibels (dBA)	Unclear	Prevalence	Number of workers above 85 dBA	68	All inspected workers	120
Chen 2017	Equivalent continuous dBA weighted sound pressure levels (LEX,8h)	Unclear	Prevalence	Number of workers above 85 dBA	900	All inspected workers	490
de Souza 2015	A-weighted sound level	Unclear	Prevalence	Number of workers above 85 dBA	470	All inspected workers	1,259
Du 2007	A-weighted equivalent sound level (LAeq), peak sound level	Unclear	Prevalence	Number of workers above 85 dBA	1,342	All inspected workers	1,937
Guo 2012	A sound level and sound pressure level (SPL), cumulative noise exposure (CNE)	No	Prevalence	Number of workers above 85 dBA	60	All workers in the position	0
He 2017	A-weighted equivalent sound level (LAeq)	No	Prevalence	Number of workers above 85 dBA	165	All inspected workers	348
Hong 1998	Time-weighted average	None	None	Number of workers above 85 dBA	255	All inspected workers	195
Hu 2005	A-weighted equivalent sound level (LAeq)	No	Prevalence	Number of workers above 85 dBA	123	All inspected workers	68
Hughes 2013	Equivalent Cumulative Level (ECL)	toluene, styrene, xylene, benzene	Prevalence	Number of workers above 85 dBA	368	All inspected workers	135
Inoue 2005	A-weighted sound level	No	Prevalence	Number of workers above 85 dBA	242	All inspected workers	173
Ivanovich 1994	Equivalent noise level (Leq)	Unclear	Prevalence	Number of workers above 85 dBA	81	All inspected workers	168
Johnson 2006	8h Level Equivalent Dosimeter Measurements.	Styrene	Prevalence	Number of workers above 85 dBA	146	All inspected workers	167
Kock 2004	The A-weighted equivalent sound level (LAeq)	No	Prevalence	Number of workers above 85 dBA	254	All workers in the industrial sector	487
Kovacevic 2006	Equivalent noise levels (Leq)	No	Prevalence	Number of workers above 85 dBA	111	All inspected workers	114
Landen 2004	Full shift TWA (dBA)	No	Prevalence	Number of workers above 85 dBA	213	All inspected workers	96
Lee 1999	Time Weighted Average (TWA) level (dBA)	No	Prevalence	Number of workers above 85 dBA	40	All inspected workers	37
Lee 2009	TWA (dBA)	No	Incidence	Number of workers above 85 dBA	133	All inspected workers	397
Liu 2015	Environmental noise levels and time-weighted noise level	Total volatile organic compounds (TVOCs)	Prevalence	Number of workers above 85 dBA	100	All inspected workers	147
Lv 2003	The 8h equivalent continuous A sound level of each subject (LAeq.8h)	No	Prevalence	Number of workers above 85 dBA	290	All workers in the position	29
Maccà 2014	dBA Lep,d	No	Prevalence	Number of workers above 85 dBA	137	All inspected workers	148
Melamed 1997	Time-weighted equivalent noise level	Unclear	Prevalence	Number of workers above 85 dBA	205	All inspected workers	765
Morata 1997	LAEq values in dBA	Solvents	Prevalence	Number of workers above 85 dBA	74	All inspected workers	50
Morata 1997	Time weighted average	Solvents	Prevalence	Number of workers above 85 dBA	189	All inspected workers	249
Nasir 2012	Time-weighted average	No	Prevalence	Number of workers above 85 dBA	136	All inspected workers	222
NHIS 2014	Exposure to “loud” or “very loud” sounds/ noise	No	Prevalence	Number of workers reporting “loud” or “very loud” noise	3,543	All inspected workers	30,502
NHIS 2007	Exposure to “loud” sounds/ noise	No	Prevalence	Number of workers reporting “loud” noise	2,094	All inspected workers	18,706
NHANES 1999–2004	Exposure to “loud” noise	No	Prevalence	Number of workers reporting “loud” noise	1,523	All inspected workers	8,198
Noweir 1984	Average value of the A-weighted sound pressure level and sound pressure levels	None	Prevalence	Number of workers above 90 dBA	1,404	All inspected workers	1,054
Nyarubeli 2018	A-weighted equivalent noise level and the C-weighted peak noise level	None	Incidence	Number of workers above 85 dBA	293	All inspected workers	33
Osibogun 2000	Leq 60sec noise (for rapidly fluctuating noise) in the A-weighted network and slow meter response	No	Prevalence	Number of workers above 85 dBA	116	All inspected workers	71
Parent-Thirion 2017 (EWCS)	Exposure to “Noise so loud that you would have to raise your voice to talk to people”. Responses ‘never or almost never’ were classified as ‘not occupationally exposed to noise’ and ‘Around 1/4 of the day time’ or more as “occupationally exposed’	Unclear	Prevalence	All participants reporting ‘exposure’ for at least 4 h/day	7,831	All participants in the study	35,805
Pawlaczy-Luszczunska 2016	Daily noise exposure level (LEX,8h), maximum A-weighted sound pressure level (with time constant Slow) (LAmax) and peak C-weighted sound pressure level (LCpeak)	Organic solvents, tobacco smoking	Prevalence	Number of workers above 85 dBA	50	All inspected workers	0
Rabinowitz 2007	Time Averaged Equivalent (Leq)	No	Incidence	Number of workers above 85 dBA	1,759	All inspected workers	4,458
Rachiotis 2006	Equivalent continuous Sound Level (Leq dB (A))	Unclear	Prevalence	Number of workers above 85 dBA	94	All inspected workers	51
Sancini 2014	Equivalent continuous level noise (LAeq)	No	Prevalence	Number of workers above 85 dBA	72	All inspected workers	119
Seixas 2001	Losha (TWA), Leq and Lpeak.	No	Prevalence	Number of workers above 85 dBA	40	All inspected workers	19
Shakhatreh 2000	dB (A)	No	Prevalence	Number of workers above 85 dBA	22	All inspected workers	70
Shi 2009	Sound pressure level (SPL);A-weighted equivalent sound level (LAeq)	Unclear	Prevalence	Number of workers above 85 dBA	385	All inspected workers	785
Singh 2012	A weighted (Leq) ambient noise and time-weighted average dose	None declared	Prevalence	Number of workers above 85 dBA	165	All inspected workers	57
Sliwinska-Kowalska 2004	A-weighted equivalent sound pressure level	Organic Solvents mixture	Prevalence	Number of workers above 85 dBA	701	All inspected workers	205
Solecki 2008	8h work day (LEX, 8h)	Unclear	Prevalence	Number of workers above 85 dBA	44	All inspected workers	0
Souza 2001	Cumulative exposure to noise (dBA)	No	Prevalence	Number of workers above 85 dBA	585	All inspected workers	190
Sriopas 2017	Time-weighted average of 8h	No	Prevalence	Number of workers above 85 dBA	113	All inspected workers	67
Starck 1999	A-weighted equivalent level	Smoke	Prevalence	Number of workers above 85 dBA	46	All inspected workers	324
Stokholm 2013	Full-shift noise exposure levels (*L*AEq values)	No	Unclear	Number of workers above 85 dBA	6,262	All inspected workers	5,133
Strauss 2014	Time-weighted average	No	Incidence	Number of workers above 85 dBA	33,961	All inspected workers	6,162
Talbott 1999	Time-weighted average and Cumulative Exposure Level (Expc)	No	Prevalence	Number of workers above 85 dBA	62	All inspected workers	246
Toppila 2001	10min samples calculated A-weighted levels weighted noise exposure level	No	Prevalence	Number of workers above 85 dBA	706	All inspected workers	0
Vihma 1981	Mean noise level (A filter and predominantly the 200ms time constant) and Impulse noise (the 35ms time constant)	No	Prevalence	Number of workers above 85 dBA	404	All inspected workers	777
Virkkunen 2005	Mean level of exposure among the exposed (level L) by occupation and period	Impulsive noise	Incidence	Number of workers above 85 dBA	2,958	All inspected workers	3,047
Whittaker 2014	8h equivalent level	No	Prevalence	Number of workers above 85 dBA	115	All inspected workers	123
Wu 1987	A-weighted sound level	No	Prevalence	Number of workers above 85 dBA	158	All inspected workers	158
Xiao 2008	A-weighted equivalent sound level (LAeq), peak sound level	No	Prevalence	Number of workers above 85 dBA	953	All inspected workers	953
Xie 2015	LAeq.8h	Unclear	Prevalence	Number of workers above 85 dBA	98	All inspected workers	0
Xue 2018	Equivalent continuous A-weighted sound pressure level, LAeq8 h	Unclear	Prevalence	Number of workers above 85 dBA	700	All inspected workers	1,113
Yu 2017	Cumulative noise exposure	Unclear	Prevalence	Number of workers above 85 dBA	6,112	All inspected workers	185
Yuan 2005	A-weighted equivalent sound level (LAeq)	No	Prevalence	Number of workers above 85 dBA	88	All inspected workers	86
Zhao 1991	Time-weighted average	No	Prevalence	Number of workers above 85 dBA	886	All inspected workers	215

#### 4.2.1 Study type

Fifty-six included studies were cross-sectional and nine were cohort studies.

#### 4.2.2 Population studied

The included studies captured 157,370 workers (15,369 females, 141,675 males). Thirty-one and 33 studies examined male, and both female and male workers, respectively. No study of females only was found.

By WHO region, most studies examined populations in the Western Pacific (24 studies from 6 countries), followed by populations in Europe (16 studies from 10 countries), Americas (10 studies from 2 countries), Eastern Mediterranean (5 studies from 4 countries), and South-East Asia (3 studies from 3 countries), with fewest studies found from Africa (3 studies from 3 countries). The most commonly studied countries were the People's Republic of China (19 studies) and the United States of America (7 studies).

The industrial sectors most commonly studied were mining and quarrying, other manufacturing (7 studies), manufacture of basic metals (6 studies), manufacture of motor vehicles, trailers and semi-trailers (4 studies) and manufacture of textiles (4 studies).

The occupations included in most studies were plant and machine operators and assemblers (19 studies), followed by craft and related trades workers (10 studies), technicians and associate professionals (seven studies), metal processing plant operators (four studies), and petroleum and natural gas refining plant operators and aircraft pilots and related associate professionals (three studies each).

Half of all studies (31) measured occupational exposure to noise using dosimetry. Nineteen used direct measurements collected with sound level meters. The remaining 12 studies measured exposure indirectly (e.g. by means of proxy through occupation); this included the four studies of general populations of workers (Center for Disease Control and Prevention, 2007, Center for Disease Control and Prevention, 2014, Center for Disease Control and Prevention, 2020, Parent-Thirion et al., 2019) that used self-reported measurements collected in surveys (see Table 3 for description of the survey items).

All studies measures enabled us to differentiate “any” from “no” occupational exposure to noise as per our pre-specified levels (see Table 1).

### 4.3 Risk of bias within studies

The tables of risk of bias of each included study are presented in Appendix 4 in the Supplementary data.

#### 4.3.1 Cohort studies

**Fig. 2:**
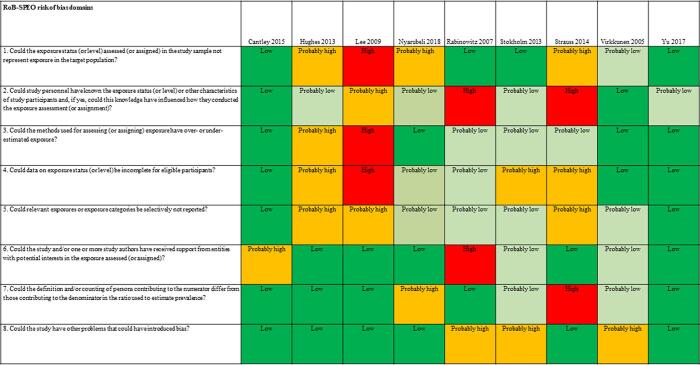
Summary of risk of bias, cohort studies.

Fig. 2 presents an overview of risk of bias in included cohort studies. Each study was rated along RoB-SPEO’s eight risk of bias domains (Pega et al., 2020b).

##### 4.3.1.1 Bias in selection of participants into the study

We assessed risk of bias in this domain based on whether the groups being compared were the same in all relevant ways (or as close to this as possible) apart from the exposure. Of the nine included studies, the risk of selection bias was rated to be high for one study due to the study records not providing sufficient detail for target population, study sample, criteria for eligibility for individuals to participate in the study, sampling, recruitment and enrolment procedures, rate of participation in the study, and rate of participation in the exposure assessment (Lee et al., 2009). Three studies were rated as ”probably high” because indirect evidence suggests that inclusion/exclusion criteria, recruitment and enrolment procedures, and participation/response rates differed across groups exposure (Hughes and Hunting, 2013, Strauss et al., 2014). One study was rated as “probably low” because the authors declared the inclusion of white-collar analyses would impact the results (Virkkunen et al., 2005) (Fig. 2).

##### 4.3.1.2 Performance bias

For the eight included studies, this bias was rated “high” for two studies due to exposure assessors and study personnel not being blinded or being incompletely blinded, and the exposure assessment was likely to have been influenced by the lack of blinding (e.g., exposure was systematically assessed differentially for subgroups defined by participant characteristics) (Rabinowitz et al., 2007, Strauss et al., 2014). For one study the risk was deemed “probably high” because the information on blinding was insufficient to permit a rating of risk of bias (Lee et al., 2009). Four studies were rated as “probably low” because indirect evidence suggests that the exposure assessors and study personnel were adequately blinded or blinding was unlikely to influence exposure assessment (Hughes and Hunting, 2013, Stokholm et al., 2013, Yu et al., 2017) (Fig. 2).

##### 4.3.1.3 Bias due to exposure misclassification

For the eight included studies, this bias was rated high for one study, due to direct evidence suggesting that the method adopted in the study for the exposure assessment and assignment does not produce valid, accurate and reliable exposure data, ideally based on direct quantitative exposure measures (Lee et al., 2009); one “probably high” because the information on blinding is insufficient to permit a rating of risk of bias (Lee et al., 2009); another was rated as “probably high” because as described in the report, noise exposure data may overestimate the true exposure to noise among the subjects due to use of hearing protection as per occupational standards and HCPs regulations (Hughes and Hunting, 2013); and three studies were rated as “probably low” because insufficient information existed about the exposure assessment and assignment method to permit rating the risk of bias (Rabinowitz et al., 2007, Stokholm et al., 2013, Strauss et al., 2014) (Fig. 2).

##### 4.3.1.4 Bias due to incomplete exposure data

For the eight included studies, this bias was rated “high” for one study due to insufficient information about the completeness of exposure data (Lee et al., 2009). Three studies were rated as “probably high” because data on exposure level were not shown for all participants (Hughes and Hunting, 2013, Stokholm et al., 2013, Strauss et al., 2014). Two studies was rated as “probably low” because time trend analysis was used to estimate temporal trends for noise exposures within particular standardised job titles (Rabinowitz et al., 2007) (Fig. 2).

##### 4.3.1.5 Bias due to selective reporting of exposures

For the eight included studies, this bias was rated “probably high” for three studies, due to the control group possibly including persons with some previous exposure to occupational exposure to noise, since information on the previous work history of participants was not available; additionally uncontrolled confounders and possible misclassifications due to employment status could have introduced some degree of uncertainty (Hughes and Hunting, 2013, Lee et al., 2009, Strauss et al., 2014). Four studies received a “probably low” rating because the noise was not directly measured, there was indirect evidence that exposure measurement methods were accurate or dosimetry was performed in a small group and broken down for all study participants (Rabinowitz et al., 2007, Stokholm et al., 2013, Virkkunen et al., 2005) (Fig. 2).

##### 4.3.1.6 Bias due to conflict of interest

For the eight included studies, this bias was rated “high” for one study due to two researchers providing consultant services/work to industry (Rabinowitz et al., 2007). One study was rated as “probably high” because the research was funded by grant from industry (Cantley et al., 2015). Two studies were rated as “probably low” because there was indirect evidence which suggested the study was free of support from a company, study author or other entity having a financial interest in the outcome of the study (Stokholm et al., 2013, Virkkunen et al., 2005) (Fig. 2).

##### 4.3.1.7 Bias due to differences in numerator and denominator

For the eight included studies, this bias was rated “probably high” for two studies due to insufficient information provided in study records and our requests for missing data about exposure and control group (including unclear total numbers of study participants for some included studies) were unanswered; it is unclear whether the authors studied all workers (Strauss et al., 2014);. The risk of bias was rated as “probably low” for two studies that dropped some participants from the analysis without clarifying if these were dropped from the numerator and/or denominator (Stokholm et al., 2013, Virkkunen et al., 2005) (Fig. 2).

##### 4.3.1.8 Other bias

For the eight included studies, this bias was rated “probably high” for three studies; for one this due to the declaration that the inclusion of white-collar analyses would impact the results (Virkkunen et al., 2005). Stokholm et al. (2013) declared that individual information on the use of hearing protection devices was not available, which possibly reduced the power to detect an effect. Rabinowitz et al. (2007) did not consider the combined effects of noise (Fig. 2).

#### 4.3.2 Cross-sectional studies

**Fig. 3:**
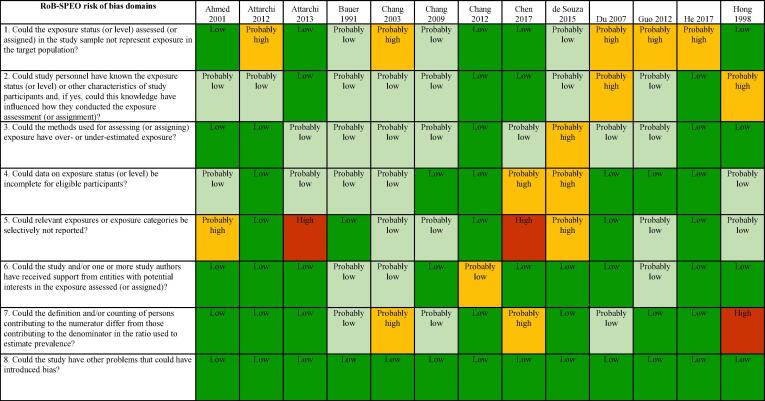

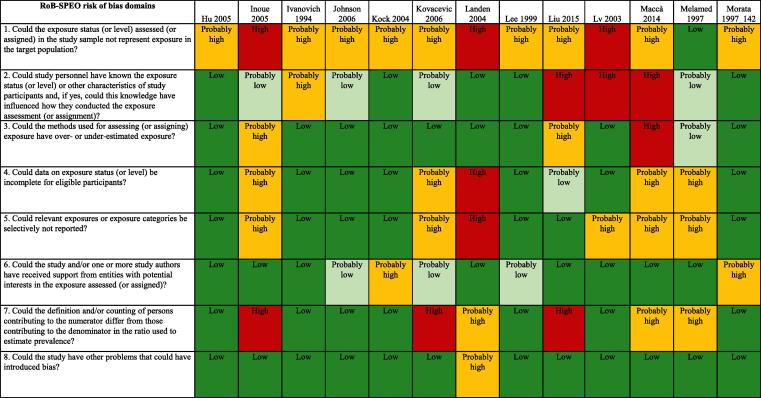

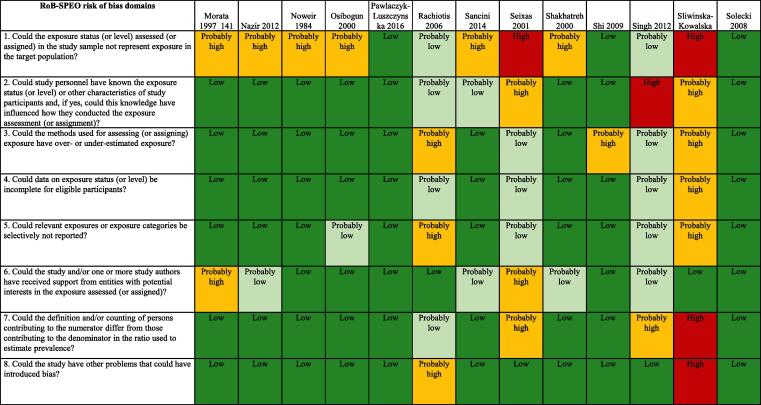

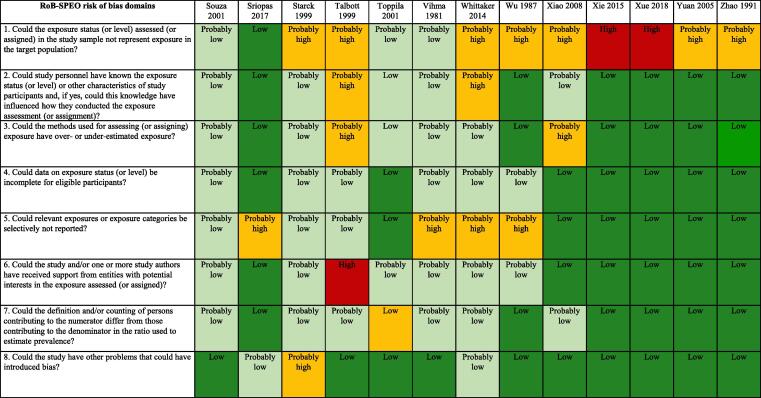
Summary of risk of bias, cross-sectional studies.

Fig. 3 presents an overview of risk of bias in included cross-sectional studies rated along RoB-SPEO’s eight risk of bias domains (Pega et al., 2020b).

##### 4.3.2.1 Bias in selection of participants into the study

We assessed risk of bias in this domain based on whether the groups being compared were the same in all relevant ways (or as close to this as possible) apart from the exposure. Of the 52 included studies, the risk of selection bias was rated to be high for seven studies due to the study records not providing sufficient descriptions of the target population, inclusion/exclusion criteria, recruitment and enrolment procedures, participation/response rates, and/or data on the distribution of relevant study sample and population characteristics (Inoue et al., 2005, Landen et al., 2004, Seixas et al., 2001, Sliwinska-Kowalska et al., 2004, Xie et al., 2015, Xue et al., 2018). Twenty six studies were rated as “probably high” because the recruitment and/or enrolment procedures were not representative of the target population (Attarchi et al., 2012, Chang et al., 2003, Du et al., 2007, Guo et al., 2012, He et al., 2017, Hu et al., 2005, Ivanovich et al., 1994, Johnson et al., 2006, Kock et al., 2004, Kovacevic and Belojevic, 2006, Lee, 1999, Liu et al., 2015, Macca et al., 2015, Morata et al., 1997a, Morata et al., 1997b, Nasir and Rampal, 2012, Noweir, 1984, Osibogun et al., 2000, Sancini et al., 2014, Shakhatreh et al., 2000, Starck et al., 1999, Talbott et al., 1999, Whittaker et al., 2014, Wu et al., 1987, Xiao et al., 2008, Yuan et al., 2005). Nine studies were rated as “probably low” for this item, in general, because indirect evidence suggested that inclusion/exclusion criteria, recruitment and enrolment procedures, and participation/response rates were similar across groups (Bauer et al., 1991, Chang and Chang, 2009, de Souza et al., 2015, Rachiotis et al., 2006, Singh et al., 2012, Souza et al., 2001, Toppila et al., 2001, Vihma, 1981, Zhao et al., 1991). Finally, ten articles were rated as “low” risk of bias for this domain because, in general, the descriptions of the target population, inclusion/exclusion criteria, recruitment and enrolment procedures, participation/response rates were adequately detailed (Ahmed et al., 2001, Attarchi et al., 2013, Chang et al., 2012, Chen et al., 2017, Hong et al., 1998, Melamed et al., 1997, Pawlaczyk-Luszczynska et al., 2016, Shi, 2009, Solecki, 2008, Sriopas et al., 2017) (Fig. 3).

##### 4.3.2.2 Performance bias

From the 52 cross-sectional included studies, the performance bias was rated “high” for four studies, due to exposure assessors and study personnel were not blinded or were incompletely blinded, and the exposure assessment was likely to be influenced by the lack of blinding(Liu et al., 2015, Macca et al., 2015, Singh et al., 2012). Seven studies received a “probably high” rating because indirect evidence suggested that the exposure assessors and study personnel were not adequately blinded (Du et al., 2007, Hong et al., 1998, Ivanovich et al., 1994, Seixas et al., 2001, Sliwinska-Kowalska et al., 2004, Talbott et al., 1999, Whittaker et al., 2014). Eighteen studies were rated as “probably low” because, although the information on blinding was insufficient to permit a rating of “low” risk of bias, indirect evidence indicated that the exposure assessors and study personnel were adequately blinded (Ahmed et al., 2001, Attarchi et al., 2013, Bauer et al., 1991, Chang and Chang, 2009, Chang et al., 2003, de Souza et al., 2015, Guo et al., 2012, Inoue et al., 2005, Johnson et al., 2006, Kovacevic and Belojevic, 2006, Melamed et al., 1997, Rachiotis et al., 2006, Sancini et al., 2014, Souza et al., 2001, Starck et al., 1999, Vihma, 1981, Xiao et al., 2008, Zhao et al., 1991). Lastly, the remaining twenty three studies were rated as “low” because exposure assessors and study personnel were blinded to relevant participant characteristics (e.g. occupation and/or disease status), and the blinding was probably not broken (Attarchi et al., 2012, Chang et al., 2012, Chen et al., 2017, He et al., 2017, Hu et al., 2005, Kock et al., 2004, Landen et al., 2004, Lee, 1999, Morata et al., 1997a, Morata et al., 1997b, Nasir and Rampal, 2012, Noweir, 1984, Osibogun et al., 2000, Pawlaczyk-Luszczynska et al., 2016, Shakhatreh et al., 2000, Shi, 2009, Solecki, 2008, Sriopas et al., 2017, Toppila et al., 2001, Wu et al., 1987, Xie et al., 2015, Xue et al., 2018, Yuan et al., 2005) (Fig. 3).

##### 4.3.2.3 Bias due to exposure misclassification

For the 52 included cross-sectional studies, this bias was rated “high” for only one study (Macca et al., 2015) because it was unclear how exposure assessment was performed and exposure measures were obtained, and there was evidence for concern about the exposure measurement method used. Eight studies were rated as “probably high” because, in general, there was insufficient information about the exposure measurement methods to permit a rating of “high” risk of bias but there was indirect evidence that suggested that the methods were not robust, as described by the criteria for a rating of high risk of bias (de Souza et al., 2015, Inoue et al., 2005, Liu et al., 2015, Rachiotis et al., 2006, Shi, 2009, Sliwinska-Kowalska et al., 2004, Talbott et al., 1999, Xiao et al., 2008). Fifteen studies were rated as “probably low” because there was insufficient information about the exposure measurement methods to permit a rating of “low” risk of bias. However, there was indirect evidence that exposure measurement methods were accurate, as described by the criteria for a rating of “low” risk of bias (Attarchi et al., 2013, Bauer et al., 1991, Chang and Chang, 2009, Chang et al., 2003, Chen et al., 2017, Du et al., 2007, Guo et al., 2012, Melamed et al., 1997, Seixas et al., 2001, Singh et al., 2012, Souza et al., 2001, Starck et al., 1999, Vihma, 1981, Whittaker et al., 2014, Zhao et al., 1991). Lastly, twenty eight studies (Ahmed et al., 2001, Attarchi et al., 2012, Chang et al., 2012, He et al., 2017, Hong et al., 1998, Hu et al., 2005, Ivanovich et al., 1994, Johnson et al., 2006, Kock et al., 2004, Kovacevic and Belojevic, 2006, Landen et al., 2004, Lee, 1999, Lv et al., 2003, Morata et al., 1997a, Morata et al., 1997b, Nasir and Rampal, 2012, Noweir, 1984, Osibogun et al., 2000, Pawlaczyk-Luszczynska et al., 2016, Sancini et al., 2014, Shakhatreh et al., 2000, Solecki, 2008, Sriopas et al., 2017, Toppila et al., 2001, Wu et al., 1987, Xie et al., 2015, Xue et al., 2018, Yuan et al., 2005) were classified as low risk of bias because, in general, the reviewers considered direct evidence that exposure was consistently assessed, using well-established methods that directly measure exposure (Fig. 3).

##### 4.3.2.4 Bias due to incomplete exposure data

For the 52 cross-sectional included studies, this bias was rated high for one study because participation in the study was low enough to possibly introduce bias (Landen et al., 2004). Seven studies were rated as “probably high” because data on exposure level are not shown for all participants (Chen et al., 2017, de Souza et al., 2015, Inoue et al., 2005, Kovacevic and Belojevic, 2006, Macca et al., 2015, Melamed et al., 1997, Sliwinska-Kowalska et al., 2004). Sixteen studies were rated as “probably low” because, in general, the proportion of study participants who participated in the exposure assessment was acceptable and the reasons for non-participation in exposure assessment were acceptable (Ahmed et al., 2001, Attarchi et al., 2013, Bauer et al., 1991, Chang et al., 2003, Hong et al., 1998, Liu et al., 2015, Rachiotis et al., 2006, Seixas et al., 2001, Singh et al., 2012, Souza et al., 2001, Starck et al., 1999, Talbott et al., 1999, Vihma, 1981, Whittaker et al., 2014, Wu et al., 1987, Zhao et al., 1991). The remaining 28 studies were rated as “low” for this item because, on the whole, data on exposure status (or level) seemed complete for eligible participants (Attarchi et al., 2012, Chang and Chang, 2009, Chang et al., 2012, Du et al., 2007, Guo et al., 2012, He et al., 2017, Hu et al., 2005, Ivanovich et al., 1994, Johnson et al., 2006, Kock et al., 2004, Lee, 1999, Lv et al., 2003, Morata et al., 1997a, Morata et al., 1997b, Nasir and Rampal, 2012, Noweir, 1984, Osibogun et al., 2000, Pawlaczyk-Luszczynska et al., 2016, Sancini et al., 2014, Shakhatreh et al., 2000, Shi, 2009, Solecki, 2008, Sriopas et al., 2017, Toppila et al., 2001, Xiao et al., 2008, Xie et al., 2015, Xue et al., 2018, Yuan et al., 2005) (Fig. 3).

##### 4.3.2.5 Bias due to selective reporting of exposures

For the 52 included studies, this bias was rated as “high” for three studies, because there was insufficient information about selective exposure reporting to permit a judgment of “low” or “probably low” risk of bias but there was sufficient evidence, which suggests the study was not free of selective exposure reporting (Attarchi et al., 2013, Chen et al., 2017, Landen et al., 2004). There were fourteen studies rated as “probably high” because, in general, there was indirect evidence, which suggested the study was not free of selective exposure reporting (Ahmed et al., 2001, de Souza et al., 2015, Inoue et al., 2005, Kovacevic and Belojevic, 2006, Lv et al., 2003, Macca et al., 2015, Melamed et al., 1997, Rachiotis et al., 2006, Sliwinska-Kowalska et al., 2004, Sriopas et al., 2017, Vihma, 1981, Whittaker et al., 2014, Wu et al., 1987, Zhao et al., 1991). Ten studies were classified as “probably low” for this item because, in general, the exposure assessment was based on individual exposure measurements (Chang and Chang, 2009, Chang et al., 2003, Guo et al., 2012, Hong et al., 1998, Osibogun et al., 2000, Seixas et al., 2001, Singh et al., 2012, Souza et al., 2001, Starck et al., 1999, Talbott et al., 1999). Finally, the remaining 25 studies were considered as “low” risk of bias because of sufficient information about exposure selective reporting (Attarchi et al., 2012, Bauer et al., 1991, Chang et al., 2012, Du et al., 2007, He et al., 2017, Hu et al., 2005, Ivanovich et al., 1994, Johnson et al., 2006, Kock et al., 2004, Lee, 1999, Liu et al., 2015, Morata et al., 1997a, Morata et al., 1997b, Nasir and Rampal, 2012, Noweir, 1984, Pawlaczyk-Luszczynska et al., 2016, Sancini et al., 2014, Shakhatreh et al., 2000, Shi, 2009, Solecki, 2008, Toppila et al., 2001, Xiao et al., 2008, Xie et al., 2015, Xue et al., 2018, Yuan et al., 2005) (Fig. 3).

##### 4.3.2.6 Bias due to conflict of interest

From the 52 cross-sectional included articles, this bias was rated as “high” for only one study because the authors received funding from a company (ford.com) which could represent a potential interest in the study’s results (Talbott et al., 1999). For five studies the bias was rated “probably high” because, in general, there was indirect evidence which suggests the study was not free of support from a company, study author or other entity having a financial interest in the outcome of the study (Chang et al., 2012, Kock et al., 2004, Morata et al., 1997a, Morata et al., 1997b, Seixas et al., 2001). Seventeen studies were classified as “probably low” because, in general, there was insufficient information to permit a rating of low risk of bias but there is indirect evidence which suggests the study was free of support from a company, study author or other entity having a financial interest in the outcome of the study, as described by the criteria for a rating of low risk of bias (Attarchi et al., 2012, Attarchi et al., 2013, Bauer et al., 1991, Chang and Chang, 2009, Chang et al., 2003, Chen et al., 2017, de Souza et al., 2015, Du et al., 2007, Guo et al., 2012, He et al., 2017, Hong et al., 1998, Hu et al., 2005, Inoue et al., 2005, Ivanovich et al., 1994, Johnson et al., 2006, Kovacevic and Belojevic, 2006, Landen et al., 2004, Lee, 1999, Liu et al., 2015, Lv et al., 2003, Macca et al., 2015, Melamed et al., 1997, Nasir and Rampal, 2012, Noweir, 1984, Osibogun et al., 2000, Pawlaczyk-Luszczynska et al., 2016, Rachiotis et al., 2006, Sancini et al., 2014, Shakhatreh et al., 2000, Shi, 2009, Singh et al., 2012, Sliwinska-Kowalska et al., 2004, Solecki, 2008, Souza et al., 2001, Sriopas et al., 2017, Starck et al., 1999, Toppila et al., 2001, Vihma, 1981, Whittaker et al., 2014, Wu et al., 1987, Xiao et al., 2008, Xie et al., 2015, Xue et al., 2018, Yuan et al., 2005, Zhao et al., 1991. Lastly, twenty nine studies were considered as low risk of bias for this item because, in general, there was sufficient evidence which suggests they were free of competing interest between authors and funding entities {Ahmed et al., 2001 #1597) (Fig. 3).

##### 4.3.2.7 Bias due to differences in numerator and denominator

For the 52 included cross-sectional studies, this bias was rated as “high” for five studies (Hong et al., 1998, Inoue et al., 2005, Kovacevic and Belojevic, 2006, Liu et al., 2015, Sliwinska-Kowalska et al., 2004) because, in general, the numerator and denominator are defined and/or counted differently, and/or the shortest prevalence period was inappropriate (e.g. too short to detect exposure). Seven studies were rated as “probably high” for this bias item because, in general, indirect evidence suggests that the numerator and denominator for the prevalence estimate did not seem appropriate (Chang et al., 2003, Chen et al., 2017, Landen et al., 2004, Macca et al., 2015, Melamed et al., 1997, Seixas et al., 2001, Singh et al., 2012). Eleven studies were classified as “probably low” because, in general, the proportion of persons invited to participate in the study who in fact did participate was acceptable (Bauer et al., 1991, Chang and Chang, 2009, Du et al., 2007, Rachiotis et al., 2006, Souza et al., 2001, Starck et al., 1999, Talbott et al., 1999, Vihma, 1981, Whittaker et al., 2014, Xiao et al., 2008, Zhao et al., 1991). Finally, twenty nine studies were rated as “low” because differences in numerator and denominator seem appropriate (Ahmed et al., 2001, Attarchi et al., 2012, Attarchi et al., 2013, Chang et al., 2012, de Souza et al., 2015, Guo et al., 2012, He et al., 2017, Hu et al., 2005, Ivanovich et al., 1994, Johnson et al., 2006, Kock et al., 2004, Lee, 1999, Lv et al., 2003, Morata et al., 1997a, Morata et al., 1997b, Nasir and Rampal, 2012, Noweir, 1984, Osibogun et al., 2000, Pawlaczyk-Luszczynska et al., 2016, Sancini et al., 2014, Shakhatreh et al., 2000, Shi, 2009, Solecki, 2008, Sriopas et al., 2017, Toppila et al., 2001, Wu et al., 1987, Xie et al., 2015, Xue et al., 2018, Yuan et al., 2005) (Fig. 3)

##### 4.3.2.8 Other bias

From the 52 cross-sectional studies, one was rated as “high” (Sliwinska-Kowalska et al., 2004) because the authors did not study the impact of the use of personal protective equipment on hearing function and did not control for occupational co-exposures to ototoxic chemical agents. Three were rated as “probably high” because there was indirect evidence suggesting the study was not free of other potential sources of bias (Landen et al., 2004, Rachiotis et al., 2006, Starck et al., 1999). Two were rated as “probably low” because there was insufficient information to permit a rating of “low” risk of bias but there was indirect evidence, which suggests the study was free of other potential sources of bias (Sriopas et al., 2017, Whittaker et al., 2014). The vast majority of studies (46) were classified as “low” risk of bias for this item because they appeared to be free of other potential sources of bias (Fig. 3).

#### 4.3.3 Population-based cross-sectional survey

**Fig. 4:**
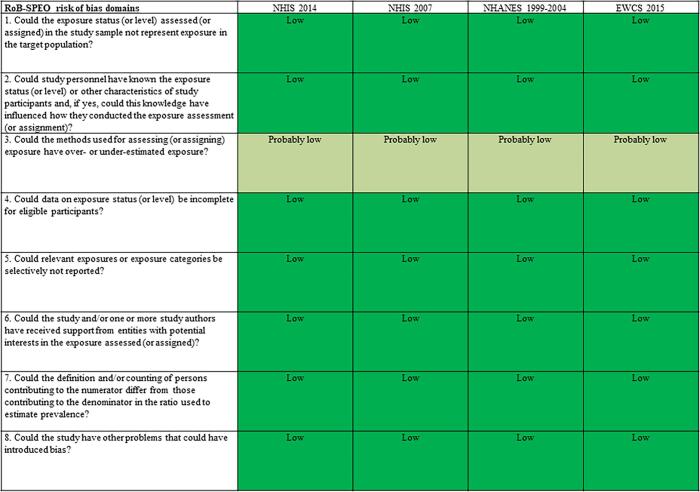
Summary of risk of bias, Population-based cross-sectional surveys.

For the eight domains of risk of bias for the population-based cross-sectional survey, only the bias due to exposure misclassification was rated “probably low” for all studies because the method of assessing occupational noise exposure was subjective (Fig. 4).

### 4.4 Synthesis of results

#### 4.4.1 All prevalence estimates

A data sheet with all prevalence estimates is provided in Appendix 5 in the Supplementary data. If available to us, the prevalence estimates are presented fully disaggregated by country, sex, age group and industrial sector or occupation. The data in the sheet is prepared in a convenient format for ready use as input data for modelling occupational exposure to noise.

#### 4.4.2 Pooled prevalence estimate

**Fig. 5:**
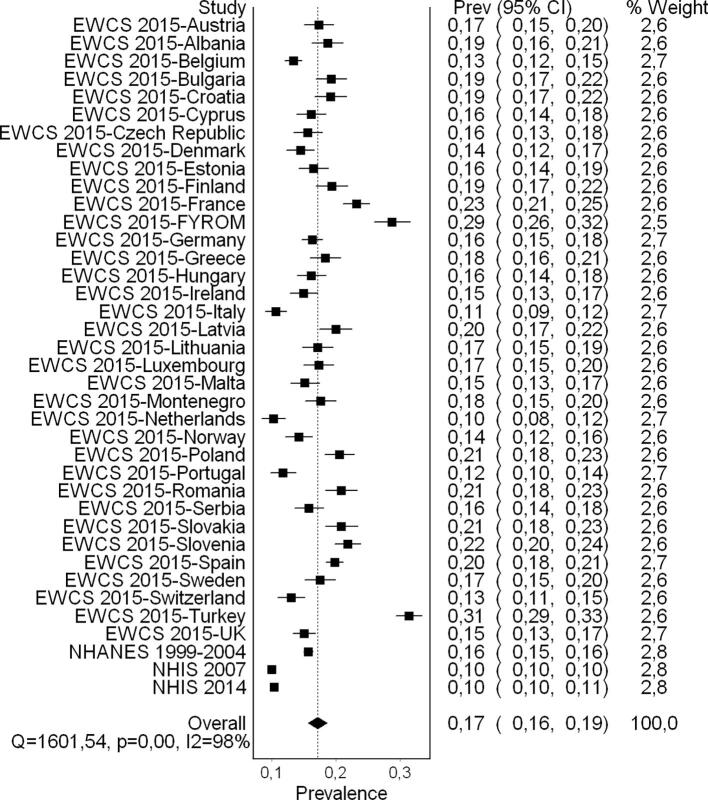
Main meta-analysis, Is occupationally exposed to noise (≥85dBA), studies of general populations of workers.

For our main meta-analysis, we prioritized the included studies that surveyed national probability samples of general populations of workers. These are the European Working Condition Survey (EWCS) (Parent-Thirion et al., 2019); National Health Interview Survey (NHIS) (Center for Disease Control and Prevention, 2007, Center for Disease Control and Prevention, 2014) and National Health and Nutrition Examination Survey (NHANES) (Center for Disease Control and Prevention and National Center for Health Statistics 2020). We judged the other 60 included studies to capture workers in (selected) industrial sectors and/or occupations with relatively high occupational exposure to noise that did not equally represent exposure in the general population of workers, and therefore we excluded these studies from the main meta-analysis. The main meta-analysis therefore included four studies with a total of 108,256 participants from 38 countries in two WHO regions (Americas and Europe) that reported estimates of the prevalence of occupational exposure to noise among workers (general population) using self-reported, subjective measurements collected in large-scale, official surveys. These four studies were thus relatively homogenous in population and exposure. The forest plot from the meta-analysis is presented in Fig. 5. The pooled prevalence of any occupational exposure to noise was 0.17 (95% CI 0.16 to 0.19, 4 studies, 108,256 participants, 38 countries). The statistical heterogeneity was high, with an I^2^ of 98%.

### 4.5 Additional analyses

#### 4.5.1 Subgroup analyses

We conducted subgroup analyses using all studies included in the systematic review, where results were disaggregated by the relevant variable, including the population-based studies.

##### 4.5.1.1 By WHO region and/or country

**Fig. 6:**
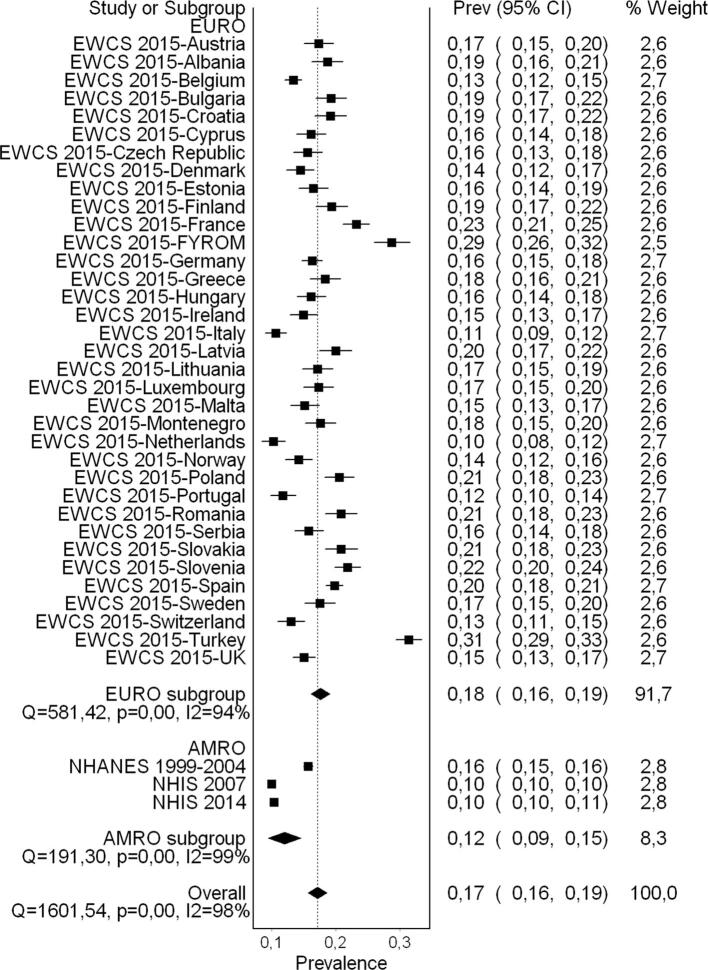
Subgroup analysis by WHO region, Is occupationally exposed to noise (≥85dBA), studies of the general population of workers.

**Fig. 7:**
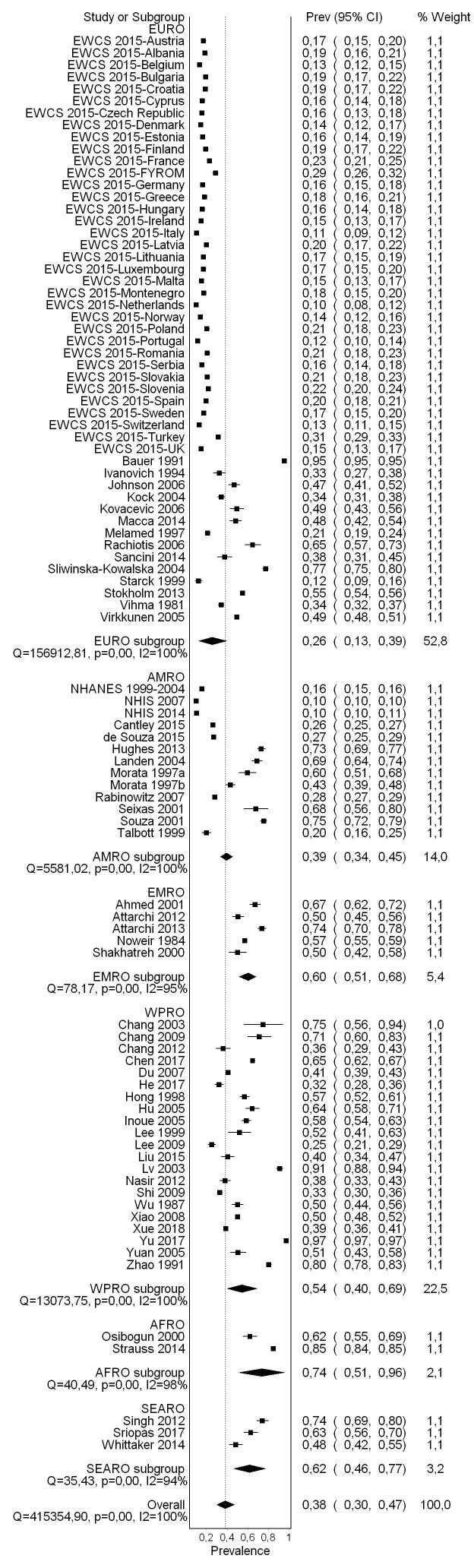
Subgroup analysis by WHO region, Is occupationally exposed to noise (≥85dBA), all included studies.

By WHO region, the pooled prevalence estimates for studies of the general population of workers is presented in Fig. 6 and Table 4 and for studies of studies of workers in selected occupations and/or industrial sectors with higher exposure is presented in Fig. 7 and Table 5. In both analyses, the prevalence estimates differed substantially between the WHO regions included in the respective subgroup analyses (test for subgroup differences p < 0.001).

**Table 4:** Subgroup analysis by WHO region, Is occupationally exposed to noise, studies of the general population of workers.

WHO region	Prevalence (95% confidence interval)	Number of studies^2^
Africa	–	–
Americas	0.12 (0.09–0.15)	3 studies
Eastern Mediterranean	–	–
Europe	0.18 (0.16–0.19)	1 study
South-East Asia	–	–
Western Pacific	–	–

Footnotes: Test for subgroup differences found p < 0.001.

**Table 5:** Subgroup analysis by WHO region, Is occupationally exposed to noise, studies of workers in selected occupations and/or industrial sectors with higher exposure.

WHO region	Prevalence (95% confidence interval)	Number of studies
Africa	0.74 (0.51–0.96)	2 studies
Americas	0.39 (0.34–0.45)	13 studies
Eastern Mediterranean	0.60 (0.51–0.68)	5 studies
Europe	0.26 (0.13–0.39)	15 studies
South-East Asia	0.62 (0.46–0.77)	3 studies
Western Pacific	0.54 (0.40–0.69)	21 studies

Footnotes: Test for subgroup differences found p < 0.001.

##### 4.5.1.2 By sex

**Fig. 8:**
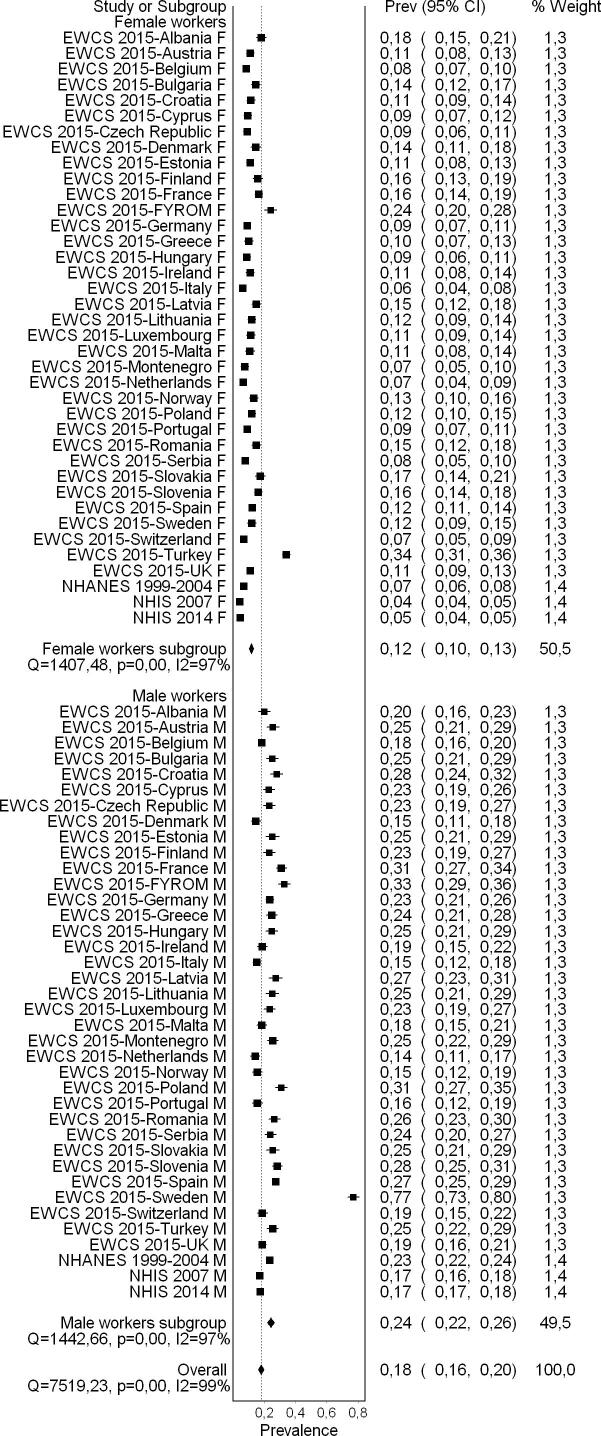
Subgroup analysis by sex, Is occupationally exposed to noise (≥85dBA).

As noted above (see Section 3.9), for the subgroup analyses by sex, we prioritized evidence from the included studies that surveyed national probability samples of general populations of workers. The reason for this was that these studies provided the prevalence that we were interested in. The subgroup analysis (Fig. 8) found that the prevalence of occupational exposure to noise was lower among females with 0.12 (95% CI 0.10 to 0.13) than among males with 0.24 (95% CI 0.21 to 0.26)). The test of subgroup differences was statistically significant (p < 0.001).

##### 4.5.1.3 By industrial sector

**Fig. 9:**
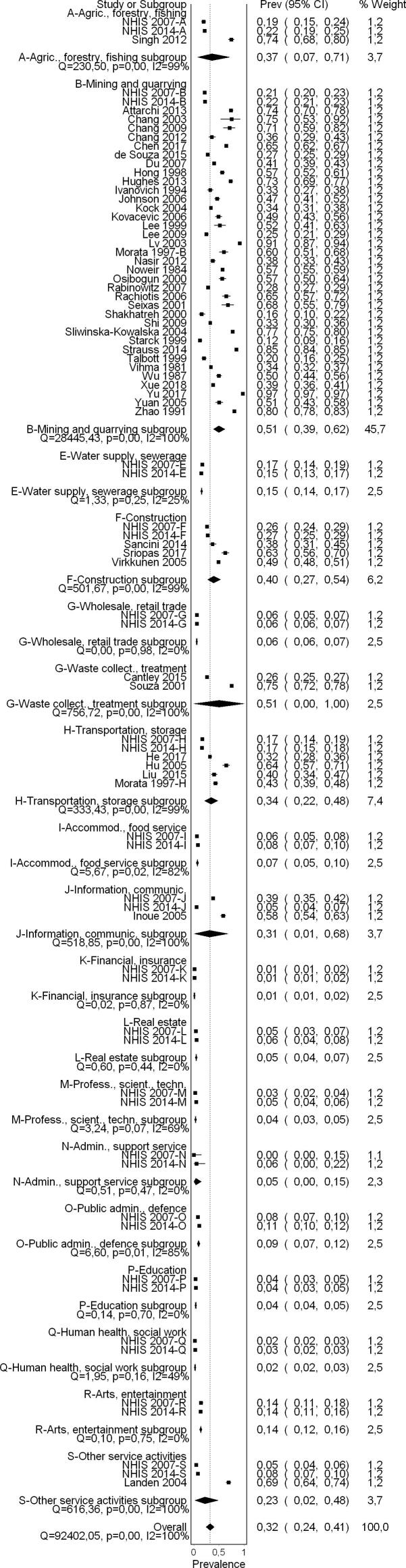
Subgroup analysis by industrial sector, Is occupationally exposed to noise (≥85dBA), studies of general populations of workers.

By industrial sector, the pooled prevalence estimates for ISIC codes at one-digit level with available data are presented in Fig. 9 and Table 6. Prevalence estimates differed substantially between industrial sectors (test for subgroup differences p < 0.001) (see Table 6).

**Table 6:** Subgroup analysis by industrial sector, Is occupationally exposed to noise, studies of general populations of workers.

Industrial sector (ISIC code)	Prevalence (95% confidence interval)	Number of studies
A Agriculture, forestry and fishing	0.37 (0.07–0.17)	3 studies
B Mining and quarrying	0.51 (0.39–0.62)	38 studies
E Water supply; sewerage, waste management and remediation activities	0.15 (0.14–0.17)	2 studies
F Construction	0.40 (0.27–0.54)	5 studies
G Wholesale and retail trade; repair of motor vehicles and motorcycles	0.06 (0.06–0.07)	2 studies
H Transportation and storage	0.34 (0.22–0.48)	6 studies
I Accommodation and food service activities	0.07 (0.05, 0.10)	2 studies
J Information and communication activities	0.31 (0.01–0.68)	3 studies
K Financial and insurance activities	0.01 (0.01–0.02)	2 studies
L Real estate activities	0.05 (0.04–0.07)	2 studies
M Professional, scientific and technical activities	0.04 (0.03–0.05)	2 studies
N Administrative and support service activities	0.05 (0.00–0.15)	2 studies
O Public administration and defence; compulsory social security	0.11 (0.08–0.14)	2 studies
P Education	0.04 (0.04–0.05)	2 studies
Q Human health and social work activities	0.03 (0.02–0.03)	2 studies
S Other service activities	0.23 (0.02–0.48)	3 studies

Footnotes: Test for subgroup differences found p < 0.001.

##### 4.5.1.4 By occupation

**Fig. 10:**
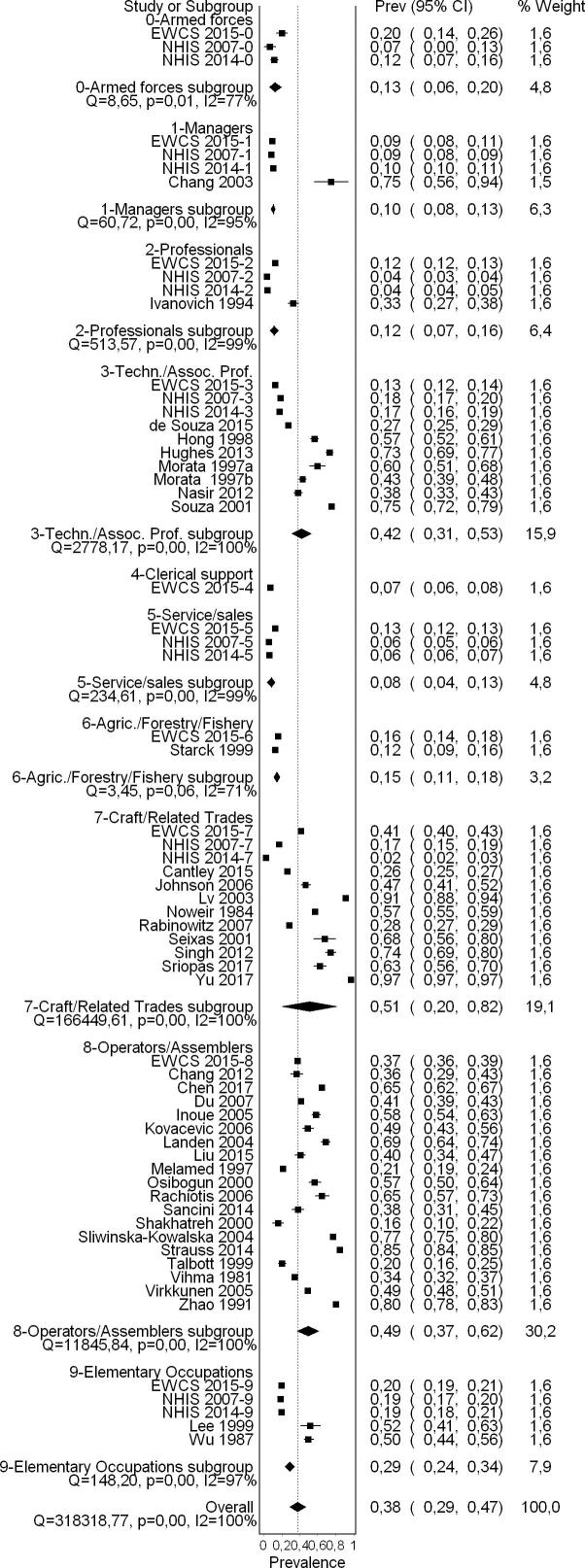
Subgroup analysis by occupation, Is occupationally exposed to noise (≥85dBA).

By occupation, the pooled prevalence for ISCO codes (at one-digit level) with available data are presented in Fig. 10. For studies that did not report ISCO codes, we coded occupations ourselves based on the information reported in the study records or other study documentation that we accessed, such as study protocols and questionnaires. Evidence included within subgroups defined by occupation may be heterogeneous. Different studies may have coded the same occupations differently. Exposure might differ in the same occupation. For example, in the People's Republic of China, large enterprises often use automated machines which decrease the exposure time of noise for workers, whereas small and medium-sized enterprises may not use these more expensive, newer machines, suggesting that occupational exposure to noise differs by enterprise size even within occupation. The forest plot of the subgroup analysis (Fig. 10) shows that prevalence estimates differed substantially between the nine groups of occupations captured in the body of evidence included in this systematic review (test for subgroup differences p < 0.001) (see Table 7).

**Table 7:** Subgroup analysis by occupation, Is occupationally exposed to noise.

Industrial sector (ISIC code)	Prevalence (95% confidence interval)	Number of studies
0 Armed forces occupations	0.13 (0.06–0.20)	3 studies
1 Managers	0.10 (0.08–0.13)	4 studies
2 -Professionals	0.12 (0.07–0.16)	4 studies
3 Technicians and Associate Professionals	0.42 (0.31–0.53)	10 studies
4 Clerical support workers	0.07 (0.06–0.08)	1 study
5 Service and sales workers	0.08 (0.04–0.13)	3 studies
6 Skilled Agricultural, Forestry and Fishery Workers	0.15 (0.11–0.18)	2 studies
7 Craft and Related Trades Workers	0.51 (0.20–0.82)	12 studies
8 Plant and Machine Operators and Assemblers	0.49 (0.37–0.62)	19 studies
9 Elementary Occupations	0.29 (0.24–0.34)	5 studies

Footnotes: Test for subgroup differences found p < 0.001.

### 4.6 Quality of evidence

Using the QoE-SPEO approach for assessing quality of evidence of the entire body of evidence, which WHO developed specifically for the WHO/ILO Joint Estimates (forthcoming), we judged the body of evidence used in the main meta-analysis. This is the prioritized evidence from the four population-based studies of the general population of workers.

#### 4.6.1 Expected heterogeneity

The expected heterogeneity is defined as “the actual, real and non-spurious variability in the prevalence of exposure within or between individual workers” (Pega et al., submitted for publication). We anticipated heterogeneity both within and between workers, based on different levels of occupational exposure to noise between and within occupations, work sites, regulations and compliance, use of personal protective equipment, job tasks, and technology and tools used. Consequently, we rated expected heterogeneity as “high”.

#### 4.6.2 Risk of bias

The use of survey questions may have introduced serious risk of bias across studies in the third RoB-SPEO domain (Pega et al., 2020a, Pega et al., 2020b): misclassification bias in the assessment of the exposure to the risk factor. Such bias could have occurred even though the survey questions that are used in the included studies are standardized and validated against objective, individual measurements. We downgraded the quality of evidence by one level (−1) due to our serious concerns in this QoE-SPEO domain.

#### 4.6.3 Indirectness

Evidence is limited to populations in 38 countries in two WHO regions. All of these countries are high-income countries. We have serious concerns that this study population does not match the population of interest. We downgraded the quality of evidence for “serious” concerns for indirectness by one level (−1).

#### 4.6.4 Inconsistency, imprecision, and publication bias

We did not have any serious concerns in the domains of inconsistency, imprecision or publication bias. We had rated expected heterogeneity as “high” and found high statistical variability across studies (I^2^ 98%), and therefore we were not concerned for inconsistency. Although expected heterogeneity was “high”, the 95% CI of the pooled prevalence estimate is narrow, but the pooled body of evidence includes a large sample (108,256 participants), and this probably explains the relatively narrow CI even if heterogeneity is expected to be high between and within studies. No evidence was found for publication bias, and the body of evidence consists of established, large studies conducted by producers of official statistics. Therefore, we did not downgrade for any of these three QoE-SPEO domains (+/- 0).

#### 4.6.5 Final decision on quality of evidence

The grading started from a rating of “high”. We downgraded the quality of evidence by one grade each for risk of bias and indirectness. Our final rating was therefore that the body of evidence was of “low quality”; further research is very likely to have an important impact on our confidence in the estimate of prevalence and is likely to change the estimate.

## 5 Discussion

### 5.1 Summary of evidence

As shown in the table of summary of findings (Table 8), the pooled prevalence of any occupational exposure to noise was 0.17 (95% CI 0.16 to 0.19, 4 studies, 108,256 participants, 38 countries, I^2^ 98%). By region, the pooled prevalence was 0.74 for Africa (95% CI 0.51 to 0.96, 2 studies); 0.39 for the Americas (95% CI 0.34 to 0.45, 13 studies); 0.60 for the Eastern Mediterranean (95% CI 0.51 to 0.68, 5 studies); 0.26 for Europe (95% CI 0.13 to 0.39, 15 studies); for South-East Asia was 0.62 (95% CI 0.46 to 0.77, 3 studies); and 0.54 for the Western Pacific (95% CI 0.40 to 0.69, 21 studies) (test for subgroup differences p < 0.001). Subgroup analyses showed that pooled prevalence also differed by sex, industrial sector and occupation.

### 5.2 Comparison with previous systematic reviews evidence

To our knowledge, there is no prior systematic review or meta-analytic evidence that we could compare our systematic review and meta-analysis against.

**Table 8:** Summary of findings.

**Prevalence of any occupational exposure to noise**				
**Population:** Any workers **Settings:** All countries and work settings **Exposure:** Occupational exposure to noise (>85dBA)				
**Outcome**	**Prevalence estimate (95% CI)**	**No. of participants (studies)**	**QoE-SPEO quality of the evidence rating**	**Comments**
Any occupational exposure to noise (defined as ≥ 85 dBA; measured using indirect (survey) measurements)	**0.17** (0.16, 0.19)	108,256 participants (4 studies)	⊕⊕⊝⊝ ^a,b,^^c^ Low	The other 60 studies not included in the main meta-analysis generally support this estimate but indicated higher prevalence because they studied non-general populations at higher risk (defined by occupations and industrial sectors with known high occupational exposure to noise).
**CI**: confidence interval				
QoE-SPEO quality of evidence ratings: **High quality:** Further research is very unlikely to change our confidence in the estimate of prevalence. **Moderate quality:** Further research is likely to have an important impact on our confidence in the estimate of prevalence and may change the estimate. **Low quality:** Further research is very likely to have an important impact on our confidence in the estimate of prevalence and is likely to change the estimate. **Very low quality:** We are very uncertain about the estimate.				

^a^Expected heterogeneity was rated as “high”.

^b^Downgraded by one grade because of serious concern for risk of bias (primarily for bias in the exposure assessment).

^c^Downgraded by one grade because of serious concern for indirectness.

## 6 Use of evidence for burden of disease estimation

This systematic review and meta-analysis was conducted by WHO and ILO, supported by a large network of experts, for the development of the WHO/ILO Joint Estimates. More specifically, it provides the crucial evidence base for both organizations to consider producing global health estimates of occupational exposure to noise. The systematic review found a large body of evidence comprising many studies. Overall, we judged this body of evidence to be of low quality (at least the prioritized evidence that contributed the main analysis). Producing estimates of occupational exposure to noise appears to be evidence-based and warranted, and the pooled prevalence estimate from the main and subgroup analyses appear suitable as input data for WHO/ILO Joint Estimates.

## 7 Conclusions

Our systematic review and meta-analysis found that occupational exposure to noise is prevalent among general populations of workers. The current body of evidence is, however, of low quality, due to serious concerns for risk of bias and indirectness. Producing estimates occupational exposure to noise nevertheless appears evidence-based, and the pooled effect estimates presented in this systematic review are suitable as input data for the WHO/ILO Joint Estimates.

## 8 Differences between protocol and systematic review

● We intended to use a modified version of the Navigation Guide risk of bias tool, but then developed a tool specifically for assessing risk of bias in studies estimating prevalence of exposure with occupational exposure to noises (RoB-SPEO; Pega et al., 2020b) and applied this dedicated tool in our systematic review.
● We intended to use a modified version of the Navigation Guide approach for assessing quality of evidence, but then WHO, with a Working Group of individual experts, developed the QoE-SPEO approach specifically for assessing quality of evidence in studies estimating prevalence of exposure to occupational risk factors (Pega et al., submitted for publication) and then applied this dedicated tool in our systematic review.
● In our protocol we planned to conduct sensitivity analyses, but we did not conduct these.
● We did not intend to quantitatively meta-analyse prevalence estimates but did so in the systematic review because the health estimation will require pooled estimates, rather than individual estimates only.

## Financial support

All authors are salaried staff members of their respective institutions. The publication was prepared with financial support from the World Health Organization cooperative agreement with the Centres for Disease Control and Prevention National Institute for Occupational Safety and Health of the United States of America (Grant 1E11OH0010676-02; Grant 6NE11OH010461-02-01; and Grant 5NE11OH010461-03-00); the German Federal Ministry of Health (BMG Germany) under the BMG-WHO Collaboration Programme 2020-2023 (WHO specified award ref. 70672); and the Spanish Agency for International Cooperation (AECID) (WHO specified award ref. 71208).

## Sponsors

The sponsors of this systematic review are the World Health Organization and the International Labour Organization.

## Author contributions

Had the idea for this systematic review: FP, Ivan Ivanov (WHO), Nancy Leppink (ILO).

Selected the lead reviewers and gathered the review teams: FP, Ivan Ivanov, Nancy Leppink.

Coordinated the entire series of systematic reviews: FP, Yuka Ujita (ILO).

Were the lead reviewers of this systematic review: LT, DS.

Led the design of the systematic review including developed the standard methods: FP.

Contributed substantially to the design of the systematic review: LT, TA, WA, MA, MM, DS.

Conducted the search: LT, FP, TA, WA, MA, MM, WH, XS, MZ, DS.

Selected studies: LT, TA, WA, MA, MM, WH, XS, MZ, DS.

Extracted data: LT, TA, WA, MA, MM, WH, XS, MZ, DS, AMD.

Requested missing data: LT, DS, AMD.

Assessed risk of bias: LT, TA, MA, MM, WH, XS, MZ, DS.

Conducted the meta-analyses: LT, FP, CA, AMD, DS.

Assessed quality of evidence: LT, FP, AMD, DS.

Developed the standards and wrote the template for all systematic reviews in the series: FP.

Wrote the first draft of the manuscript using the template: LT, FP, AMD, DS.

Revised the manuscript critically for important intellectual content: LT, FP, AMD, TA, WA, MA, MM, DS.

Ensured tailoring of the systematic review for WHO/ILO estimation purposes: FP.

Ensured harmonization across systematic reviews in the series: FP.

Approved the final version of the systematic review to be published: All authors.

Agreed to be accountable for all aspects of the work in ensuring that questions related to the accuracy or integrity of any part of the work are appropriately investigated and resolved: All authors.

## Declaration of Competing Interest

The authors declare that they have no known competing financial interests or personal relationships that could have appeared to influence the work reported in this paper.
