# Supplementary material for: The prevalence of occupational exposure to noise: A systematic review and meta-analysis from the WHO/ILO Joint Estimates of the Work-related Burden of Disease and Injury
Source: Environ Int. 2021 Sep;154:106380. doi: 10.1016/j.envint.2021.106380 (PMC8204275; doi:10.1016/j.envint.2021.106380)
Supplement: Supplementary data 1 [file mmc1.docx]

**Appendix 1 Search strategies for electronic academic databases**

| DATABASE/Date | STRATEGIES |
| --- | --- |
| Pubmed  April 26^st^, 2018 | 1. Noise, Occupational[MESH] OR ((noise*[TIAB] OR noisy[TIAB] OR sound[TIAB] OR loud[TIAB]) AND (occupation*[TIAB] OR work*[TIAB] OR employ*[TIAB] OR labour*[TIAB] OR labor*[TIAB] OR job[TIAB])) 2. "Epidemiologic Studies"[Mesh] OR "Clinical Trial" [Publication Type] OR "Observational Study" [Publication Type] OR "Comparative Study" [Publication Type] OR Trial[TW] OR random*[TW] OR experiment*[TW] OR ((intervention[TW] OR observational[TW] OR epidemiologic*[TW] OR panel*[TW] OR “follow up”[TIAB] OR exposure*[TW]) AND (study[TIAB] OR studies[TIAB] OR analys*[TIAB])) OR Cohort*[TW] OR longitudinal*[TW] OR retrospective*[TW] OR prospective*[TW] OR "time series"[TIAB] OR before-after[TIAB] 3. Seroepidemiologic Studies[MESH] OR Cross-Sectional Studies[MESH] OR “cross sectional”[TIAB] 4. #1 and (#2 not #3) |
| Medline/Ovid  April 26^st^, 2018 | 1. Noise, Occupational/ 2. ((noise* or noisy or loud or sound*) and (occupation* or work* or employ* or labour* or labor* or job)).mp. 3. 1 or 2 4. exp Epidemiologic Studies/ or Clinical Trial/ or Observational Study/ or Comparative Study/ 5. (Trial or experiment*).tw. 6. ((intervention or observational or epidemiologic* or panel* or 'follow up' or exposure) adj (study or studies or analys* or data)).tw. 7. (longitudinal* or retrospective* or prospective*).tw. 8. (random* or 'time series' or before-after).mp. [mp=title, abstract, original title, name of substance word, subject heading word, floating sub-heading word, keyword heading word, organism supplementary concept word, protocol supplementary concept word, rare disease supplementary concept word, unique identifier, synonyms] 9. 4 or 5 or 6 or 7 or 8 10. exp Seroepidemiologic Studies/ or Cross-Sectional Studies/ or 'cross sectional'.tw. 11. 9 or 10 12. 3 and 11 |
| Embase  April 26^th^ 2018 | - 1. ('occupational health'/exp AND 'noise'/exp) OR 'industrial noise'/exp/mj or 'noise pollution'/exp)   2. 'noise*':ti,ab,kw OR 'noisy':ti,ab,kw OR 'loud':ti,ab,kw   3. 'occupation*':ti,ab,kw OR 'work*':ti,ab,kw OR 'employ*':ti,ab,kw OR 'labour*':ti,ab,kw OR 'labor*':ti,ab,kw OR 'job':ti,ab,kw   4. 2 AND 3   5. 1 OR 4   6. 5 AND   7. (filter Study types) ('clinical article'/de OR 'clinical trial'/de OR 'cohort analysis'/de OR 'comparative study'/de OR 'controlled study'/de OR 'human experiment'/de OR 'major clinical study'/de OR 'prospective study'/de OR 'randomized controlled trial'/de OR 'retrospective study'/de)   8. 13. AND [embase]/lim NOT ([embase]/lim AND [medline]/lim) |
| Web of Science  May 03^th^ 2018 | 1. TS=(“Occupational health” and “noise”) OR TS=(“industrial noise” or “noise pollution") 2. TS=(noise* OR noisy OR loud) 3. TS=(occupation* OR work* OR employ* OR labour* OR labor* OR job) 4. 2 and 3 5. 1 or 4 6. TS=(“clinical article” OR “clinical trial” OR cohort OR “comparative study” OR “controlled study” OR “human experiment” OR “clinical study” OR prospective OR “randomized controlled trial” OR retrospective OR random* OR case-control OR non-randomized OR quasi-randomized OR before-after OR “BEFORE AND AFTER” OR “time series”) 7. 5 AND 6 |
| Scopus  April 26^th^ 2018 | 1. ( TITLE-ABS-KEY ( ( "Occupational health" AND "noise" ) ) OR TITLE-ABS-KEY ( ( "industrial noise" OR "noise pollution" ) ) ) = 22.211 2. TITLE-ABS-KEY ( noise* OR noisy ) W/8 TITLE-ABS-KEY ( occupation* OR work* OR employ* OR labour* OR labor* OR job ) = 37,441 3. 1 or 2 = 54.209 4. ( TITLE-ABS-KEY ( "clinical article" OR "clinical trial" OR cohort OR "comparative study" OR "controlled study" OR "human experiment" OR "clinical study" OR prospective OR "randomized controlled trial" OR retrospective ) OR TITLE-ABS-KEY ( random* OR case-control OR non-randomized OR quasi-randomized OR before-after OR "BEFORE AND AFTER" OR "time series" ) ) 5. 3 and 4 |
| Lilacs  April 26^th^ 2018 | ((mh:("Ruido Ocupacional")) OR (mh:("Saúde do Trabalhador")) AND (tw:(ruido OR barulho OR barullo OR noise OR noisy OR sound OR sonido OR som)) OR ((tw:("Occupational health" OR “saúde ocupacional” OR “saúde do trabalhador” OR “salud ocupacional” OR “salud del trabajador” OR “salud laboral”)) AND (tw:(ruido OR barulho OR barullo OR noise OR noisy OR sound OR sonido OR som))) OR ((tw:(ruido OR barulho OR barullo OR noise OR noisy OR sound OR sonido OR som)) AND (tw:(occupation* OR work* OR employ* OR labour* OR labor* OR job)))) |

**Appendix 2 Data extraction notes on NHIS 2014, NHIS 2007, NHANES 1999-2004 and EWCS 2015**

The Centers for Disease Control and Prevention (CDC) of the US grants researchers academic access to primary data from its surveys through public-use data files for statistical analysis or reporting purposes. All information used in the present study was obtained anonymized from the website of the CDC.

The **National Health Interview Survey (NHIS)** is a population-based cross-sectional survey conducted in the US by the National Center for Health Statistics (NCHS) (https://www.cdc.gov/nchs/nhis/index.htm). All collected data are based on a questionnaire administered during a personal household interview. We used the 2014 and 2007 Data Release files.

The **National Health and Nutrition Examination Survey (NHANES)** is also an ongoing series of cross-sectional surveys repeated every two years in the US and conducted by the NCHS (https://www.cdc.gov/nchs/nhanes/index.htm). We used combined data from three consecutive waves of the NHANES (1999-2000, 2001-2002, and 2003-2004). Those were selected because they used the same definition of self-reported noise exposure. The three waves were merged into one 1999-2004 NHANES dataset in order to increase the sample size and allow disaggregation of prevalence by sex and age group.

In NHIS 2014, participants were considered exposed to loud/very loud occupational exposure to noise (a proxy for ≥ 85/90dBA) if they answered affirmatively to either or both of the questions “Have you ever had a job, or combination of jobs, where you were exposed to VERY LOUD sounds or noise for 4 OR MORE HOURS A DAY, SEVERAL DAYS A WEEK?” and “Have you ever had a job, or combination of jobs, where you were exposed to LOUD sounds or noise for 4 OR MORE HOURS A DAY, SEVERAL DAYS A WEEK?”. They also had to have confirmed that their exposure occurred either during the last 12 months or both during and before the last 12 months by answering the questions: “When were you exposed to VERY LOUD sounds or noise at work… during the last 12 months, before then, or both during and before the last 12 months?” and “When were you exposed to LOUD sounds or noise at work… during the last 12 months, before then, or both during and before the last 12 months?”.

In NHIS 2007, participants were considered exposed if they answered affirmatively to the question “Have you ever had a job, or combination of jobs, where you were exposed to loud sounds or noise for 4 or more hours a day, several days a week? Loud means so loud that you must speak in a raised voice to be heard”. Then they had confirmed that their exposure occurred during the last 12 months by answering the question “Was any of this exposure to loud sounds or noise IN THE PAST 12 MONTHS?”.

In NHANES 1999-2004, participants were considered exposed if they answered affirmatively to the question “Thinking of all the jobs you have ever had, have you ever been exposed to loud noise at work for at least three months? By loud noise I mean noise so loud that you had to speak in a raised voice to be heard?” and then confirmed that they were currently exposed at their job (“At [your/SP's] job as a(n) [OCCUPATION] for [EMPLOYER], [are you/is s/he] currently exposed to loud noise?”).

The Eurofound datasets are stored with the UK Data Service (UKDS) in Essex, UK. The **European Working Conditions Surveys** (EWCS) is a pan-European cross-sectional representative questionnaire-based survey conducted every 5 years by the European Foundation for the Improvement of Living and Working Conditions (<https://www.eurofound.europa.eu/surveys/european-working-conditions-surveys-ewcs>). It covers the active population aged ≥ 15 years. Information on occupational exposure to noise was collected through face-to-face interviews at participants’ home. We used the latest dataset available from the 6^th^ EWCS in 2015 (<https://www.eurofound.europa.eu/surveys/european-working-conditions-surveys/sixth-european-working-conditions-survey-2015>).

In EWCS 2015, participants were considered exposed to loud occupational exposure to noise (a proxy for ≥ 85dBA) if they answered affirmatively to the question about their main paid job: “Please tell me, using the following scale, are you exposed at work to ... Noise so loud that you would have to raise your voice to talk to people?” for at least “Around half of the time”.

**Appendix 3 Selected excluded studies and reason for their exclusion**

| **Study (Study ID)** | **Reason for exclusion** |
| --- | --- |
| Aguilar 1990 | Ineligible study type |
| Alizadeh 2016 | Ineligible population studied |
| Alleyne 1985 | Ineligible exposure studied |
| Almeida 2007 | Ineligible exposure studied |
| Alterman 2008 | Ineligible exposure studied |
| Alves 2012 | Ineligible study type |
| Amaral 2014 | Ineligible study type |
| Amirov 1992 | Ineligible study type |
| Anon 1988 | Ineligible exposure studied |
| Anon 1984 | Ineligible study type |
| Anon 1981 | Ineligible study type |
| Anttonen 1994 | Ineligible exposure studied |
| Ballesteros 2012 | Ineligible population studied |
| Barone 1987 | Ineligible exposure studied |
| Bartosi 2002 | Ineligible study type |
| Benavides 1997 | Ineligible exposure studied |
| Bhatt 2016 | Ineligible exposure studied |
| Blanc 1992 | Ineligible study type |
| Bogadi-Šare 2009 | Ineligible exposure studied |
| Borchgrevink 2005 | Ineligible exposure studied |
| Brahams 1984 | Ineligible study type |
| Burr 2005 | Ineligible exposure studied |
| Caciari 2013 | Ineligible population studied |
| Cambou 2003 | Ineligible exposure studied |
| Campos-Serna 2013 | Ineligible study type |
| Canosa 1995 | Ineligible study type |
| Cantley 2015 | Ineligible population studied |
| Cardoso 1992 | Ineligible study type |
| Cediel 2014 | Ineligible study type |
| Centers for Disease Control and Prevention 1988 | Ineligible exposure studied |

**Appendix 4**

Risk of bias table, Ahmed 2001

| **Risk of bias domain** | **Bias in selection of participants into the study (or selection bias)** | **Performance bias** | **Bias due to exposure misclassification** | **Bias due to incomplete exposure data** | **Bias due to selective reporting of exposures** | **Bias due to conflicts of interest** | **Bias due to differences in numerator and denominator** | **Other biases** |
| --- | --- | --- | --- | --- | --- | --- | --- | --- |
| **Rating** | **LOW** | **PROBABLY LOW** | **LOW** | **PROBABLY LOW** | **PROBABLY HIGH** | **LOW** | **LOW** | **LOW** |
| **Justification for rating** | The study records provide sufficient detail for assessing risk of bias, including the: target population; study sample; criteria for eligibility for individuals to participate in the study; sampling, recruitment and enrolment procedures; rate of participation in the study; and rate of participation in the exposure assessment. The data reported in the study records are sufficiently detailed to support the conclusion that exposure in the study sample is representative of exposure in the target population, with similar characteristics reported for both the study sample and the target population. | The information on blinding is insufficient to permit a rating of low risk of bias. However, indirect evidence suggests that the exposure assessors and study personnel were adequately blinded or blinding was unlikely to influence exposure assessment, as described by the criteria for a rating of low risk of bias. | *Indirect* evidence suggests that the method used for assessing and assigning exposure produces valid, reliable and accurate measures of the exposure of interest. For example, a less established or less direct method was used, but there is indirect evidence that it performs equally well as a valid, reliable and accurate method or there is no validated exposure assessment or assignment method. | There is insufficient information about the completeness of exposure data to permit a judgment of low risk of bias, but there is indirect evidence, which suggests incomplete exposure data were adequately addressed, as described by the criteria for a judgment of low risk of bias. | There is insufficient information about selective exposure reporting to permit a judgment of high risk of bias, but there is indirect evidence, which suggests the study was not free of selective exposure reporting, as described by the criteria for a judgment of high risk of bias. It is not mentioned whether the other neighboring industries had the same characteristics as the two factories in of the study. | There is no evidence that the study conduct or reporting were influenced by a competing interest. | I judge the study to be free of other potential sources of bias. | I judge the study to be free of other potential sources of bias. |

Risk of bias table, Attarchi 2012

| **Risk of bias domain** | **Bias in selection of participants into the study (or selection bias)** | **Performance bias** | **Bias due to exposure misclassification** | **Bias due to incomplete exposure data** | **Bias due to selective reporting of exposures** | **Bias due to conflicts of interest** | **Bias due to differences in numerator and denominator** | **Other biases** |
| --- | --- | --- | --- | --- | --- | --- | --- | --- |
| **Rating** | **PROBABLY HIGH** | **PROBABLY LOW** | **LOW** | **LOW** | **LOW** | **LOW** | **LOW** | **LOW** |
| **Justification for rating** | The study's eligibility criteria systematically excluded participants in the study sample such that exposure in the study sample may not represent exposure in the target population. | The exposure assessment was based on the environmental noise assessment and study personnel were adequately blinded or blinding was unlikely to influence exposure assessment, as described by the criteria for a rating of low risk of bias. | Direct evidence suggests that the method adopted in the study for the exposure assessment and assignment produces valid, accurate and reliable exposure data, ideally based on direct quantitative exposure measures. | There were no or minimal incomplete exposure data, or any incomplete exposure data have been imputed using appropriate methods. | The sound level meter, which was placed in 120 various stations, detected noise levels ranging from 72-100 dBA. Mean noise intensity for Group 1 and 2 was 81.8 dB (72-83.4) and for Groups 3 and 4 was 92.3 dB (87-99). | There is no evidence that the study conduct or reporting were influenced by a competing interest. | The groups in this study were categorized according to the exposure to noise level and there were no differences in numerator and denominator arising from a mismatch between the definitions. | I judge the study to be free of other potential sources of bias. |

Risk of bias table, Attarchi 2013

| **Risk of bias domain** | **Bias in selection of participants into the study (or selection bias)** | **Performance bias** | **Bias due to exposure misclassification** | **Bias due to incomplete exposure data** | **Bias due to selective reporting of exposures** | **Bias due to conflicts of interest** | **Bias due to differences in numerator and denominator** | **Other biases** |
| --- | --- | --- | --- | --- | --- | --- | --- | --- |
| **Rating** | **LOW** | **LOW** | **PROBABLY LOW** | **PROBABLY LOW** | **HIGH** | **LOW** | **LOW** | **LOW** |
| **Justification for rating** | The study records provide sufficient detail for assessing risk of bias, including the: target population; study sample; criteria for eligibility for individuals to participate in the study; sampling, recruitment and enrolment procedures; rate of participation in the study; and rate of participation in the exposure assessment. The data reported in the study records are sufficiently detailed to support the conclusion that exposure in the study sample is representative of exposure in the target population, with similar characteristics reported for both the study sample and the target population. | Exposure assessors and study personnel were not blinded to relevant participant characteristics. However, this lack of blinding is unlikely to have influenced the exposure assessment (i.e., it was likely that exposure was systematically assessed similarly across sub-groups defined by participants characteristics). | Indirect evidence suggests that exposure data are missing in the target population (e.g. there is evidence that exposure data are missing differentially by participant characteristics that may approximate the exposure). | There is insufficient information about the completeness of exposure data to permit a judgment of low risk of bias, but there is indirect evidence, which suggests incomplete exposure data were adequately addressed, as described by the criteria for a judgment of low risk of bias. | There is insufficient information about selective exposure reporting to permit a judgment of low risk of bias, but there is indirect evidence, which suggests the study was free of selective exposure reporting, as described by the criteria for a judgment of low risk of bias. | There is no evidence that the study conduct or reporting were influenced by a competing interest. | I judge the study to be free of other potential sources of bias. | I judge the study to be free of other potential sources of bias. |

Risk of bias table, Bauer 1991

| **Risk of bias domain** | **Bias in selection of participants into the study (or selection bias)** | **Performance bias** | **Bias due to exposure misclassification** | **Bias due to incomplete exposure data** | **Bias due to selective reporting of exposures** | **Bias due to conflicts of interest** | **Bias due to differences in numerator and denominator** | **Other biases** |
| --- | --- | --- | --- | --- | --- | --- | --- | --- |
| **Rating** | **PROBABLY LOW** | **PROBABLY LOW** | **PROBABLY LOW** | **PROBABLY LOW** | **LOW** | **PROBABLY LOW** | **PROBABLY LOW** | **LOW** |
| **Justification for rating** | Although the criteria of all excluded participants were not evident, the final study number participants was an adequate representation of the target population and participant inclusion and exclusion criteria were appropriately defined. | The information on blinding is insufficient to permit a rating of low risk of bias. However, indirect evidence suggests that the exposure assessors and study personnel were adequately blinded. | Technical devices seem to follow standard requirements and were properly calibrated. Furthermore, the exposure assessment appropriately took into account the adoption of protective gear and other preventive strategies. | The proportion of study participants who participated in the exposure assessment was acceptable and the reasons for non-participation in exposure assessment were acceptable. | Direct evidence suggests that exposure was consistently assessed (i.e., under the same method and time-frame) using well-established methods that directly measure exposure; the exposure measurement assessed relevant levels of the exposure over relevant reporting periods. | Although nothing was declared about conflicts of interest, there is no evidence that the study conduct or reporting were influenced by a competing interest. | The proportion of persons invited to participate in the study who did participate in the study was acceptable. | I judge the study to be free of other potential sources of bias. |

Risk of bias table, Cantley 2015

| **Risk of bias domain** | **Bias in selection of participants into the study (or selection bias)** | **Performance bias** | **Bias due to exposure misclassification** | **Bias due to incomplete exposure data** | **Bias due to selective reporting of exposures** | **Bias due to conflicts of interest** | **Bias due to differences in numerator and denominator** | **Other biases** |
| --- | --- | --- | --- | --- | --- | --- | --- | --- |
| **Rating** | **LOW** | **LOW** | **LOW** | **LOW** | **LOW** | **PROBABLY HIGH** | **LOW** | **LOW** |
| **Justification for rating** | The descriptions and/or data suggest the potential for selection, and there was no information suggesting that potential selection was differential across sub-groups defined by exposure levels. However drivers of selection were well-understood, these drivers of selection were measured in the dataset, and appropriate post-hoe statistical methods were used to control for potential selection bias. | Noise exposure assessed by the company's database. | Direct evidence suggest that the method adopted in the study for the exposure assessment and assignment produces valid, accurate and reliable exposure data. | There were no or minimal incomplete exposure data, or any incomplete exposure data have been imputed using appropriate methods. | There was a pre-published protocol, and the prevalence of all exposures and exposure categories has been reported as pre-specified in the protocol. | There is no evidence that the study conduct or reporting were influenced by a competing interest, however is evidence of financial support and a contract with Alcoa Inc. | The groups in this study were categorized according to the exposure to noise level and there were no differences in numerator and denominator arising from a mismatch between the definitions. | I judge the study to be free of other potential sources of bias. |

Risk of bias table, Chang 2003

| **Risk of bias domain** | **Bias in selection of participants into the study (or selection bias)** | **Performance bias** | **Bias due to exposure misclassification** | **Bias due to incomplete exposure data** | **Bias due to selective reporting of exposures** | **Bias due to conflicts of interest** | **Bias due to differences in numerator and denominator** | **Other biases** |
| --- | --- | --- | --- | --- | --- | --- | --- | --- |
| **Rating** | **PROBABLY HIGH** | **PROBABLY LOW** | **PROBABLY LOW** | **PROBABLY LOW** | **PROBABLY LOW** | **PROBABLY LOW** | **PROBABLY HIGH** | **LOW** |
| **Justification for rating** | Although the workers in the study are from automobile manufacturing company, the sample size is too small for adequate representation of the target population. | The study sample was drawn at random. | Indirect evidence suggests that the exposure was consistently assessed using well-established methods that directly measure exposure. | Table 1 exhibits a brief description of all 15 exposed group subjects (mean and standard deviation). | The exposure measurement assessed relevant levels of the exposure over relevant reporting periods. | Although nothing was declared about conflicts of interest, there is no evidence that the study conduct or reporting were influenced by a competing interest. | The sum of subjects of "high exposed" and "low exposed" groups was equal 20. The proportion of exposed participants (15) and sample size (20) seems inappropriate. | I judge the study to be free of other potential sources of bias. |

Risk of bias table, Chang 2009

| **Risk of bias domain** | **Bias in selection of participants into the study (or selection bias)** | **Performance bias** | **Bias due to exposure misclassification** | **Bias due to incomplete exposure data** | **Bias due to selective reporting of exposures** | **Bias due to conflicts of interest** | **Bias due to differences in numerator and denominator** | **Other biases** |
| --- | --- | --- | --- | --- | --- | --- | --- | --- |
| **Rating** | **PROBABLY LOW** | **PROBABLY LOW** | **PROBABLY LOW** | **LOW** | **PROBABLY LOW** | **LOW** | **PROBABLY LOW** | **LOW** |
| **Justification for rating** | The sampling frame seems representative of the target population. | The information on blinding is insufficient to permit a rating of low risk of bias. However, indirect evidence suggests that the exposure assessors and study personnel were adequately blinded. | Indirect evidence suggests that the exposure was consistently assessed using well-established methods that directly measure exposure. | Data on exposure status (or level) seem complete for eligible participants. | Data were collected directly from the participants. | Study authors make a claim denying conflicts of interest. | The proportion of persons invited to participate in the study who did participate in the study was acceptable. | I judge the study to be free of other potential sources of bias. |

Risk of bias table, Chang 2012

| **Risk of bias domain** | **Bias in selection of participants into the study (or selection bias)** | **Performance bias** | **Bias due to exposure misclassification** | **Bias due to incomplete exposure data** | **Bias due to selective reporting of exposures** | **Bias due to conflicts of interest** | **Bias due to differences in numerator and denominator** | **Other biases** |
| --- | --- | --- | --- | --- | --- | --- | --- | --- |
| **Rating** | **LOW** | **LOW** | **LOW** | **LOW** | **LOW** | **PROBABLY HIGH** | **LOW** | **LOW** |
| **Justification for rating** | The descriptions and/or data indicated above do suggest the potential for selection bias. However, adequate information was given tto judge any potential selection as not differential across sub-groups defined by exposure levels. | The exposure assessment was based on the environmental noise assessment and study personnel were adequately blinded or blinding was unlikely to influence exposure assessment, as described by the criteria for a rating of low risk of bias. | Direct evidence suggests that the method adopted in the study for the exposure assessment and assignment produces valid, accurate and reliable exposure data, ideally based on direct quantitative exposure measures. | There were no or minimal incomplete exposure data, or any incomplete exposure data have been imputed using appropriate methods. | The study findings suggest that occupational noise exposure above 80 dBA for specific periods may be associated with hypertension, and noise frequency at 4000 Hz may have the greatest effect on hypertension. | There is no evidence that the study conduct or reporting were influenced by a competing interest but there is evidence of receipt of payment from the government. | The groups in this study were categorized according to the exposure to noise level and there were no differences in numerator and denominator arising from a mismatch between the definitions. | I judge the study to be free of other potential sources of bias. |

Risk of bias table, Chen 2017

| **Risk of bias domain** | **Bias in selection of participants into the study (or selection bias)** | **Performance bias** | **Bias due to exposure misclassification** | **Bias due to incomplete exposure data** | **Bias due to selective reporting of exposures** | **Bias due to conflicts of interest** | **Bias due to differences in numerator and denominator** | **Other biases** |
| --- | --- | --- | --- | --- | --- | --- | --- | --- |
| **Rating** | **LOW** | **LOW** | **PROBABLY LOW** | **PROBABLY HIGH** | **HIGH** | **LOW** | **PROBABLY HIGH** | **LOW** |
| **Justification for rating** | The study records provide sufficient detail for assessing risk of bias, including the: target population; study sample; criteria for eligibility for individuals to participate in the study; sampling, recruitment and enrolment procedures; rate of participation in the study; and rate of participation in the exposure assessment. The data reported in the study records are sufficiently detailed to support the conclusion that exposure in the study sample is representative of exposure in the target population, with similar characteristics reported for both the study sample and the target population | Exposure assessors and study personnel were not blinded to relevant participant characteristics. However, this lack of blinding is unlikely to have influenced the exposure assessment (i.e., it was likely that exposure was systematically assessed similarly across sub-groups defined by participants characteristics). | There is insufficient information about the completeness of exposure data to permit a judgment of low risk of bias, but there is indirect evidence, which suggests incomplete exposure data were adequately addressed, as described by the criteria for a judgment of low risk of bias. | Indirect evidence suggests that exposure data are missing in the target population (e.g. there is evidence that exposure data are missing differentially by participant characteristics that may approximate the exposure). | There is insufficient information about selective exposure reporting to permit a judgment of low risk of bias, but there is indirect evidence, which suggests the study was free of selective exposure reporting, as described by the criteria for a judgment of low risk of bias. | There is no evidence that the study conduct or reporting were influenced by a competing interest. | There is insufficient information to permit a rating of high risk of bias, but there is indirect evidence, which suggests the study was not free of other potential sources of bias, as described by the criteria for a rating of high risk of bias. | I judge the study to be free of other potential sources of bias |

Risk of bias table, de Souza 2015

| **Risk of bias domain** | **Bias in selection of participants into the study (or selection bias)** | **Performance bias** | **Bias due to exposure misclassification** | **Bias due to incomplete exposure data** | **Bias due to selective reporting of exposures** | **Bias due to conflicts of interest** | **Bias due to differences in numerator and denominator** | **Other biases** |
| --- | --- | --- | --- | --- | --- | --- | --- | --- |
| **Rating** | **PROBABLY LOW** | **PROBABLY LOW** | **PROBABLY HIGH** | **PROBABLY HIGH** | **PROBABLY HIGH** | **LOW** | **LOW** | **LOW** |
| **Justification for rating** | Information about selection does not suffice to permit judgement of the risk of bias to be low. Indirect evidence (see Definitions) suggests that inclusion/exclusion criteria, recruitment and enrolment procedures, and participation/ response rates were similar across groups as described by the criteria for a rating of low risk of bias. | The information on blinding is insufficient to permit a rating of low risk of bias. However, indirect evidence suggests that the exposure assessors and study personnel were adequately blinded or blinding was unlikely to influence exposure assessment, as described by the criteria for a rating of low risk of bias. | I judge there to be insufficient information about the exposure assessment and assignment methods to permit a rating of high risk of bias, but there is indirect evidence that suggests that methods were not robust, as described by the criteria for a rating of high risk of bias: | There is insufficient information about incomplete exposure data to permit a judgment of high risk of bias, but there is indirect evidence that incomplete exposure data were not adequately addressed, as described by the criteria for a judgment of high risk of bias. | There is insufficient information about selective exposure reporting to permit a judgment of high risk of bias, but there is indirect evidence, which suggests the study was not free of selective exposure reporting, as described by the criteria for a judgment of high risk of bias. | There is no evidence that the study conduct or reporting were influenced by a competing interest. | I judge the study to be free of other potential sources of bias. | I judge the study to be free of other potential sources of bias. |

Risk of bias table, Du 2007

| **Risk of bias domain** | **Bias in selection of participants into the study (or selection bias)** | **Performance bias** | **Bias due to exposure misclassification** | **Bias due to incomplete exposure data** | **Bias due to selective reporting of exposures** | **Bias due to conflicts of interest** | **Bias due to differences in numerator and denominator** | **Other biases** |
| --- | --- | --- | --- | --- | --- | --- | --- | --- |
| **Rating** | **PROBABLY HIGH** | **PROBABLY HIGH** | **PROBABLY LOW** | **LOW** | **LOW** | **LOW** | **PROBABLY LOW** | **LOW** |
| **Justification for rating** | The article does not provide information about how the study selected the workers among exposed and non-exposed groups. Therefore, there is a lack of evidence that the study population can reflect the target population accurately. | The exposure group was selected by the author before measurement of noise , but the result of the measurement suggest that 118 workers in the exposure group were exposed to noise below 80 dB(A). | The noise of the textile workshop of textile enterprises is steady-state noise. The laborers are basically fixed-point operations, and the investigation shows that the laborers work 8 hours a day. Therefore, the detection of noise at fixed-point places is basically close to the equivalent sound level of 8 hours. | There were no or minimal incomplete exposure data or any incomplete exposure data have been imputed using appropriate methods. | The article does not include a pre-plan, but reports on all types of exposure and exposure in the article. | The author was working in Fuxin Municipal Center for Disease Control and Prevention and Liaoning Provincial Occupational Disease Prevention and Treatment Center. They are all technical institutions under the government department. Because the regulation of the government and the CDC, the risk of potential interest is low. | The numerator is defined as the number of workers who exposed the noise. The denominator is defined as who is exposed with noise. | I judge the study to be free of other potential sources of bias. |

Risk of bias table, EWCS 2015

| **Risk of bias domain** | **Bias in selection of participants into the study (or selection bias)** | **Performance bias** | **Bias due to exposure misclassification** | **Bias due to incomplete exposure data** | **Bias due to selective reporting of exposures** | **Bias due to conflicts of interest** | **Bias due to differences in numerator and denominator** | **Other biases** |
| --- | --- | --- | --- | --- | --- | --- | --- | --- |
| **Rating** | **LOW** | **LOW** | **PROBABLY LOW** | **LOW** | **LOW** | **LOW** | **LOW** | **LOW** |
| **Justification for rating** | The descriptions and/or data as indicated above suggest the potential for selection, and there was no information suggesting that potential selection was not differential across sub-groups defined by exposure levels. However drivers of selection were well-understood, these drives of selection were measured in the data set, and appropriate post-hoe statistical methods were used to control for potential selection bias. | Noise exposure assessed by the company's database. | Insufficient information exists about the exposure assessment and assignment method to permit you to rate the risk of bias as low. However, there is indirect evidence that the methods were valid, accurate and reliable, as described by the criteria for a rating of low risk of bias: Indirect evidence suggests that the exposure was assessed using validated methods that directly measure exposure. | There were no or minimal incomplete exposure data or any incomplete exposure data have been imputed using appropriate methods. | There was a pre-published protocol, and the prevalence of all exposures and exposure categories has been reported as pre-specified in the protocol. | There is no evidence that the study conduct or reporting were influenced by a competing interest. | The groups in this study were categorized according to the exposure to noise level and there were no differences in numerator and denominator arising from a mismatch between the definitions. | I judge the study to be free of other potential sources of bias |

Risk of bias table, Hu 2005

| **Risk of bias domain** | **Bias in selection of participants into the study (or selection bias)** | **Performance bias** | **Bias due to exposure misclassification** | **Bias due to incomplete exposure data** | **Bias due to selective reporting of exposures** | **Bias due to conflicts of interest** | **Bias due to differences in numerator and denominator** | **Other biases** |
| --- | --- | --- | --- | --- | --- | --- | --- | --- |
| **Rating** | **PROBABLY HIGH** | **LOW** | **LOW** | **LOW** | **LOW** | **LOW** | **LOW** | **LOW** |
| **Justification for rating** | The study does not mention the selection method and workers who meet the include criteria, and the recruitment and enrolment procedures is might not representative of the target population. | All participants were selected by researcher according to their job title, but the lack of blinding is unlikely to have influenced the exposure assessment. | The exposure assessment was based on the environmental noise measurement. However the workers were stable in their working position, therefore the environmental noise measure could reflect their individual noise exposure. | There were no or minimal incomplete exposure data or any incomplete exposure data have been imputed using appropriate methods. | The article does not include a pre-plan, but reports on all types of exposure and exposure in the article. | The author is working in Zhongshan City Center for Disease Control and Prevention. It is a technical institution under the government department. Because the regulation of the government, the risk of potential interest is low. | The numerator is defined as the number of workers who exposed the noise above 85dBA. | I judge the study to be free of other potential sources of bias. |

Risk of bias table, Hughes 2013

| **Risk of bias domain** | **Bias in selection of participants into the study (or selection bias)** | **Performance bias** | **Bias due to exposure misclassification** | **Bias due to incomplete exposure data** | **Bias due to selective reporting of exposures** | **Bias due to conflicts of interest** | **Bias due to differences in numerator and denominator** | **Other biases** |
| --- | --- | --- | --- | --- | --- | --- | --- | --- |
| **Rating** | **PROBABLY HIGH** | **PROBABLY LOW** | **PROBABLY HIGH** | **PROBABLY HIGH** | **PROBABLY HIGH** | **LOW** | **LOW** | **LOW** |
| **Justification for rating** | The study's eligibility criteria systematically excluded participants in the study sample such that exposure in the study sample may not represent exposure in the target population. | The exposure assessment was based on data collected from existing audiometric examinations, therefore the information on blinding is insufficient to permit a rating of low risk of bias. However, indirect evidence suggests that the exposure assessors and study personnel were adequately blinded or blinding was unlikely to influence exposure assessment, as described by the criteria for a rating of low risk of bias. | As describe in the report, noise exposure data may overestimate the true exposures to noise among the subjects due to use of hearing protection as per occupational standards and HCPs regulations. | As stated in the report because noise-exposed personnel were most likely wearing hearing protection when exposed to hazardous occupational noise sources, the differences in true exposures between the study's four noise groups may have been modest, therefore diminishing the ability to detect a difference in hearing loss between exposure groups. | Uncontrolled confounders and possible misclassifications due to employment status may introduce some degree of uncertainty. | There is no evidence that the study conduct or reporting were influenced by a competing interest. | The groups in this study were categorized according to the exposure to noise level and there are no differences in numerator and denominator arising from a mismatch between the definitions. | I judge the study to be free of other potential sources of bias. |

Risk of bias table, Inoue 2005

| **Risk of bias domain** | **Bias in selection of participants into the study (or selection bias)** | **Performance bias** | **Bias due to exposure misclassification** | **Bias due to incomplete exposure data** | **Bias due to selective reporting of exposures** | **Bias due to conflicts of interest** | **Bias due to differences in numerator and denominator** | **Other biases** |
| --- | --- | --- | --- | --- | --- | --- | --- | --- |
| **Rating** | **HIGH** | **PROBABLY LOW** | **PROBABLY HIGH** | **PROBABLY HIGH** | **PROBABLY HIGH** | **LOW** | **HIGH** | **LOW** |
| **Justification for rating** | The descriptions of the target population, inclusion/exclusion criteria, recruitment and enrolment procedures, participation/ response rates and/or data on the distribution of relevant study sample and population characteristics suggest that the risk of selection bias was substantial. | The information on blinding is insufficient to permit a rating of low risk of bias. However, indirect evidence suggests that the exposure assessors and study personnel were adequately blinded or blinding was unlikely to influence exposure assessment, as described by the criteria for a rating of low risk of bias. | You judge there to be insufficient information about the exposure assessment and assignment methods to permit a rating of high risk of bias, but there is indirect evidence that suggests that methods were not robust, as described by the criteria for a rating of high risk of bias: | There is insufficient information about incomplete exposure data to permit a judgment of high risk of bias, but there is indirect evidence that incomplete exposure data were not adequately addressed, as described by the criteria for a judgment of high risk of bias. | There is insufficient information about selective exposure reporting to permit a judgment of high risk of bias, but there is indirect evidence, which suggests the study was not free of selective exposure reporting, as described by the criteria for a judgment of high risk of bias. | There is no evidence that the study conduct or reporting were influenced by a competing interest. | The numerator and denominator are defined and/or counted differently, and/or the shortest prevalence period was inappropriate (e.g. too short to detect exposure). | I judge the study to be free of other potential sources of bias. |

Risk of bias table, Ivanovich 1994

| **Risk of bias domain** | **Bias in selection of participants into the study (or selection bias)** | **Performance bias** | **Bias due to exposure misclassification** | **Bias due to incomplete exposure data** | **Bias due to selective reporting of exposures** | **Bias due to conflicts of interest** | **Bias due to differences in numerator and denominator** | **Other biases** |
| --- | --- | --- | --- | --- | --- | --- | --- | --- |
| **Rating** | **PROBABLY HIGH** | **PROBABLY HIGH** | **LOW** | **LOW** | **LOW** | **LOW** | **LOW** | **LOW** |
| **Justification for rating** | The study's eligibility criteria systematically excluded participants in the study sample such that exposure in the study sample may not represent exposure in the target population. | In the study, mean hearing level at each tested frequency was compared between similar jobs activities in the different departments. It was revealed a background noise in the range of 57.4 - 70.6 dBA in the International Department, 70.6 - 71.7 dBA in the Intercity Department and 68.7 - 70.1 dBA in the Information Department and highest intensities of 63.5, 62.1 and 58.2 dB for frequencies of 0.5, 1 and 2 kHz. The registered noise (Leq) from the inner telephone receiver microphone (type Star-set) was in the range of 78.4 - 86.6 dBA. Peaks of 90-96 dBA were registered during different working operations ("dialing", "speaking", "fax", "occupied") with duration less than 5s and stochastic appearance during the 15 min measuring periods. | Direct evidence suggests that the method adopted in the study for the exposure assessment and assignment produces valid, accurate and reliable exposure data, ideally based on direct quantitative exposure measures. | There were no or minimal incomplete exposure data or any incomplete exposure data have been imputed using appropriate methods. | The article does not include a pre-plan, but reports on all types of exposure and exposure in the article. | There is no evidence that the study conduct or reporting were influenced by a competing interest. | The groups in this study were categorized according to the exposure to noise level and there were no differences in numerator and denominator arising from a mismatch between the definitions. | I judge the study to be free of other potential sources of bias |

Risk of bias table, Johnson 2006

| **Risk of bias domain** | **Bias in selection of participants into the study (or selection bias)** | **Performance bias** | **Bias due to exposure misclassification** | **Bias due to incomplete exposure data** | **Bias due to selective reporting of exposures** | **Bias due to conflicts of interest** | **Bias due to differences in numerator and denominator** | **Other biases** |
| --- | --- | --- | --- | --- | --- | --- | --- | --- |
| **Rating** | **PROBABLY HIGH** | **PROBABLY LOW** | **LOW** | **LOW** | **LOW** | **PROBABLY LOW** | **LOW** | **LOW** |
| **Justification for rating** | The study's eligibility criteria systematically excluded participants in the study sample such that exposure in the study sample may not represent exposure in the target population. | Noise exposure assessment was described earlier in Morata et al - Audiometric findings in workers exposed to low levels of styrene and noise, 2002. | Direct evidence suggest that the method adopted in the study for the exposure assessment and assignment produces valid, accurate and reliable exposure data. | There were no or minimal incomplete exposure data, or any incomplete exposure data have been imputed using appropriate methods. | There was a pre-published protocol, and the prevalence of all exposures and exposure categories has been reported as pre-specified in the protocol. | There is no evidence that the study conduct or reporting were influenced by a competing interest, however is evidence of financial support. | The groups in this study were categorized according to the exposure to noise level and there were no differences in numerator and denominator arising from a mismatch between the definitions. | I judge the study to be free of other potential sources of bias. |

Risk of bias table, Kock 2004

| **Risk of bias domain** | **Bias in selection of participants into the study (or selection bias)** | **Performance bias** | **Bias due to exposure misclassification** | **Bias due to incomplete exposure data** | **Bias due to selective reporting of exposures** | **Bias due to conflicts of interest** | **Bias due to differences in numerator and denominator** | **Other biases** |
| --- | --- | --- | --- | --- | --- | --- | --- | --- |
| **Rating** | **PROBABLY HIGH** | **LOW** | **LOW** | **LOW** | **LOW** | **PROBABLY HIGH** | **LOW** | **LOW** |
| **Justification for rating** | The study's eligibility criteria systematically excluded participants in the study sample such that exposure in the study sample may not represent exposure in the target population. | The exposure assessment was based on the environmental noise assessment and study personnel were adequately blinded or blinding was unlikely to influence exposure assessment, as described by the criteria for a rating of low risk of bias. | Direct evidence suggests that the method adopted in the study for the exposure assessment and assignment produces valid, accurate and reliable exposure data, ideally based on direct quantitative exposure measures. | There were no or minimal incomplete exposure data or any incomplete exposure data have been imputed using appropriate methods. | In the study, the noise levels ranged between 83.7 dB(A)(95% CI 83.3 to 84.1) in the selected industries; 69.9 dB(A)(95% CI 68.8 to 71.0) for the residents and office workers and some 50% of the workers were exposed to more than 85 dB(A) and some 20% to more than 90 dB(A) in several industries. | There is no evidence that the study conduct or reporting were influenced by a competing interest but there is evidence of receipt of payment from the government. | The groups in this study were categorized according to the exposure to noise level and there were no differences in numerator and denominator arising from a mismatch between the definitions. | I judge the study to be free of other potential sources of bias |

Risk of bias table, Kovacevic 2006

| **Risk of bias domain** | **Bias in selection of participants into the study (or selection bias)** | **Performance bias** | **Bias due to exposure misclassification** | **Bias due to incomplete exposure data** | **Bias due to selective reporting of exposures** | **Bias due to conflicts of interest** | **Bias due to differences in numerator and denominator** | **Other biases** |
| --- | --- | --- | --- | --- | --- | --- | --- | --- |
| **Rating** | **PROBABLY HIGH** | **PROBABLY LOW** | **LOW** | **PROBABLY HIGH** | **PROBABLY HIGH** | **PROBABLY LOW** | **HIGH** | **LOW** |
| **Justification for rating** | Information about selection does not suffice to permit you to judge the risk of bias to be high. Indirect evidence suggests that inclusion/exclusion criteria, recruitment and enrolment procedures, and participation/response rates differed across groups, as described by the criteria for a rating of high risk of bias. | The information on blinding is insufficient to permit a rating of low risk of bias. However, indirect evidence suggests that the exposure assessors and study personnel were adequately blinded or blinding was unlikely to influence exposure assessment, as described by the criteria for a rating of low risk of bias. | Direct evidence suggests that the method adopted in the study for the exposure assessment and assignment produces valid, accurate and reliable exposure data, ideally based on direct quantitative exposure measures. | There is insufficient information about incomplete exposure data to permit a judgment of high risk of bias, but there is indirect evidence that incomplete exposure data were not adequately addressed, as described by the criteria for a judgment of high risk of bias. | There is insufficient information about selective exposure reporting to permit a judgment of high risk of bias, but there is indirect evidence, which suggests the study was not free of selective exposure reporting, as described by the criteria for a judgment of high risk of bias. | There is insufficient information to permit a rating of low risk of bias, but there is indirect evidence that the study conduct or reporting were not influenced by a competing interest. | The authors didn't inform which is a total population of the industry. | I judge the study to be free of other potential sources of bias. |

Risk of bias table, Landen 2004

| **Risk of bias domain** | **Bias in selection of participants into the study (or selection bias)** | **Performance bias** | **Bias due to exposure misclassification** | **Bias due to incomplete exposure data** | **Bias due to selective reporting of exposures** | **Bias due to conflicts of interest** | **Bias due to differences in numerator and denominator** | **Other biases** |
| --- | --- | --- | --- | --- | --- | --- | --- | --- |
| **Rating** | **HIGH** | **LOW** | **LOW** | **HIGH** | **HIGH** | **LOW** | **PROBABLY HIGH** | **PROBABLY HIGH** |
| **Justification for rating** | The descriptions of the target population, inclusion/exclusion criteria, recruitment and enrolment procedures, participation/response rates and/or data on the distribution of relevant study sample and population characteristics suggest that the risk of selection bias was substantial; | Exposure assessors and study personnel were not blinded to relevant participant characteristics. However, this lack of blinding is unlikely to have influenced the exposure assessment (e.g., it was likely that exposure was systematically assessed similarly across sub-groups defined by participants characteristics); | Direct evidence suggests that the method adopted in the study for the exposure assessment and assignment produces valid, accurate and reliable exposure data, ideally based on direct quantitative exposure measures. | Participation in the study was so low that this could have introduced bias. | There was no pre-published protocol, and there was direct evidence that the study was not free of selective exposure reporting. | There is no evidence that the study conduct or reporting were influenced by a competing interest. | There is insufficient information to permit a rating of high risk of bias, but there is indirect evidence, which suggests the study was not free of other potential sources of bias, as described by the criteria for a rating of high risk of bias. The authors do not report the total population of sand and gravel miners. | There is insufficient information to permit a rating of high risk of bias, but there is indirect evidence, which suggests the study was not free of other potential sources of bias, as described by the criteria for a rating of high risk of bias. |

Risk of bias table, Lee 1999

| **Risk of bias domain** | **Bias in selection of participants into the study (or selection bias)** | **Performance bias** | **Bias due to exposure misclassification** | **Bias due to incomplete exposure data** | **Bias due to selective reporting of exposures** | **Bias due to conflicts of interest** | **Bias due to differences in numerator and denominator** | **Other biases** |
| --- | --- | --- | --- | --- | --- | --- | --- | --- |
| **Rating** | **PROBABLY HIGH** | **LOW** | **LOW** | **LOW** | **LOW** | **PROBABLY LOW** | **LOW** | **LOW** |
| **Justification for rating** | The study's eligibility criteria systematically excluded participants in the study sample such that exposure in the study sample may not represent exposure in the target population | The exposure assessment was based on the environmental noise assessment and study personnel were adequately blinded or blinding was unlikely to influence exposure assessment, as described by the criteria for a rating of low risk of bias. | Direct evidence suggests that the method adopted in the study for the exposure assessment and assignment produces valid, accurate and reliable exposure data, ideally based on direct quantitative exposure measures. | There were no or minimal incomplete exposure data or any incomplete exposure data have been imputed using appropriate methods. | It is between 10pm and 3am that the noise exposure level goes above 85dBA. The range of exposure to above 85dBA for the employees is 3.6 to 6.9 hours with a mean of 5.1 hours. All the occupational groups are however exposed to a noise level of at least 89dBA Leq for their whole work shift (Table 1). | There is no evidence that the study conduct or reporting were influenced by a competing interest. | The groups in this study were categorized according to the exposure to noise level and there were no differences in numerator and denominator arising from a mismatch between the definitions. | I judge the study to be free of other potential sources of bias. |

Risk of bias table, Lee 2009

| **Risk of bias domain** | **Bias in selection of participants into the study (or selection bias)** | **Performance bias** | **Bias due to exposure misclassification** | **Bias due to incomplete exposure data** | **Bias due to selective reporting of exposures** | **Bias due to conflicts of interest** | **Bias due to differences in numerator and denominator** | **Other biases** |
| --- | --- | --- | --- | --- | --- | --- | --- | --- |
| **Rating** | **HIGH** | **PROBABLY HIGH** | **HIGH** | **HIGH** | **PROBABLY HIGH** | **LOW** | **LOW** | **LOW** |
| **Justification for rating** | The study records provide sufficient detail for assessing risk of bias, including the: target population; study sample; criteria for eligibility for individuals to participate in the study; sampling, recruitment and enrolment procedures; rate of participation in the study; and rate of participation in the exposure assessment. The data reported in the study records are sufficiently detailed to support the conclusion that exposure in the study sample is representative of exposure in the target population, with similar characteristics reported for both the study sample and the target population. | The information on blinding is insufficient to permit a rating of low risk of bias. However, indirect evidence suggests that the exposure assessors and study personnel were adequately blinded or blinding was unlikely to influence exposure assessment, as described by the criteria for a rating of low risk of bias. | Direct evidence suggests that the method adopted in the study for the exposure assessment and assignment produces valid, accurate and reliable exposure data, ideally based on direct quantitative exposure measures. | There is insufficient information about the completeness of exposure data to permit a judgment of low risk of bias, but there is indirect evidence, which suggests incomplete exposure data were adequately addressed, as described by the criteria for a judgment of low risk of bias. | There is insufficient information about selective exposure reporting to permit a judgment of low risk of bias, but there is indirect evidence, which suggests the study was free of selective exposure reporting, as described by the criteria for a judgment of low risk of bias. | There is no evidence that the study conduct or reporting were influenced by a competing interest. | I judge the study to be free of other potential sources of bias. | I judge the study to be free of other potential sources of bias. |

Risk of bias table, Liu 2015

| **Risk of bias domain** | **Bias in selection of participants into the study (or selection bias)** | **Performance bias** | **Bias due to exposure misclassification** | **Bias due to incomplete exposure data** | **Bias due to selective reporting of exposures** | **Bias due to conflicts of interest** | **Bias due to differences in numerator and denominator** | **Other biases** |
| --- | --- | --- | --- | --- | --- | --- | --- | --- |
| **Rating** | **PROBABLY HIGH** | **HIGH** | **PROBABLY HIGH** | **PROBABLY LOW** | **LOW** | **LOW** | **HIGH** | **LOW** |
| **Justification for rating** | The study only recruited the workers who had worked for at least 1 year, and no other information about the total workers in these MSWLs. Meanwhile, The study’s eligibility criteria systematically excluded participants in the study sample such that exposure in the study sample may not represent exposure in the target population. | The 3 groups in the study were defined based on the specific work sites in the MSWLs where they mainly worked. The environmental noise levels were only reported by groups.  Therefore, exposure assessors and study personnel were not blinded, and the exposure assessment was likely to be influenced by the lack of blinding. | The exposure assessment was based on environmental noise from the general background at the workplaces. There is no information about the use of personal protective equipment. | There were no or minimal incomplete exposure data or any incomplete exposure data have been imputed using appropriate methods (all data reported in Table 2). | There is insufficient information about selective exposure reporting to permit a judgment of low risk of bias, but there is indirect evidence which suggests the study was free of selective exposure reporting (all data reported in Table 2). | The authors have declared that no competing interests exist. | The numerator should be the number of workers who exposed to noise above 80dBA. However, the noise levels were only reported by groups in this articles. Only several noise levels at some sites where workers in group 3 were above 80 dBA,and The mean time-weighted noise exposure of bulldozer drivers and compacting machine operators were 95.1 dB and 91.1 dB. There is no other information about the exposure condition of other workers. | I judge the study to be free of other potential sources of bias. |

Risk of bias table, Lv 2003

| **Risk of bias domain** | **Bias in selection of participants into the study (or selection bias)** | **Performance bias** | **Bias due to exposure misclassification** | **Bias due to incomplete exposure data** | **Bias due to selective reporting of exposures** | **Bias due to conflicts of interest** | **Bias due to differences in numerator and denominator** | **Other biases** |
| --- | --- | --- | --- | --- | --- | --- | --- | --- |
| **Rating** | **HIGH** | **HIGH** | **LOW** | **LOW** | **PROBABLY HIGH** | **LOW** | **LOW** | **LOW** |
| **Justification for rating** | The study only selected workers who exposed to noise, and there is no information about total number of workers in the factories. Therefore, the recruitment and enrolment procedures are not representative of the target population. | All participants were selected by researcher according to their job title, and were assumed that they are all exposed to a high level noise by researcher. | The exposure assessment was based on individual noise measurement. | There were no or minimal incomplete exposure data or any incomplete exposure data have been imputed using appropriate methods (all data reported in Table 1 and table 2). | The article only shows the numbers of worker exposed to noise below 85dBA, but the criteria in the SR8 is 80dBA. | This article was supported by funds. Their research behavior or reports were not affected by competitive interests. | The definition of numerator and denominator are based on the exposure level of noise. | I judge the study to be free of other potential sources of bias. |

Risk of bias table, Morata 1997

| **Risk of bias domain** | **Bias in selection of participants into the study (or selection bias)** | **Performance bias** | **Bias due to exposure misclassification** | **Bias due to incomplete exposure data** | **Bias due to selective reporting of exposures** | **Bias due to conflicts of interest** | **Bias due to differences in numerator and denominator** | **Other biases** |
| --- | --- | --- | --- | --- | --- | --- | --- | --- |
| **Rating** | **PROBABLY HIGH** | **LOW** | **LOW** | **LOW** | **LOW** | **PROBABLY HIGH** | **LOW** | **LOW** |
| **Justification for rating** | The study's eligibility criteria systematically excluded participants in the study sample such that exposure in the study sample may not represent exposure in the target population. | The exposure assessment was based on the environmental noise assessment and study personnel were adequately blinded or blinding was unlikely to influence exposure assessment, as described by the criteria for a rating of low risk of bias. | Direct evidence suggests that the method adopted in the study for the exposure assessment and assignment produces valid, accurate and reliable exposure data, ideally based on direct quantitative exposure measures. | There were no or minimal incomplete exposure data or any incomplete exposure data have been imputed using appropriate methods. | Measurements suggested that most exposures to noise and solvents were within exposure limits recommended by international agencies; however, the prevalence for hearing loss within the exposed groups ranged from 42 to 50%, significantly exceeding the 15-30% prevalence observed for unexposed groups. | There is no evidence that the study conduct or reporting were influenced by a competing interest, however there was confirmation that this study received financial support. | The groups in this study were categorized according to the exposure to noise level and there were no differences in numerator and denominator arising from a mismatch between the definitions. | I judge the study to be free of other potential sources of bias |

Risk of bias table, Nazir 2012

| **Risk of bias domain** | **Bias in selection of participants into the study (or selection bias)** | **Performance bias** | **Bias due to exposure misclassification** | **Bias due to incomplete exposure data** | **Bias due to selective reporting of exposures** | **Bias due to conflicts of interest** | **Bias due to differences in numerator and denominator** | **Other biases** |
| --- | --- | --- | --- | --- | --- | --- | --- | --- |
| **Rating** | **PROBABLY HIGH** | **LOW** | **LOW** | **LOW** | **LOW** | **PROBABLY LOW** | **LOW** | **LOW** |
| **Justification for rating** | The study's eligibility criteria systematically excluded participants in the study sample such that exposure in the study sample may not represent exposure in the target population. | The exposure assessment was based on the environmental noise assessment and study personnel were adequately blinded or blinding was unlikely to influence exposure assessment, as described by the criteria for a rating of low risk of bias. | Direct evidence suggests that the method adopted in the study for the exposure assessment and assignment produces valid, accurate and reliable exposure data, ideally based on direct quantitative exposure measures. | There were no or minimal incomplete exposure data or any incomplete exposure data have been imputed using appropriate methods. | In the study, mean hearing level at each tested frequency was compared between occupational noise exposed and non-exposed groups using Student tests. There were a significant difference in the frequency 3 KHz (95% CI=3.4-10.0), 4 KHz (95% CI=3.0-12.5), 6 KHz (95% CI=2.3-13.2) and 8 KHz (95% CI=1.3-11.8) of right ear between the two groups. Unlike right ear, there was only a significant difference in the frequency 3 KHz (95% CI=2.6-9.2) of left ear between the two groups. | There is insufficient information to permit a rating of low risk bias, but there is indirect evidence that the study conduct or reporting were not influenced by a competing interest. | The groups in this study were categorized according to the exposure to noise level and there were no differences in numerator and denominator arising from a mismatch between the definitions. | I judge the study to be free of other potential sources of bias |

Risk of bias table, NHANES (1999-2004)

| **Risk of bias domain** | **Bias in selection of participants into the study (or selection bias)** | **Performance bias** | **Bias due to exposure misclassification** | **Bias due to incomplete exposure data** | **Bias due to selective reporting of exposures** | **Bias due to conflicts of interest** | **Bias due to differences in numerator and denominator** | **Other biases** |
| --- | --- | --- | --- | --- | --- | --- | --- | --- |
| **Rating** | **LOW** | **LOW** | **PROBABLY LOW** | **LOW** | **LOW** | **LOW** | **LOW** | **LOW** |
| **Justification for rating** | The descriptions and/or data as indicated above suggest the potential for selection, and there was no information suggesting that potential selection was not differential across sub-groups defined by exposure levels. However drivers of selection were well-understood, these drives of selection were measured in the data set, and appropriate post-hoe statistical methods were used to control for potential selection bias. | Noise exposure assessed by the company's database. | Insufficient information exists about the exposure assessment and assignment method to permit you to rate the risk of bias as low. However, there is indirect evidence that the methods were valid, accurate and reliable, as described by the criteria for a rating of low risk of bias: Indirect evidence suggests that the exposure was assessed using validated methods that directly measure exposure. | There were no or minimal incomplete exposure data or any incomplete exposure data have been imputed using appropriate methods. | There was a pre-published protocol, and the prevalence of all exposures and exposure categories has been reported as pre-specified in the protocol. | There is no evidence that the study conduct or reporting were influenced by a competing interest. | The groups in this study were categorized according to the exposure to noise level and there were no differences in numerator and denominator arising from a mismatch between the definitions. | I judge the study to be free of other potential sources of bias |

Risk of bias table, NHIS (2007)

| **Risk of bias domain** | **Bias in selection of participants into the study (or selection bias)** | **Performance bias** | **Bias due to exposure misclassification** | **Bias due to incomplete exposure data** | **Bias due to selective reporting of exposures** | **Bias due to conflicts of interest** | **Bias due to differences in numerator and denominator** | **Other biases** |
| --- | --- | --- | --- | --- | --- | --- | --- | --- |
| **Rating** | **LOW** | **LOW** | **PROBABLY LOW** | **LOW** | **LOW** | **LOW** | **LOW** | **LOW** |
| **Justification for rating** | The descriptions and/or data as indicated above suggest the potential for selection, and there was no information suggesting that potential selection was not differential across sub-groups defined by exposure levels. However drivers of selection were well-understood, these drives of selection were measured in the data set, and appropriate post-hoe statistical methods were used to control for potential selection bias. | Noise exposure assessed by the company's database. | Insufficient information exists about the exposure assessment and assignment method to permit you to rate the risk of bias as low. However, there is indirect evidence that the methods were valid, accurate and reliable, as described by the criteria for a rating of low risk of bias: Indirect evidence suggests that the exposure was assessed using validated methods that directly measure exposure. | There were no or minimal incomplete exposure data or any incomplete exposure data have been imputed using appropriate methods. | There was a pre-published protocol, and the prevalence of all exposures and exposure categories has been reported as pre-specified in the protocol. | There is no evidence that the study conduct or reporting were influenced by a competing interest. | The groups in this study were categorized according to the exposure to noise level and there were no differences in numerator and denominator arising from a mismatch between the definitions. | I judge the study to be free of other potential sources of bias |

Risk of bias table, NHIS (2014)

| **Risk of bias domain** | **Bias in selection of participants into the study (or selection bias)** | **Performance bias** | **Bias due to exposure misclassification** | **Bias due to incomplete exposure data** | **Bias due to selective reporting of exposures** | **Bias due to conflicts of interest** | **Bias due to differences in numerator and denominator** | **Other biases** |
| --- | --- | --- | --- | --- | --- | --- | --- | --- |
| **Rating** | **LOW** | **LOW** | **PROBABLY LOW** | **LOW** | **LOW** | **LOW** | **LOW** | **LOW** |
| **Justification for rating** | The descriptions and/or data as indicated above suggest the potential for selection, and there was no information suggesting that potential selection was not differential across sub-groups defined by exposure levels. However drivers of selection were well-understood, these drives of selection were measured in the data set, and appropriate post-hoe statistical methods were used to control for potential selection bias. | Noise exposure assessed by the company's database. | Insufficient information exists about the exposure assessment and assignment method to permit you to rate the risk of bias as low. However, there is indirect evidence that the methods were valid, accurate and reliable, as described by the criteria for a rating of low risk of bias: Indirect evidence suggests that the exposure was assessed using validated methods that directly measure exposure. | There were no or minimal incomplete exposure data or any incomplete exposure data have been imputed using appropriate methods. | There was a pre-published protocol, and the prevalence of all exposures and exposure categories has been reported as pre-specified in the protocol. | There is no evidence that the study conduct or reporting were influenced by a competing interest. | The groups in this study were categorized according to the exposure to noise level and there were no differences in numerator and denominator arising from a mismatch between the definitions. | I judge the study to be free of other potential sources of bias |

Risk of bias table, Noweir 1984

| **Risk of bias domain** | **Bias in selection of participants into the study (or selection bias)** | **Performance bias** | **Bias due to exposure misclassification** | **Bias due to incomplete exposure data** | **Bias due to selective reporting of exposures** | **Bias due to conflicts of interest** | **Bias due to differences in numerator and denominator** | **Other biases** |
| --- | --- | --- | --- | --- | --- | --- | --- | --- |
| **Rating** | **PROBABLY HIGH** | **LOW** | **LOW** | **LOW** | **LOW** | **LOW** | **LOW** | **LOW** |
| **Justification for rating** | The study's eligibility criteria systematically excluded participants in the study sample such that exposure in the study sample may not represent exposure in the target population. | The exposure assessment was based on the environmental noise assessment and study personnel were adequately blinded or blinding was unlikely to influence exposure assessment, as described by the criteria for a rating of low risk of bias. | Direct evidence suggests that the method adopted in the study for the exposure assessment and assignment produces valid, accurate and reliable exposure data, ideally based on direct quantitative exposure measures. | There were no or minimal incomplete exposure data or any incomplete exposure data have been imputed using appropriate methods. | The average noise levels observed in the different departments of the three surveyed plants are presented in Table 1. These levels varied from 80.4 to 99.3 dBA. In plant B the noise levels varied considerably for those departments engaged in weaving preparation and spinning operations, necessitating two separate entries. | There is no evidence that the study conduct or reporting were influenced by a competing interest. | The groups in this study were categorized according to the exposure to noise level and there were no differences in numerator and denominator arising from a mismatch between the definitions. | I judge the study to be free of other potential sources of bias. |

Risk of bias table, Nyarubeli 2018

| **Risk of bias domain** | **Bias in selection of participants into the study (or selection bias)** | **Performance bias** | **Bias due to exposure misclassification** | **Bias due to incomplete exposure data** | **Bias due to selective reporting of exposures** | **Bias due to conflicts of interest** | **Bias due to differences in numerator and denominator** | **Other biases** |
| --- | --- | --- | --- | --- | --- | --- | --- | --- |
| **Rating** | **PROBABLY HIGH** | **PROBABLY LOW** | **LOW** | **PROBABLY LOW** | **PROBABLY LOW** | **LOW** | **PROBABLY HIGH** | **LOW** |
| **Justification for rating** | Although prior to taking noise measurements, the research team accompanied by a factory management representative has conducted a walk-through survey in each factory, with collected information about when the factory started steel production, available job groups/titles and other relevant details, some basic characteristics about workers were not evident, such as the proportion male/female and their respective ages. | Indirect evidence suggests that the exposure assessors and study personnel were adequately blinded. | The measurements seem were taken under apparently stable working conditions with the assumption that the measured result would be representative of the prevailing working situation. | It appears that all study participants exposed to noise were assessed (Table 3). | There is insufficient information about selective outcome reporting to permit a judgment of low risk of bias, but there is indirect evidence which suggests the study was free of selective reporting. | There is no evidence that the study conduct or reporting were influenced by a competing interest. | I judge the final number of participants that has been measured was too low in relation to the initial number. | I judge the study to be free of other potential sources of bias. |

Risk of bias table, Osibogun 2000

| **Risk of bias domain** | **Bias in selection of participants into the study (or selection bias)** | **Performance bias** | **Bias due to exposure misclassification** | **Bias due to incomplete exposure data** | **Bias due to selective reporting of exposures** | **Bias due to conflicts of interest** | **Bias due to differences in numerator and denominator** | **Other biases** |
| --- | --- | --- | --- | --- | --- | --- | --- | --- |
| **Rating** | **PROBABLY HIGH** | **LOW** | **LOW** | **LOW** | **PROBABLY LOW** | **LOW** | **LOW** | **LOW** |
| **Justification for rating** | The study's eligibility criteria systematically excluded participants in the study sample such that exposure in the study sample may not represent exposure in the target population. | The exposure assessment was based on the environmental noise assessment and study personnel were adequately blinded or blinding was unlikely to influence exposure assessment, as described by the criteria for a rating of low risk of bias. | Direct evidence suggests that the method adopted in the study for the exposure assessment and assignment produces valid, accurate and reliable exposure data, ideally based on direct quantitative exposure measures. | There were no or minimal incomplete exposure data or any incomplete exposure data have been imputed using appropriate methods. | There is insufficient information about selective exposure reporting to permit a judgment of low risk of bias, due to the fact that both right and left ears of the subjects were measure but only the air-conducting hearing thresholds for the right ear are presented in the current report. | There is no evidence that the study conduct or reporting were influenced by a competing interest. | The groups in this study were categorized according to the exposure to noise level and there were no differences in numerator and denominator arising from a mismatch between the definitions. | I judge the study to be free of other potential sources of bias. |

Risk of bias table, Pawlaczyk-Luszczynska 2016

| **Risk of bias domain** | **Bias in selection of participants into the study (or selection bias)** | **Performance bias** | **Bias due to exposure misclassification** | **Bias due to incomplete exposure data** | **Bias due to selective reporting of exposures** | **Bias due to conflicts of interest** | **Bias due to differences in numerator and denominator** | **Other biases** |
| --- | --- | --- | --- | --- | --- | --- | --- | --- |
| **Rating** | **LOW** | **LOW** | **LOW** | **LOW** | **LOW** | **LOW** | **LOW** | **LOW** |
| **Justification for rating** | TARGET POPULATION: blue collar workers from furniture factory STUDY POPULATION: 50 workers JUSTIFICATION: There is no information about selection. | Assessors haven’t prior knowledge of participant characteristics | No. Noise measurements and exposure evaluations at work stations in two departments of the furniture factory were performed using standardized method specified by PN-EN ISO 9612:2011 and PN-N-01307:1994.Additinally, the predicted noise reduction (PNR) of hearing protectors (HPs) worn by study subjects were estimated using the octave-band method according to PN-EN 458:2006. However, due to lack of data concerning actual usage of HPs, the efficiency of personal protective equipment were not taken into consideration in noise exposure evaluation. | Data were complete. Due to the rotation of employees, the energy average values of the daily noise exposure levels at various work stations in two analyzed departments of the furniture factory were the basis for estimating the risk of noise-induced hearing loss among study. | No, all exposures data were reported | Nobody from authors received support from entities with potential interest in the assessed exposure | Not applicable | No other problems could have introduced bias |

Risk of bias table, Rabinowitz 2007

| **Risk of bias domain** | **Bias in selection of participants into the study (or selection bias)** | **Performance bias** | **Bias due to exposure misclassification** | **Bias due to incomplete exposure data** | **Bias due to selective reporting of exposures** | **Bias due to conflicts of interest** | **Bias due to differences in numerator and denominator** | **Other biases** |
| --- | --- | --- | --- | --- | --- | --- | --- | --- |
| **Rating** | **LOW** | **HIGH** | **PROBABLY LOW** | **PROBABLY LOW** | **PROBABLY LOW** | **HIGH** | **LOW** | **PROBABLY HIGH** |
| **Justification for rating** | The study records provide sufficient detail for assessing risk of bias, including the: target population; study sample; criteria for eligibility for individuals to participate in the study; sampling, recruitment and enrolment procedures; rate of participation in the study; and rate of participation in the exposure assessment. The data reported in the study records are sufficiently detailed to support the conclusion that exposure in the study sample is representative of exposure in the target population, with similar characteristics reported for both the study sample and the target population. | Exposure assessors and study personnel were not blinded or were incompletely blinded, and the exposure assessment was likely to be influenced by the lack of blinding (e.g., exposure was systematically assessed differentially for sub-groups defined by participant characteristics). | Insufficient information exists about the exposure assessment and assignment method to permit you to rate the risk of bias as low. However, there is indirect evidence that the methods were valid, accurate and reliable, as described by the criteria for a rating of low risk of bias: Indirect evidence suggests that the exposure was assessed using validated methods that directly measure exposure. | There is insufficient information about the completeness of exposure data to permit a judgment of low risk of bias, but there is indirect evidence, which suggests incomplete exposure data were adequately addressed, as described by the criteria for a judgment of low risk of bias. | There is insufficient information about selective exposure reporting to permit a judgment of low risk of bias, but there is indirect evidence, which suggests the study was free of selective exposure reporting, as described by the criteria for a judgment of low risk of bias. | There is evidence that the study conduct or reporting were influenced by a competing interest. | I judge the study to be free of other potential sources of bias. | The authors did not consider the combined effects of noise. |

Risk of bias table, Rachiotis 2006

| **Risk of bias domain** | **Bias in selection of participants into the study (or selection bias)** | **Performance bias** | **Bias due to exposure misclassification** | **Bias due to incomplete exposure data** | **Bias due to selective reporting of exposures** | **Bias due to conflicts of interest** | **Bias due to differences in numerator and denominator** | **Other biases** |
| --- | --- | --- | --- | --- | --- | --- | --- | --- |
| **Rating** | **PROBABLY LOW** | **PROBABLY LOW** | **PROBABLY HIGH** | **PROBABLY LOW** | **PROBABLY HIGH** | **LOW** | **PROBABLY LOW** | **PROBABLY HIGH** |
| **Justification for rating** | Information about selection does not suffice to permit you to judge the risk of bias to be low. Indirect evidence (see Definitions) suggests that inclusion/exclusion criteria, recruitment and enrolment procedures, and participation/response rates were similar across groups as described by the criteria for a rating of low risk of bias. | The information on blinding is insufficient to permit a rating of low risk of bias. However, indirect evidence suggests that the exposure assessors and study personnel were adequately blinded or blinding was unlikely to influence exposure assessment, as described by the criteria for a rating of low risk of bias. | Insufficient information is provided about the exposure measurement method, including validity and reliability, but there is evidence for concern about the exposure measurement method used. | There is insufficient information about the completeness of exposure data to permit a judgment of low risk of bias, but there is indirect evidence, which suggests incomplete exposure data were adequately addressed, as described by the criteria for a judgment of low risk of bias. | There is insufficient information about selective exposure reporting to permit a judgment of high risk of bias, but there is indirect evidence, which suggests the study was not free of selective exposure reporting, as described by the criteria for a judgment of high risk of bias. | There is no evidence that the study conduct or reporting were influenced by a competing interest. | There is insufficient information to permit a rating of low risk of bias, but there is indirect evidence, which suggests the study was free of other potential sources of bias. | There is insufficient information to permit a rating of high risk of bias, but there is indirect evidence, which suggests the study was not free of other potential sources of bias, as described by the criteria for a rating of high risk of bias. |

Risk of bias table, Sancini 2014

| **Risk of bias domain** | **Bias in selection of participants into the study (or selection bias)** | **Performance bias** | **Bias due to exposure misclassification** | **Bias due to incomplete exposure data** | **Bias due to selective reporting of exposures** | **Bias due to conflicts of interest** | **Bias due to differences in numerator and denominator** | **Other biases** |
| --- | --- | --- | --- | --- | --- | --- | --- | --- |
| **Rating** | **PROBABLY HIGH** | **PROBABLY LOW** | **LOW** | **LOW** | **LOW** | **PROBABLY LOW** | **LOW** | **LOW** |
| **Justification for rating** | From 167 occupationally exposed to noise participants, only 72 were selected for final exposed group. | Table 2 shows exposure status for eligible participants with continuous equivalent level of noise. Data noise of exposed group were shown with interval values. | Evidence suggests that exposure was consistently assessed, using well-established methods that directly measure exposure: "In accordance with the legislation, the phonometric measurements in the departments were performed using a class 1 tool as defined in IEC (International Electrotechnical Commission)". | There were no or minimal incomplete exposure data or any incomplete exposure data have been imputed using appropriate methods. | There was a pre-published protocol, and the prevalence of all exposures and exposure categories has been reported as pre-specified in the protocol. | Although the sentence above indicates that all subjects agreed to the study, there was not a statement about interest conflicts. | The groups in this study were categorized according to the exposure to noise level and there were no differences in numerator and denominator arising from a mismatch between the definitions. | I judge the study to be free of other potential sources of bias |

Risk of bias table, Seixas 2001

| **Risk of bias domain** | **Bias in selection of participants into the study (or selection bias)** | **Performance bias** | **Bias due to exposure misclassification** | **Bias due to incomplete exposure data** | **Bias due to selective reporting of exposures** | **Bias due to conflicts of interest** | **Bias due to differences in numerator and denominator** | **Other biases** |
| --- | --- | --- | --- | --- | --- | --- | --- | --- |
| **Rating** | **HIGH** | **PROBABLY HIGH** | **PROBABLY LOW** | **PROBABLY LOW** | **PROBABLY LOW** | **PROBABLY HIGH** | **PROBABLY HIGH** | **LOW** |
| **Justification for rating** | The descriptions of the target population, inclusion/exclusion criteria, recruitment and enrolment procedures, participation/response rates and/or data on the distribution of relevant study sample and population characteristics suggest that the risk of selection effects was substantial. | The information on blinding is insufficient to permit a rating of low risk of bias. | Indirect evidence suggests that the exposure was consistently assessed using well-established methods that directly measure exposure. | Data on exposure status (or level) seem complete for eligible participants. | Noise monitoring was conducted with data-logging noise dosimeters. | Study authors did not make a claim denying conflicts of interest. Additionally, Kyle Ren, one of the authors is member of a refinery, which may indicate a conflict of interest about the results of study. | The authors did not specify the proportion of persons invited to participate in the study, in relation of participants who did participate in the study. | I judge the study to be free of other potential sources of bias. |

Risk of bias table, Shakhatreh 2000

| **Risk of bias domain** | **Bias in selection of participants into the study (or selection bias)** | **Performance bias** | **Bias due to exposure misclassification** | **Bias due to incomplete exposure data** | **Bias due to selective reporting of exposures** | **Bias due to conflicts of interest** | **Bias due to differences in numerator and denominator** | **Other biases** |
| --- | --- | --- | --- | --- | --- | --- | --- | --- |
| **Rating** | **PROBABLY HIGH** | **LOW** | **LOW** | **LOW** | **LOW** | **PROBABLY LOW** | **LOW** | **LOW** |
| **Justification for rating** | The study's eligibility criteria systematically excluded participants in the study sample such that exposure in the study sample may not represent exposure in the target population | The exposure assessment was based on the environmental noise assessment and study personnel were adequately blinded or blinding was unlikely to influence exposure assessment, as described by the criteria for a rating of low risk of bias. | Direct evidence suggests that the method adopted in the study for the exposure assessment and assignment produces valid, accurate and reliable exposure data, ideally based on direct quantitative exposure measures. | There were no or minimal incomplete exposure data or any incomplete exposure data have been imputed using appropriate methods. | The prevalence rate of hearing loss was higher among the exposed group i.e. 30% in the exposed group and 8% in the non-exposed group. Hearing loss increased with increasing level of noise reaching 73% in the 95dB(A) area. Average hearing loss was highest amongst those who were employed for 25 years or more, reaching 39% dB(HL). | There is no evidence that the study conduct or reporting were influenced by a competing interest. | The groups in this study were categorized according to the exposure to noise level and there were no differences in numerator and denominator arising from a mismatch between the definitions. | I judge the study to be free of other potential sources of bias. |

Risk of bias table, Shi 2009

| **Risk of bias domain** | **Bias in selection of participants into the study (or selection bias)** | **Performance bias** | **Bias due to exposure misclassification** | **Bias due to incomplete exposure data** | **Bias due to selective reporting of exposures** | **Bias due to conflicts of interest** | **Bias due to differences in numerator and denominator** | **Other biases** |
| --- | --- | --- | --- | --- | --- | --- | --- | --- |
| **Rating** | **LOW** | **LOW** | **PROBABLY HIGH** | **LOW** | **LOW** | **LOW** | **LOW** | **LOW** |
| **Justification for rating** | The study selected All the workers who had conducted the body examination in the hospital from March to April in 2008. As described in the Regulation of Occupational Health Examination in China all the workers have to take the occupational health examination each year, so the study population could reflect the target population because usually all the workers should take the examination within a short time in order to not disturb their regular work. Therefore, the risk of selection bias can be assessed as low. | The exposed and unexposed workers were selected according the measurement of the noise level in the workplace. So the selection of the exposure group is quite accurate. | The method adopted in the study for the exposure assessment is based on environmental exposure data, therefore the risk in this domain is probably high | There were minimal incomplete exposure data have been imputed using appropriate methods because the study had evaluated the noise exposure level in most job position. | There is no protocol reported in the article, however the exposure status of noise was defined by the accurate detection of noise level. Therefore, the measurement of the noise level in the job position which suggests the study was free of selective exposure reporting, as described by the criteria for a judgment of low risk of bias. | The author was working in the CDC in Zhongyuan oil field in Puyuan City (Author information), and the oil field was managed by government. Because the regulation of the government and the CDC, the risk of potential interest is low. | The numerator defined as the number of workers who exposed the noise above the 85db(A). The measurement of the noise level in the job position can accurate help to define the worker who is exposed with noise. | I judge the study to be free of other potential sources of bias. |

Risk of bias table, Souza 2001

| **Risk of bias domain** | **Bias in selection of participants into the study (or selection bias)** | **Performance bias** | **Bias due to exposure misclassification** | **Bias due to incomplete exposure data** | **Bias due to selective reporting of exposures** | **Bias due to conflicts of interest** | **Bias due to differences in numerator and denominator** | **Other biases** |
| --- | --- | --- | --- | --- | --- | --- | --- | --- |
| **Rating** | **PROBABLY LOW** | **PROBABLY LOW** | **PROBABLY LOW** | **PROBABLY LOW** | **PROBABLY LOW** | **PROBABLY LOW** | **PROBABLY LOW** | **LOW** |
| **Justification for rating** | The proportion of persons invited to participate in the study who did participate in the study was acceptable. | The information on blinding is insufficient to permit a rating of low risk of bias. However, indirect evidence suggests that the exposure assessors and study personnel were adequately blinded. | Indirect evidence suggests that the exposure was consistently assessed using well-established methods that directly measure exposure. | Data on exposure level (percentage dose) are shown for ambient noise levels and occupation. | It seems data were collected directly from the participants (dosimetry reported). | There is insufficient information to permit a rating of low risk of bias, but there is indirect evidence which suggests the study was free of support from a company, study author, or other entity having a financial interest in the outcome of the study. | The numerator and denominator for the prevalence estimate seem appropriate. | I judge the study to be free of other potential sources of bias. |

Risk of bias table, Sriopas 2017

| **Risk of bias domain** | **Bias in selection of participants into the study (or selection bias)** | **Performance bias** | **Bias due to exposure misclassification** | **Bias due to incomplete exposure data** | **Bias due to selective reporting of exposures** | **Bias due to conflicts of interest** | **Bias due to differences in numerator and denominator** | **Other biases** |
| --- | --- | --- | --- | --- | --- | --- | --- | --- |
| **Rating** | **LOW** | **LOW** | **LOW** | **LOW** | **PROBABLY HIGH** | **LOW** | **LOW** | **PROBABLY LOW** |
| **Justification for rating** | The study records provide sufficient detail for assessing risk of bias, including the: target population; study sample; criteria for eligibility for individuals to participate in the study; sampling, recruitment and enrolment procedures; rate of participation in the study; and rate of participation in the exposure assessment. The data reported in the study records are sufficiently detailed to support the conclusion that exposure in the study sample is representative of exposure in the target population, with similar characteristics reported for both the study sample and the target population. | Exposure assessors and study personnel were blinded to relevant participant characteristics, and the blinding was probably not broken. | *Direct* evidence suggests that the method adopted in the study for the exposure assessment and assignment produces valid, accurate and reliable exposure data, ideally based on direct quantitative exposure measures. | There were no or minimal incomplete exposure data or any incomplete exposure data have been imputed using appropriate methods. | There is insufficient information about selective exposure reporting to permit a judgment of high risk of bias, but there is indirect evidence, which suggests the study was not free of selective exposure reporting, as described by the criteria for a judgment of high risk of bias. | There is no evidence that the study conduct or reporting were influenced by a competing interest. | I judge the study to be free of other potential sources of bias. | There is insufficient information to permit a rating of low risk of bias, but there is indirect evidence, which suggests the study was free of other potential sources of bias. |

Risk of bias table, Starck 1999

| **Risk of bias domain** | **Bias in selection of participants into the study (or selection bias)** | **Performance bias** | **Bias due to exposure misclassification** | **Bias due to incomplete exposure data** | **Bias due to selective reporting of exposures** | **Bias due to conflicts of interest** | **Bias due to differences in numerator and denominator** | **Other biases** |
| --- | --- | --- | --- | --- | --- | --- | --- | --- |
| **Rating** | **PROBABLY HIGH** | **PROBABLY LOW** | **PROBABLY LOW** | **PROBABLY LOW** | **PROBABLY LOW** | **PROBABLY LOW** | **PROBABLY LOW** | **PROBABLY HIGH** |
| **Justification for rating** | There is insufficient information about participant selection such as inclusion/exclusion criteria, recruitment and enrolment procedures. | Indirect evidence suggests that the exposure assessors and study personnel were adequately blinded. | There is insufficient information about the exposure measurement methods to permit a rating of low risk of bias. However, there is indirect evidence that exposure measurement methods were accurate. | Data on exposure level are shown for all participants. | It seems data were collected directly from the participants (dosimetry reported). | There is insufficient information to permit a rating of low risk of bias, but there is indirect evidence which suggests the study was free of support from a company, study author, or other entity having a financial interest in the outcome of the study. | The numerator and denominator for the prevalence estimate seem appropriate. | There was not a reference group for noise exposure. All workers were exposed to high level noise. |

Risk of bias table, Stokholm 2013

| **Risk of bias domain** | **Bias in selection of participants into the study (or selection bias)** | **Performance bias** | **Bias due to exposure misclassification** | **Bias due to incomplete exposure data** | **Bias due to selective reporting of exposures** | **Bias due to conflicts of interest** | **Bias due to differences in numerator and denominator** | **Other biases** |
| --- | --- | --- | --- | --- | --- | --- | --- | --- |
| **Rating** | **LOW** | **PROBABLY LOW** | **PROBABLY LOW** | **PROBABLY HIGH** | **PROBABLY LOW** | **PROBABLY LOW** | **PROBABLY LOW** | **PROBABLY HIGH** |
| **Justification for rating** | The descriptions of the target population, inclusion/exclusion criteria, recruitment and enrolment procedures (including sampling frame), participation/ response rates are sufficiently detailed, and adequate data were supplied on the distribution of relevant study sample and population characteristics. | Indirect evidence suggests that the exposure assessors and study personnel were adequately blinded. | It seems that the same data collection mode was used for measuring exposure among all study participants | Although the elevated number of participants, data on exposure level are not shown for all participants, as can be seen in table 3. | It seems data were collected directly from the participants (dosimetry reported). | There is indirect evidence which suggests the study was free of support from a company, study author, or other entity having a financial interest in the outcome of the study. | The difference between initial and final number of subjects seems appropriate (66%), almost 70%. | The author declared that individual information on the use of hearing protection devices was not available, and thus reduce the power to detect an effect. |

Risk of bias table, Strauss 2014

| **Risk of bias domain** | **Bias in selection of participants into the study (or selection bias)** | **Performance bias** | **Bias due to exposure misclassification** | **Bias due to incomplete exposure data** | **Bias due to selective reporting of exposures** | **Bias due to conflicts of interest** | **Bias due to differences in numerator and denominator** | **Other biases** |
| --- | --- | --- | --- | --- | --- | --- | --- | --- |
| **Rating** | **PROBABLY HIGH** | **HIGH** | **PROBABLY LOW** | **PROBABLY HIGH** | **PROBABLY HIGH** | **LOW** | **HIGH** | **LOW** |
| **Justification for rating** | Information about selection does not suffice to permit you to judge the risk of bias to be high. Indirect evidence suggests that inclusion/exclusion criteria, recruitment and enrolment procedures, and participation/response rates differed across groups, as described by the criteria for a rating of high risk of bias. | Exposure assessors and study personnel were not blinded or were incompletely blinded, and the exposure assessment was likely to be influenced by the lack of blinding (e.g., exposure was systematically assessed differentially for sub-groups defined by participant characteristics). | Insufficient information exists about the exposure assessment and assignment method to permit you to rate the risk of bias as low. However, there is indirect evidence that the methods were valid, accurate and reliable, as described by the criteria for a rating of low risk of bias. Indirect evidence suggests that the exposure was assessed using validated methods that directly measure exposure. | There is insufficient information about incomplete exposure data to permit a judgment of high risk of bias, but there is indirect evidence that incomplete exposure data were not adequately addressed, as described by the criteria for a judgment of high risk of bias. | There is insufficient information about selective exposure reporting to permit a judgment of high risk of bias, but there is indirect evidence which suggests the study was not free of selective exposure reporting, as described by the criteria for a judgment of high risk of bias. | There is no evidence that the study conduct or reporting were influenced by a competing interest. | There is insufficient information to permit a rating of high risk of bias, but there is indirect evidence, which suggests the study was not free of other potential sources of bias, as described by the criteria for a rating of high risk of bias. | I judge the study to be free of other potential sources of bias. |

Risk of bias table, Talbott 1999

| **Risk of bias domain** | **Bias in selection of participants into the study (or selection bias)** | **Performance bias** | **Bias due to exposure misclassification** | **Bias due to incomplete exposure data** | **Bias due to selective reporting of exposures** | **Bias due to conflicts of interest** | **Bias due to differences in numerator and denominator** | **Other biases** |
| --- | --- | --- | --- | --- | --- | --- | --- | --- |
| **Rating** | **PROBABLY HIGH** | **PROBABLY HIGH** | **PROBABLY HIGH** | **PROBABLY LOW** | **PROBABLY LOW** | **HIGH** | **PROBABLY LOW** | **LOW** |
| **Justification for rating** | Participant inclusion and exclusion criteria were not appropriately defined. | The information on blinding is insufficient to permit a rating of high risk of bias. However, indirect evidence suggests that the exposure assessors and study personnel were not adequately blinded | There is evidence that the exposure was assessed using indirect measures that have not been validated or empirically shown to be consistent with methods that directly measure exposure | Although exposure was assessed using indirect measures, both study samples had the same response rate of approximately 70%. | The exposure assessment was based on individual exposure measurements. | The study received support from a company. | The numerator and denominator were appropriate for the prevalence estimate. | I judge the study to be free of other potential sources of bias. |

Risk of bias table, Toppila 2001

| **Risk of bias domain** | **Bias in selection of participants into the study (or selection bias)** | **Performance bias** | **Bias due to exposure misclassification** | **Bias due to incomplete exposure data** | **Bias due to selective reporting of exposures** | **Bias due to conflicts of interest** | **Bias due to differences in numerator and denominator** | **Other biases** |
| --- | --- | --- | --- | --- | --- | --- | --- | --- |
| **Rating** | **PROBABLY LOW** | **LOW** | **LOW** | **LOW** | **LOW** | **PROBABLY LOW** | **LOW** | **LOW** |
| **Justification for rating** | All males workers in these workplaces where included in the data collection. | The exposure assessment was based on the environmental noise assessment and study personnel were adequately blinded or blinding was unlikely to influence exposure assessment, as described by the criteria for a rating of low risk of bias. | Direct evidence suggests that the method adopted in the study for the exposure assessment and assignment produces valid, accurate and reliable exposure data, ideally based on direct quantitative exposure measures. | There were no or minimal incomplete exposure data or any incomplete exposure data have been imputed using appropriate methods. | The test proceeded from 1 kHz to lower frequencies (500 and 250 Hz), then back to 1 kHz and up to higher frequencies (2, 4 and 8 kHz). The test was repeated if the two measurements at 1 kHz yielded a discrepancy greater than 5 dB. The mean heating level of the right and left ear at 4 kHz was used in most statistical calculations. | There is no evidence that the study conduct or reporting were influenced by a competing interest. | The groups in this study were categorized according to the exposure to noise level and there were no differences in numerator and denominator arising from a mismatch between the definitions. | I judge the study to be free of other potential sources of bias. |

Risk of bias table, Vihma 1981

| **Risk of bias domain** | **Bias in selection of participants into the study (or selection bias)** | **Performance bias** | **Bias due to exposure misclassification** | **Bias due to incomplete exposure data** | **Bias due to selective reporting of exposures** | **Bias due to conflicts of interest** | **Bias due to differences in numerator and denominator** | **Other biases** |
| --- | --- | --- | --- | --- | --- | --- | --- | --- |
| **Rating** | **PROBABLY LOW** | **PROBABLY LOW** | **PROBABLY LOW** | **PROBABLY LOW** | **PROBABLY HIGH** | **PROBABLY LOW** | **PROBABLY LOW** | **LOW** |
| **Justification for rating** | The study sample seems an adequate representation of the target population. | Indirect evidence suggests that the exposure assessors and study personnel were adequately blinded. | Indirect evidence suggests that the exposures were consistently assessed. | There was mention about the exposure results for all eligible participants. | The exposure assessment was not based on individual exposure measurements (e.g. personal dosimetry). | There is no evidence which suggests the study was supported from a company, study author, or other entity having a financial interest in the outcome of the study. | The numerator and denominator for the prevalence estimate were appropriate | I judge the study to be free of other potential sources of bias |

Risk of bias table, Virkkunen 2005

| **Risk of bias domain** | **Bias in selection of participants into the study (or selection bias)** | **Performance bias** | **Bias due to exposure misclassification** | **Bias due to incomplete exposure data** | **Bias due to selective reporting of exposures** | **Bias due to conflicts of interest** | **Bias due to differences in numerator and denominator** | **Other biases** |
| --- | --- | --- | --- | --- | --- | --- | --- | --- |
| **Rating** | **LOW** | **PROBABLY LOW** | **PROBABLY LOW** | **PROBABLY LOW** | **PROBABLY LOW** | **PROBABLY LOW** | **PROBABLY LOW** | **PROBABLY HIGH** |
| **Justification for rating** | The descriptions of the target population, inclusion/exclusion criteria, recruitment and enrolment procedures (including sampling frame), participation/ response rates are sufficiently detailed, and adequate data were supplied on the distribution of relevant study sample and population characteristics. | Indirect evidence suggests that the exposure assessors and study personnel were adequately blinded. | Exposure was assessed using indirect measures ("exposure is characterized by the proportion of exposed (prevalence P) and the mean level of exposure among the exposed (level L) by occupation and period") that have been validated or empirically shown to be consistent with methods that directly measure exposure. | Data on exposure level are shown for all participants on the three follow-up periods, as can be seen in tables 1 and 3. | Although the noise was not directly measured, there is indirect evidence that exposure measurement methods were accurate. | There is indirect evidence which suggests the study was free of support from a company, study author, or other entity having a financial interest in the outcome of the study. | Considering there was no loss of participants on the analyses, numerator and denominator for the prevalence estimate seem appropriate. | The authors declare the inclusion of white-collar analyses would impact the results. |

Risk of bias table, Whittaker 2014

| **Risk of bias domain** | **Bias in selection of participants into the study (or selection bias)** | **Performance bias** | **Bias due to exposure misclassification** | **Bias due to incomplete exposure data** | **Bias due to selective reporting of exposures** | **Bias due to conflicts of interest** | **Bias due to differences in numerator and denominator** | **Other biases** |
| --- | --- | --- | --- | --- | --- | --- | --- | --- |
| **Rating** | **PROBABLY HIGH** | **PROBABLY HIGH** | **PROBABLY LOW** | **PROBABLY LOW** | **PROBABLY HIGH** | **PROBABLY LOW** | **PROBABLY LOW** | **PROBABLY LOW** |
| **Justification for rating** | The authors declared they included some participants for feasibility. | Indirect evidence suggests that the exposure assessors and study personnel were not adequately blinded. | Indirect evidence suggests that the exposure was consistently assessed using well-established methods that directly measure exposure. | There was a loss of 13 individuals for analyses, and it was adequately justified by authors. | The noise was not directly measured. | There is indirect evidence which suggests the study was free of support from a company, study author, or other entity having a financial interest in the outcome of the study. | In total, 77% of participants were lost, which didn't seem to have impacted numerator and denominator for the prevalence estimate. | There is insufficient information to permit a rating of low risk of bias, but there is indirect evidence, which suggests the study was free of other potential sources of bias. |

Risk of bias table, Wu 1987

| **Risk of bias domain** | **Bias in selection of participants into the study (or selection bias)** | **Performance bias** | **Bias due to exposure misclassification** | **Bias due to incomplete exposure data** | **Bias due to selective reporting of exposures** | **Bias due to conflicts of interest** | **Bias due to differences in numerator and denominator** | **Other biases** |
| --- | --- | --- | --- | --- | --- | --- | --- | --- |
| **Rating** | **PROBABLY HIGH** | **LOW** | **LOW** | **PROBABLY LOW** | **PROBABLY HIGH** | **PROBABLY LOW** | **LOW** | **LOW** |
| **Justification for rating** | In total. 73% of the screened population were study participants which didn't seem to have impacted numerator and denominator for the prevalence estimate. | Indirect evidence suggests that the exposure assessors and study personnel were adequately blinded. | Direct evidence suggest that the method adopted in the study for the exposure assessment and assignment produces valid, accurate and reliable exposure data. | A random-matching computer program was used to select randomly one control from all those who met the matching criteria for each exposed subject. | The exposure assessment was not based on individual exposure measurements (e.g. personal dosimetry). Previous documentation concerning noise exposure intensity of these workers was unavailable, which may influence the effect of exposure. | There is indirect evidence which suggests the study was free of support from a company, study author, or other entity having a financial interest in the outcome of the study. | The groups in this study were categorized according to the exposure to noise level and there were no differences in numerator and denominator arising from a mismatch between the definitions. | I judge the study to be free of other potential sources of bias. |

Risk of bias table, Xiao 2008

| **Risk of bias domain** | **Bias in selection of participants into the study (or selection bias)** | **Performance bias** | **Bias due to exposure misclassification** | **Bias due to incomplete exposure data** | **Bias due to selective reporting of exposures** | **Bias due to conflicts of interest** | **Bias due to differences in numerator and denominator** | **Other biases** |
| --- | --- | --- | --- | --- | --- | --- | --- | --- |
| **Rating** | **PROBABLY HIGH** | **PROBABLY LOW** | **PROBABLY HIGH** | **LOW** | **LOW** | **LOW** | **Probably low** | **LOW** |
| **Justification for rating** | The study selected all the workers exposed noise into the exposure group and randomly selected 953 workers who are not exposed to noise into the control group. Therefore, the number of workers who are not exposed to noise had been underestimated. | All participants were selected by researcher according to their job title, but the lack of blinding is unlikely to have influenced the exposure assessment. | This paper only briefly describes the noise intensity of the work place of textile, stamping, grinding and crushing work is 85 ~ 110 dB (A), and the average value is (93.2 ± 9.1) dB (A). However, it does not describe how many points are measured, nor does it perform individual noise detection. Therefore, the methods used for assessing exposure of noise might under-estimated the exposure. | There were no or minimal incomplete exposure data or any incomplete exposure data have been imputed using appropriate methods. | The article does not include a pre-plan, but reports on all types of exposure and exposure in the article. | The author is working in Guangdong Provincial Occupational Disease Prevention and Treatment Institute.It is a technical institution under the government department. Because the regulation of the government, the risk of potential interest is low. | The numerator is defined as the number of workers who exposed to noise. The denominator is defined as the total number exposed to noise. | I judge the study to be free of other potential sources of bias. |

Risk of bias table, Xie 2015

| **Risk of bias domain** | **Bias in selection of participants into the study (or selection bias)** | **Performance bias** | **Bias due to exposure misclassification** | **Bias due to incomplete exposure data** | **Bias due to selective reporting of exposures** | **Bias due to conflicts of interest** | **Bias due to differences in numerator and denominator** | **Other biases** |
| --- | --- | --- | --- | --- | --- | --- | --- | --- |
| **Rating** | **HIGH** | **LOW** | **LOW** | **LOW** | **LOW** | **LOW** | **LOW** | **LOW** |
| **Justification for rating** | The study only selected workers who exposed to noise, and there is no information about total number of workers in the factories. Therefore, the recruitment and enrolment procedures is not representative of the target population. | All participants were selected by researcher according to their job title and their working environment, and were assumed that they are all exposed to noise by researcher. However, Table 1 shows that all the individual noise measurement are above 85 dBA. Therefore, the adequate blinding was unlikely to influence exposure assessment. | Direct evidence suggests that the method adopted in the study for the exposure assessment and assignment produces valid, accurate and reliable exposure data, ideally based on direct quantitative exposure measures (The measurement of continuous personal noise exposure) | There were no or minimal incomplete exposure data or any incomplete exposure data have been imputed using appropriate methods (all data reported in Table 1). | All categorical noise exposure level are reported in Table 1. | The author declares that there is no actual or potential conflict of interest in this article. | The groups in this study were categorized according to the exposure to noise. The personal noise was detected which also prove there were no differences in numerator and denominator arising from a mismatch between the definitions. | I judge the study to be free of other potential sources of bias. |

Risk of bias table, Yu 2017

| **Risk of bias domain** | **Bias in selection of participants into the study (or selection bias)** | **Performance bias** | **Bias due to exposure misclassification** | **Bias due to incomplete exposure data** | **Bias due to selective reporting of exposures** | **Bias due to conflicts of interest** | **Bias due to differences in numerator and denominator** | **Other biases** |
| --- | --- | --- | --- | --- | --- | --- | --- | --- |
| **Rating** | **LOW** | **PROBABLY LOW** | **LOW** | **LOW** | **LOW** | **LOW** | **LOW** | **LOW** |
| **Justification for rating** | Although the study only recruited workers who exposed to noise above 80 dBa, the study also presents a total number of workers in the company. Therefore, exposure in the study sample is representative of exposure in the target population | All workers in the cohort were selected by researcher according to the environmental noise level they exposed. | The exposure assessment was based on individual noise measurement. | There were no or minimal incomplete exposure data, or any incomplete exposure data have been imputed using appropriate methods (all data reported in Table 1). | All categorical noise exposure level are reported in Table 1 | The study was approved by the Ethics Committee of the Henan Provincial Institute of Occupational Disease Prevention and Control (batch number: 2013003). Informed consent was signed by all respondents. | The numerator is workers exposed to noise above 80dBA and denominator is the total number of workers in the company. The personal noise was detected, which also proves there were no differences in numerator and denominator arising from a mismatch between the definitions. | I judge the study to be free of other potential sources of bias |

**Sheet of all prevalence estimates from included studies**

| **Study ID** | **Country** | **Region** | **ISIC code** | **Industrial sector** | **ISCO code** | **Occupation** | **Sex** | **Age** | **N_workers** | **N_exposed** | **N_unexposed** |
| --- | --- | --- | --- | --- | --- | --- | --- | --- | --- | --- | --- |
| Ahmed 2001 | Saudi Arabia | EMRO | 24, 35 | Many | 8121, 7127 | Many | Male | Mean | 368 | 202 | 99 |
| Attarchi 2012 | Iran, Islamic Republic of | EMRO |  |  |  |  | Male | Mean | 331 | 167 | 164 |
| Attarchi 2013 | Iran, Islamic Republic of | EMRO | 29 | B-Mining and quarrying | 3341, 7131, 8211 | Many | Male | Mean | 471 | 347 | 124 |
| Bauer 1991 | Austria | EURO |  |  |  |  | Both | 5 categories | 47388 | 45154 | 2234 |
| Cantley 2015 | USA | AMRO | 32 | B-Mining and quarrying | 7223 | 7-Craft and Related Trades Workers | Both | Mean | 9220 | 2422 | 6798 |
| Chang - 2003 | Taiwan | WPRO | 45 | G-Waste collection, treatment and disposal activities; materials recovery | 1290 | 1-Managers | Male | Mean | 20 | 15 | 5 |
| Chang 2009 | Taiwan | WPRO | 15 | B-Mining and quarrying | 7543, 8159, 7322, 9611, 4110, 9329 | Many | Both | Mean | 59 | 42 | 17 |
| Chang 2012 | Taiwan | WPRO | 32 | B-Mining and quarrying | 8121 | 8-Plant and Machine Operators and Assemblers | Both | Mean | 281 | 68 | 120 |
| Chen 2017 | China | WPRO | 32 | B-Mining and quarrying | 8189 | 8-Plant and Machine Operators and Assemblers | Male | Mean | 1390 | 900 | 490 |
| de Souza 2015 | Brazil | AMRO | 6 | B-Mining and quarrying | 3134 | 3-Technicians and Associate Professionals | Both | Mean | 1.729 | 470 | 1259 |
| Du 2007 | China | WPRO | 13 Manufacture of textiles | B-Mining and quarrying | 8152 Weaving and knitting machine operators | 8-Plant and Machine Operators and Assemblers | Male | Mean | 3279 | 1342 | 1937 |
| Guo 2012 | China | WPRO | 13 Manufacture of textiles | B-Mining and quarrying | 8159 Textile products machine operators | 8-Plant and Machine Operators and Assemblers | Both | Mean | 60 | 60 |  |
| He 2017 | China | WPRO | 20 | B-Mining and quarrying | 91 overhaulers, whose 8-hour equivalent sound level is 86.23 dB (A); 133 laboratory workers, whose 8-hour equivalent sound level is 67.57 dB (A); 123 other ordinary operators, whose 8-hour equivalent sound level is 80.6 dB (A); 74 public works operators, whose 8-hour equivalent sound level is 90.65 dB (A) 。 | Many | Both | Mean | 513 | 165 | 348 |
| Hong 1998 | South Korea | WPRO | 51 | H-Transportation and storage | 3153 | 3-Technicians and Associate Professionals | Male | Mean | 450 | 255 | 195 |
| Hu 2005 | China | WPRO | 24 | B-Mining and quarrying | 8121, 3343 | many |  | Mean | 191 | 123 | 68 |
| Hughes 2013 | USA | AMRO | 51 | H-Transportation and storage | 3153 | 3-Technicians and Associate Professionals | Both | 5 categories | 503 | 368 | 135 |
| Inoue 2005 | Japan | WPRO | 17 | B-Mining and quarrying | 8171 | 8-Plant and Machine Operators and Assemblers | Male | Mean | 415 | 242 | 173 |
| Ivanovich 1994 | Bulgaria | EURO | 61 | J-Information and communication | 2656 | 2-Professionals | Female | Mean | 249 | 81 | 168 |
| Johnson 2006 | Sweden | EURO | 96 | B-Mining and quarrying | 7315 | 7-Craft and Related Trades Workers | Both | Mean | 313 | 146 | 167 |
| Kock 2004 | Denmark | EURO | 32 | B-Mining and quarrying |  |  |  | Mean | 741 | 254 | 487 |
| Kovacevic 2006 | Montenegro | EURO | 13 | B-Mining and quarrying | 8152 | 8-Plant and Machine Operators and Assemblers | Both | Mean | 225 | 111 | 114 |
| Landen 2004 | USA | AMRO | 8 | B-Mining and quarrying | 8111 | 8-Plant and Machine Operators and Assemblers | Both | 5 categories | 309 | 213 | 96 |
| Lee 1999 | Singapore | WPRO | 96 | S-Other service activities | 9626 | 9-Elementary Occupations | Both | Mean | 80 | 40 | 37 |
| Lee 2009 | South Korea | WPRO | 24 | B-Mining and quarrying |  |  | Male | Mean | 530 | 133 | 397 |
| Liu 2015 | China | WPRO | 32 | B-Mining and quarrying | 8121 | 8-Plant and Machine Operators and Assemblers | Both | Mean | 247 | 100 | 147 |
| Lv 2003 | China | WPRO |  | Section H - Transportation and storage | 7232Aircraft engine mechanics and repairers | 7-Craft and Related Trades Workers | Male | Mean | 319 | 290 | 29 |
| Maccà 2014 | Italy | EURO | 13, 25, 32, 41 | Many | 8159, 7211, 9313, 8114, | Many | Both | Mean | 285 | 137 | 148 |
| Noweir 1984 | Egypt | EMRO | 13 | B-Mining and quarrying | 7318 | 7-Craft and Related Trades Workers | Male | 5 categories | 2458 | 1404 | 1054 |
| Melamed 1997 | Israel | EURO | 10 ; 13 ; 24; 26 | B-Mining and quarrying | 8121; 8159; 8189; 8212; 8160 | Many | Both | Mean | 970 | 205 | 765 |
| Morata 1997 | Brazil | AMRO | 32 | B-Mining and quarrying | 3139 | 3-Technicians and Associate Professionals | Male | Mean | 124 | 74 | 50 |
| Morata 1997 | USA | AMRO | 6 | B-Mining and quarrying | 3134 | 3-Technicians and Associate Professionals |  | mean | 438 | 189 | 249 |
| Nasir 2012 | Malaysia | WPRO | 51 | H-Transportation and storage | 3153 | 3-Technicians and Associate Professionals | Both | mean | 358 | 136 | 222 |
| NHIS 2014 | USA | AMRO | General working population | General working population | General working population | General working population | Both | Mean, 15 categories | 34045 | 3543 | 30502 |
| NHIS 2007 | USA | AMRO | General working population | General working population | General working population | General working population | Both | Mean, 15 categories | 20800 | 2094 | 18706 |
| NHANES 1999-2004 | USA | AMRO | General working population | General working population | General working population | General working population | Both | Mean, 15 categories | 9721 | 1523 | 8198 |
| Nyarubeli 2018 | Tanzania | AFRO | 25 | B-Mining and quarrying | 7211, 7223 | many | Unclear | - | 326 | 293 | 33 |
| Osibogun 2000 | Nigeria | AFRO | 13 | B-Mining and quarrying | 8159 | 8-Plant and Machine Operators and Assemblers | Both | 4 categories | 204 | 116 | 71 |
| Parent-Thirion 2017 (EWCS) | 35 European countries: Albania, Austria, Belgium, Bulgaria, Croatia, Cyprus, Czech Republic, Denmark, Estonia, Finland, France, FYROM, Germany, Greece, Hungary, Ireland, Italy, Latvia, Lithuania, Luxembourg, Malta, Montenegro, Netherlands, Norway, Poland, Portugal, Romania, Serbia, Slovakia, Slovenia, Spain, Sweden, Switzerland, Turkey, United Kingdom | EURO | General working population | General working population | General working population | General working population | Both | Categories | 43636 | 7831 | 35805 |
| Pawlaczyk-Luszczynska 2016 | POLAND | EURO | 31 | B-Mining and quarrying | 8189 | 8-Plant and Machine Operators and Assemblers | Male | mean | 50 | 50 |  |
| Rabinowitz 2007 | USA | AMRO | 32 | B-Mining and quarrying | 7223 | 7-Craft and Related Trades Workers | Male | mean | 6217 | 1759 | 4458 |
| Rachiotis 2006 | GREECE | EURO | 26 | B-Mining and quarrying | 8212 | 8-Plant and Machine Operators and Assemblers | both | mean | 145 | 94 | 51 |
| Sancini 2014 | Italy | EURO | 17 | B-Mining and quarrying | 8171 | 8-Plant and Machine Operators and Assemblers | Male | mean | 191 | 72 | 119 |
| Seixas 2001 | USA | AMRO | 43 | F-Construction | 7411 | 7-Craft and Related Trades Workers |  | mean | 59 | 40 | 19 |
| Shakhatreh 2000 | JORDAN | EMRO | 32 | B-Mining and quarrying | 8159 | 8-Plant and Machine Operators and Assemblers | both | mean | 140 | 22 | 70 |
| Singh 2012 | INDIA | SEARO | 24 | B-Mining and quarrying | 7221 | 7-Craft and Related Trades Workers |  | mean | 222 | 165 | 57 |
| Sliwinska-Kowalska 2004 | POLAND | EURO | 3 | A-Agriculture, forestry and fishing | 8350 | 8-Plant and Machine Operators and Assemblers | both | mean | 906 | 701 | 205 |
| Shi 2009 | China | WPRO | 06 Extraction of crude petroleum and natural gas | B-Mining and quarrying |  |  | Male | mean | 1170 | 385 | 785 |
| Solecki 2008 | POLAND | EURO | 1 | A-Agriculture, forestry and fishing | 6111 | 6-Skilled Agricultural, Forestry and Fishery Workers |  | mean | 44 | 44 |  |
| Souza 2001 | Brazil | AMRO | 6 | B-Mining and quarrying | 3134 | 3-Technicians and Associate Professionals | Male | mean | 775 | 585 | 190 |
| Sriopas 2017 | Thailand | SEARO | 45 | G-Waste collection, treatment and disposal activities; materials recovery | 7212 | 7-Craft and Related Trades Workers | Male | mean | 180 | 113 | 67 |
| Starck 1999 |  |  | 43 | F-Construction | 6210 | 6-Skilled Agricultural, Forestry and Fishery Workers |  | mean | 370 | 46 | 324 |
| Stokholm 2013 | Denmark | EURO |  |  |  |  | both | mean | 11395 | 6262 | 5133 |
| Strauss 2014 | South Africa | AFRO | 7 | B-Mining and quarrying | 8111 | 8-Plant and Machine Operators and Assemblers | Male | 5 categories | 40123 | 33961 | 6,162 |
| Talbott 1999 | USA | AMRO | 29 | B-Mining and quarrying | 8211 | 8-Plant and Machine Operators and Assemblers | Male | mean | 308 | 62 | 246 |
| Toppila 2001 | FINLAND | EURO |  |  |  |  | Male | mean | 706 | 706 |  |
| Vihma 1981 | FINLAND | EURO | 16, 22, 24, 27 | B-Mining and quarrying | 8121 | 8-Plant and Machine Operators and Assemblers | both | mean | 1181 | 404 | 777 |
| Virkkunen 2005 | FINLAND | EURO | 96 | B-Mining and quarrying | 8121 | 8-Plant and Machine Operators and Assemblers | Male | mean | 6005 | 2958 | 3047 |
| Whittaker 2014 | NEPAL | SEARO | 7, 55 | many | 3117, 9112 | many | both | mean | 238 | 115 | 123 |
| Wu 1987 | Taiwan | WPRO | 43 | F-Construction | 9329 | 9-Elementary Occupations | Male | mean | 316 | 158 | 158 |
| Yiming 1991 | China | WPRO | 13 | B-Mining and quarrying | 8159 | 8-Plant and Machine Operators and Assemblers | Female | mean | 1101 | 886 | 215 |
| Xie 2015 | China | WPRO | 24 | B-Mining and quarrying | 7223 | 7-Craft and Related Trades Workers | both | mean | 98 | 98 |  |
| Xiao 2008 | China | WPRO |  |  |  |  | both | mean | 1906 | 953 | 953 |
| Xue 2018 | China | WPRO | 29 | B-Mining and quarrying |  |  | Male | mean | 1813 | 700 | 1113 |
| Yu 2017 | China | WPRO | 24 | B-Mining and quarrying | 7213 Sheet-metal workers | 7-Craft and Related Trades Workers |  | mean | 6297 | 6112 | 185 |
| Yuan 2015 | China | WPRO | 29 | B-Mining and quarrying | 2146 | 2-Professionals | both | mean | 396 | 215 | 181 |
| Yuan 2005 | China | WPRO | 32 Other manufacturing |  | 7221, 4110 | many | Male | mean | 174 | 88 | 86 |
